# Adaptation and validation of a coding algorithm for the Charlson Comorbidity Index in administrative claims data using the SNOMED CT standardized vocabulary

**DOI:** 10.1186/s12911-022-02006-1

**Published:** 2022-10-07

**Authors:** Stephen P. Fortin, Jenna Reps, Patrick Ryan

**Affiliations:** Janssen Research & Development, LLC, Observational Health Data Analytics, 920 U.S. Highway 202, Raritan, NJ 08869 USA

**Keywords:** Charlson comorbidity index, SNOMED, Common data model, Quan, Standardized vocabulary, Validation, OHDSI

## Abstract

**Objectives:**

The Charlson comorbidity index (CCI), the most ubiquitous comorbid risk score, predicts one-year mortality among hospitalized patients and provides a single aggregate measure of patient comorbidity. The Quan adaptation of the CCI revised the CCI coding algorithm for applications to administrative claims data using the International Classification of Diseases (ICD). The purpose of the current study is to adapt and validate a coding algorithm for the CCI using the SNOMED CT standardized vocabulary, one of the most commonly used vocabularies for data collection in healthcare databases in the U.S.

**Methods:**

The SNOMED CT coding algorithm for the CCI was adapted through the direct translation of the Quan coding algorithms followed by manual curation by clinical experts. The performance of the SNOMED CT and Quan coding algorithms were compared in the context of a retrospective cohort study of inpatient visits occurring during the calendar years of 2013 and 2018 contained in two U.S. administrative claims databases. Differences in the CCI or frequency of individual comorbid conditions were assessed using standardized mean differences (SMD). Performance in predicting one-year mortality among hospitalized patients was measured based on the c-statistic of logistic regression models.

**Results:**

For each database and calendar year combination, no significant differences in the CCI or frequency of individual comorbid conditions were observed between vocabularies (SMD ≤ 0.10). Specifically, the difference in CCI measured using the SNOMED CT vs. Quan coding algorithms was highest in MDCD in 2013 (3.75 vs. 3.6; SMD = 0.03) and lowest in DOD in 2018 (3.93 vs. 3.86; SMD = 0.02). Similarly, as indicated by the c-statistic, there was no evidence of a difference in the performance between coding algorithms in predicting one-year mortality (SNOMED CT vs. Quan coding algorithms, range: 0.725–0.789 vs. 0.723–0.787, respectively). A total of 700 of 5,348 (13.1%) ICD code mappings were inconsistent between coding algorithms. The most common cause of discrepant codes was multiple ICD codes mapping to a SNOMED CT code (*n* = 560) of which 213 were deemed clinically relevant thereby leading to information gain.

**Conclusion:**

The current study repurposed an important tool for conducting observational research to use the SNOMED CT standardized vocabulary.

**Supplementary Information:**

The online version contains supplementary material available at 10.1186/s12911-022-02006-1.

## Introduction

In observational research, measurements of patient disease burden and clinical prognosis are essential to describing study populations and adjusting for baseline clinical characteristics. Comorbid risk scores, widely accepted and applied in practice, provide a single aggregate measure of relevant comorbidities. The Charlson comorbidity index (CCI), the most ubiquitous comorbid risk score, provides a weighted index of 17 comorbid conditions to predict one-year mortality among hospitalized patients. Originally developed based on medical chart reviews of 559 patients at a single hospital, comorbid condition identification was based on the manual review of patient healthcare records [1].

Since its inception in 1984, multiple adaptations of the CCI have emerged. Notably, Deyo, Romano, and D’Hoore independently revised the CCI coding algorithm for application to administrative claims data using the International Classification of Diseases, Ninth Revision (ICD-9), and its clinical modification (ICD-9-CM) [2–4]. Subsequently, Quan translated the Deyo adaptation ICD-9-CM coding algorithm to the International Classification of Diseases, Tenth Revision (ICD-10). In the process, Quan produced an enhanced ICD-9-CM coding algorithm through the back translation of the ICD-10 coding algorithm [5]. Similarly, in 2019, Metcalfe developed and validated a coding algorithm for the CCI using Read codes through the translation of the Deyo adaptation of the CCI [6].

Although multiple vocabularies have been adopted as standards for data collection across healthcare databases, the two most commonly used vocabularies in the U.S. are ICD-10-CM and SNOMED CT. One of the primary advantages of SNOMED CT, a standardized vocabulary which maps to international coding systems, is improved consistency in research conducted across data sources containing disparate medical coding systems [6]. Furthermore, standardized vocabularies facilitate the performance of research across international federated research networks. As such, international efforts to group source vocabularies to SNOMED CT are currently ongoing and being led by organizations such as Observational Health Data Sciences and Informatics (OHDSI).

The OHDSI community has developed and implemented a coding algorithm for the CCI using SNOMED CT, henceforth referred to as the OHDSI adaptation. Although the OHDSI adaptation has been applied across several major studies, to our knowledge, no prior literature exists validating the OHDSI adaptation and recent research has shown significant discrepancies in patient identification between the OHDSI and Quan adaptations across multiple comorbid conditions comprising the CCI [7, 8]. Although SNOMED CT permits for the efficient mapping of standardized code sets to international coding systems, the conversion of code sets using non-standardized vocabularies to SNOMED CT requires careful manual curation [9]. As such, the current study describes the adaptation of SNOMED CT code sets for each comorbid condition comprising the CCI through the direct translation of the Quan coding algorithms and subsequent manual curation by clinical subject matter experts. Finally, the performance of the SNOMED CT and Quan coding algorithms are compared in the context of a retrospective cohort study of inpatient visits contained in two large U.S. administrative claims databases.

## Material and methods

### Study design and data sources

We conducted a retrospective cohort study of patients contained within two administrative claims databases in the United States; specifically, Optum® De-Identified Clinformatics Data Mart Database – Date of Death (DOD); and IBM® MarketScan® Multi-State Medicaid Database (MDCD).

DOD is comprised of inpatient visit, outpatient visit and outpatient pharmacy claims data from over 80 million privately insured patients, who are fully insured by commercial, administrative services only (ASO) or Medicare Advantage plans. In DOD, death records are derived from the Death Master File maintained by the Social Security Office. MDCD includes hospital discharge records, outpatient diagnoses and procedures, and outpatient pharmacy claims from over 31 million Medicaid enrollees located across select geographically dispersed states. In MDCD, death data is captured from the discharge status field.

All data were standardized to the Observational Health and Data Sciences and Informatics (OHDSI) Observational Medical Outcomes Partnership (OMOP) Common Data Model (CDM) version 5.3 with the July 2021 SNOMED CT International Edition Release [10]. Pursuant to Title 45 Code of Federal Regulations, Part 46 of the United States, specifically 45 CFR 46.104 (d)(4), retrospective analyses conducted in the DOD and MDCD are considered exempt from informed consent and institutional review board (IRB) approval in the United States.

### Study population

We identified patients aged ≥ 18 years with an inpatient visit occurring between January 1, 2013 to December 31, 2013 or January 1, 2018 to December 31, 2018. The calendar years of 2013 and 2018 were selected for the current study as they represented time periods occurring prior to and after the ICD-9 to ICD-10 transition, respectively, which occurred on October 1, 2015. For each patient, index was defined as the earliest observed inpatient visit for a given calendar year. The study was limited to patients with a minimum of 365 days of continuous observation within the database prior to index, and the study population was stratified by calendar year.

### Coding algorithms

#### Quan coding algorithm for Charlson comorbidity index

The current study considered both the Quan enhanced ICD-9-CM and ICD-10 coding algorithms [5]. As the current study was performed using U.S. administrative claims data, the Quan ICD-10 coding algorithm was directly translated to ICD-10-CM, and, subsequently, reviewed by clinical subject matter experts.

#### Adaptation of SNOMED CT coding algorithm for the Charlson comorbidity index

We adapted SNOMED CT code sets for each of the 17 comorbidities comprising the CCI using the following steps:

- *Step 1* An initial SNOMED CT code set was generated by directly mapping diagnosis codes included in the Quan ICD-9/10-CM coding algorithms to SNOMED CT.
- *Step 2* The SNOMED CT code set was mapped back to ICD-9/10-CM and compared to the Quan ICD-9/10-CM code sets. All discrepant codes, defined as ICD-9/10-CM codes not mapping to both the SNOMED CT and Quan coding algorithms, were identified.
- *Step 3* All SNOMED CT codes mapping to a discrepant code were vetted for inclusion by clinical subject matter experts. Specifically, the clinical relevance of the ICD-9/10-CM codes mapping to each SNOMED CT code and the impact of removing the SNOMED CT code on patient identification was carefully assessed.

### Measurement of patient characteristics

We measured patient age and sex at index. Patient baseline comorbidity was assessed based on all diagnosis codes recorded at or within 365 days prior to index. Specifically, the SNOMED CT and Quan coding algorithms were used to measure the CCI and 17 comorbid conditions comprising the CCI (myocardial infarction [MI], congestive heart failure [CHF], peripheral vascular disease [PVD], cerebrovascular disease [CVD], dementia, chronic pulmonary disease, rheumatic disease, peptic ulcer disease [PUD], mild liver disease, diabetes with vs. without chronic complications, hemiplegia and paraplegia, renal disease, malignancy, moderate or severe liver disease, metastatic solid tumor, and AIDS/HIV).

Analyses were stratified by calendar year. As such, the Quan ICD-9-CM and ICD-10-CM coding algorithms were applied to source codes for the calendar years of 2013 and 2018, respectively. In contrast, the SNOMED CT coding algorithm was applied to standard codes for both calendar years. Following the conventions outlined by Quan, the CCI was calculated as a weighted score of patient baseline comorbid conditions [5]. A complete list of SNOMED CT code sets used to query the database is available in Additional file 1: Appendix A.

### Statistical analyses

The distribution of all aforementioned patient characteristics stratified by data source and calendar year was described using descriptive statistics. Standardized mean differences (SMD) were used to assess balance in measurements of patient baseline comorbidity between the SNOMED CT and Quan coding algorithms where a SMD less than 0.10 was considered balanced. For each comorbid condition, we counted the number of patients identified by only the SNOMED CT, Quan, neither, or both coding algorithms.

The performance of coding algorithms to predict one-year mortality among hospitalized patients was assessed as described in Quan et al. using two logistic regression models [5]. In each model, the independent variable was one-year mortality and the dependent variable was the CCI. The CCI was measured using the SNOMED CT and Quan coding algorithms in models 1 and 2, respectively. The c statistic, defined as the area under the curve of the operating characteristics curve, was used to measure the predictive performance of each model.

**Fig. 1:**
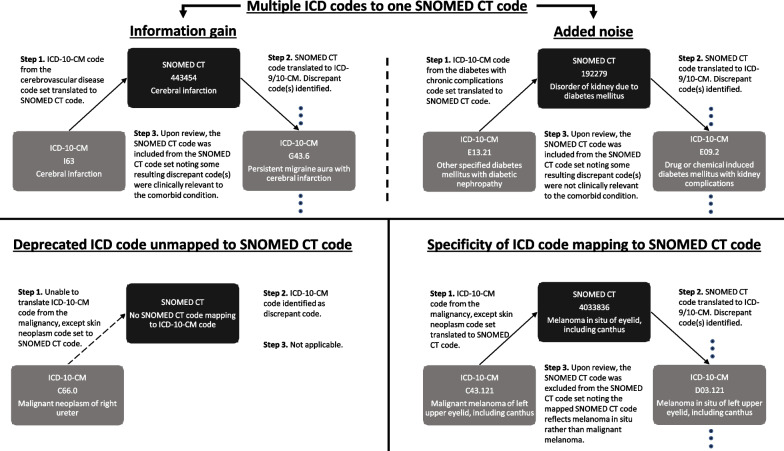
Categorization of discrepant codes during code mapping diagnostics

Finally, we examined the overlap in ICD-9/10-CM diagnosis codes mapping from the SNOMED CT and Quan coding algorithms for each individual comorbidity. Specifically, we counted the number of individual ICD-9/10-CM diagnosis codes mapped from only the SNOMED CT, Quan or both code sets for each respective comorbid condition. Code mapping diagnostics were produced for each observed discrepant ICD-9/10-CM code. As shown in Fig. 1, we categorized discrepant codes into the following categories: multiple ICD codes mapping to one SNOMED CT codes, deprecated ICD codes unmapped to SNOMED CT codes, and lack of specificity of SNOMED CT to ICD code mapping. We further differentiated clinically relevant and irrelevant discrepant codes due to multiple ICD codes mapping to one SNOMED CT code as information gain or added noise, respectively.

## Results

### Study population

The eligible study population consisted of 1,133,447 (MDCD: 328,740; and DOD: 804,707) and 1,600,700 (MDCD: 491,311; and DOD: 1,109,389) patients with an inpatient visit in 2013 and 2018, respectively. Patient baseline characteristics are summarized in Table 1. As indicated by a SMD less than 0.10, no significant imbalances in patient comorbidities were observed between coding algorithms.

**Table 1:** Patient baseline characteristics

Covariate	2013						2018					
	MDCD (*N* = 328,740)			DOD (*N* = 804,707)			MDCD (*N* = 491,311)			DOD (*N* = 1,109,389)		
	SNOMED CT	Quan^a^	SMD	SNOMED CT	Quan^a^	SMD	SNOMED CT	Quan^b^	SMD	SNOMED CT	Quan^b^	SMD
Age, years, mean (sd)	53.51 (20.97)		–	64.05 (19.29)		–	46.85 (19.83)		–	64.24 (18.14)		–
*Sex, n (%)*												
Male	93,772 (28.5)		–	327,921 (40.8)		–	154,671 (31.5)		–	467,011 (42.1)		–
Female	234,968 (71.5)		–	476,786 (59.2)		–	336,640 (68.5)		–	642,378 (57.9)		–
Charlson comorbidity index, mean (sd)	3.75 (3.66)	3.6 (3.56)	0.03	3.63 (3.62)	3.51 (3.55)	0.02	4.04 (4)	3.91 (3.92)	0.02	4.55 (3.93)	4.43 (3.86)	0.02
*Comorbid Conditions, n (%)*												
Myocardial infraction	42,067 (12.8)	42,067 (12.8)	0	111,478 (13.9)	111,478 (13.9)	0	72,069 (14.7)	72,057 (14.7)	0	192,333 (17.3)	192,333 (17.3)	0
Congestive heart failure	83,481 (25.4)	83,482 (25.4)	0	173,183 (21.5)	173,175 (21.5)	0	129,372 (26.3)	129,712 (26.4)	0	320,600 (28.9)	321,007 (28.9)	0
Peripheral vascular disease	73,630 (22.4)	73,381 (22.3)	0	212,834 (26.5)	212,337 (26.4)	0	123,268 (25.1)	121,219 (24.7)	0.01	383,111 (34.5)	378,005 (34.1)	0.01
Cerebrovascular disease	83,041 (25.3)	83,030 (25.3)	0	227,055 (28.2)	227,045(28.2)	0	121,917 (24.8)	121,824 (24.8)	0	351,730 (31.7)	351,659 (31.7)	0.00
Dementia	36,021 (11)	26,351 (8)	0.07	65,097 (8.1)	46,465 (5.8)	0.06	49,312 (10)	47,300 (9.6)	0.01	127,958 (11.5)	125,035 (11.3)	0.01
Chronic pulmonary disease	167,349 (50.9)	167,165(50.9)	0	328,588 (40.8)	328,129(40.8)	0	261,239 (53.2)	261,161 (53.2)	0	491,536 (44.3)	491,425 (44.3)	0
Rheumatic disease	22,664 (6.9)	21,326 (6.5)	0.01	64,375(8)	59,095 (7.3)	0.02	37,012 (7.5)	32,366 (6.6)	0.03	107,692 (9.7)	92,573 (8.3)	0.04
Peptic ulcer disease	21,419 (6.5)	21,419 (6.5)	0	45,029 (5.6)	45,029 (5.6)	0	33,855 (6.9)	33,855 (6.9)	0	70,761 (6.4)	70,761 (6.4)	0
Mild liver disease	49,682 (15.1)	48,863 (14.9)	0.01	116,317 (14.5)	115,402 (14.3)	0	103,234 (21)	100,175 (20.4)	0.01	198,534 (17.9)	193,778 (17.5)	0.01
Diabetes without chronic complications	123,552 (37.6)	123,547 (37.6)	0	268,579 (33.4)	268,552 (33.4)	0	180,707 (36.8)	178,911 (36.4)	0.01	44,1097 (39.8)	436,760 (39.4)	0.01
Diabetes with chronic complications	66,637 (20.3)	54,233 (16.5)	0.06	136,618 (17)	117,527 (14.6)	0.04	123,869 (25.2)	111,701 (22.7)	0.04	293,174 (26.4)	267,237 (24.1)	0.04
Hemiplegia or paraplegia	23,748 (7.2)	23,420 (7.1)	0	30,802 (3.8)	30,170 (3.8)	0	41,778 (8.5)	40,428 (8.2)	0.01	60,555 (5.5)	58,900 (5.3)	0.01
Renal disease	69,341 (21.1)	65,131(19.8)	0.02	170,506 (21.2)	161,663 (20.1)	0.02	106,497 (21.7)	102,761 (20.9)	0.01	330,026 (29.8)	325,401 (29.3)	0.01
Malignancy, except skin neoplasms	40,114 (12.2)	38,912 (11.8)	0.01	161,419 (20.1)	158,319 (19.7)	0.01	60,499 (12.3)	58,426 (11.9)	0.01	247,915 (22.4)	241,187 (21.7)	0.01
Moderate or severe liver disease	7,262 (2.2)	7,262 (2.2)	0	10,887 (1.4)	10,881 (1.4)	0	15,374 (3.1)	13,194 (2.7)	0.02	24,232 (2.2)	19,882 (1.8)	0.02
Metastatic solid tumor	12,461 (3.8)	12,248 (3.7)	0	42,841 (5.3)	42,151 (5.2)	0	19,762 (4)	18,820 (3.8)	0.01	66,772 (6)	64,521 (5.8)	0.01
AIDS/HIV	5,437 (1.7)	5,437 (1.7)	0	3,124 (0.4)	3,124 (0.4)	0	7,471 (1.5)	7,471 (1.5)	0	4,850 (0.4)	4,850 (0.4)	0

^**a**^Quan enhanced International Classification of Diseases, Ninth Edition, Clinical Modification coding algorithm

^**b**^Quan enhanced International Classification of Diseases, Tenth Edition, Clinical Modification coding algorithm

Among patients meeting the study criteria in 2013, the average age was 53.5 (sd = 21.0) years and just over a quarter of patients were male (28.5%) in MDCD. In DOD, the average age was 64.1 (sd = 19.3) years and 40.8% of patients were male. The CCI was slightly higher, albeit non-significantly, with the SNOMED CT coding algorithm as compared to Quan ICD-9-CM coding algorithm (MDCD: 3.75 vs. 3.6, SMD = 0.029; and DOD: 3.63 vs. 3.51, SMD = 0.024). Approximately a quarter of patients had the following comorbidities in either database: CHF, PVD, CVD, chronic pulmonary disease, and diabetes without chronic complications.

In 2018, the average age of patients was 46.9 (sd = 19.8) years and 31.5% were male in MDCD. As compared to MDCD, with an average age of 64.24 (sd = 18.14) years, patients in DOD were older and a higher proportion were male (42.1%). The CCI was comparable between the SNOMED CT versus Quan coding algorithms (MDCD: 4.04 vs. 3.91, SMD = 0.029; and DOD: 3.93 vs. 3.86, SMD = 0.024). Over a quarter of patients were identified as having the following comorbidities in either MDCD or DOD: CHF, PVD, CVD, chronic pulmonary disease, diabetes with and without chronic complications, and renal disease.

### Patient comorbidity overlap

The overlap in patients identified for each comorbidity comprising the CCI using SNOMED CT versus Quan ICD-9/10-CM coding algorithms is shown in Table 2. In 2013, over 1% of patients were identified by only the SNOMED CT coding algorithm in both databases for the following comorbidities: dementia, rheumatic disease, diabetes with chronic complications, and renal disease. Similarly, over 1% of patients were identified as having rheumatic disease and diabetes with chronic complications by only the SNOMED CT coding algorithm in 2018. Fewer than 0.1% of patients were identified by only the Quan ICD-9/10-CM coding algorithms for all comorbidities.

**Table 2:** Overlap in patient comorbid conditions comprising CCI between coding algorithms

Comorbid Conditions, *n* (%)	2013								2018							
	MDCD (*N* = 328,740)				DOD (*N* = 804,707)				MDCD (*N* = 491,311)				DOD (*N* = 1,109,389)			
	Both	Neither	SNOMED CT only	Quan only^a^	Both	Neither	SNOMED CT only	Quan only^a^	Both	Neither	SNOMED CT Only	Quan Only^b^	Both	Neither	SNOMED CT Only	Quan only^b^
Myocardial infraction	42,067 (12.8%)	286,673 (87.2%)	0 (0%)	0 (0%)	111,478 (13.9%)	693,229 (86.1%)	0 (0%)	0 (0%)	72,057 (14.7%)	419,242 (85.3%)	12 (0%)	0 (0%)	192,333 (17.3%)	917,056 (82.7%)	0 (0%)	0 (0%)
Congestive heart failure	83,481 (25.4%)	245,258 (74.6%)	0 (0%)	1 (0%)	173,175 (21.5%)	631,524 (78.5%)	8 (0%)	0 (0%)	129,371 (26.3%)	361,598 (73.6%)	1 (0%)	341 (0.1%)	320,597 (28.9%)	788,379 (71.1%)	3 (0%)	410 (0%)
Peripheral vascular disease	73,381 (22.3%)	255,110 (77.6%)	249 (0.1%)	0 (0%)	212,337 (26.4%)	591,873 (73.6%)	497 (0.1%)	0 (0%)	121,219 (24.7%)	368,043 (74.9%)	2,049 (0.4%)	0 (0%)	378,005 (34.1%)	726,278 (65.5%)	5,106 (0.5%)	0 (0%)
Cerebrovascular disease	83,030 (25.3%)	245,699 (74.7%)	11 (0%)	0 (0%)	227,045 (28.2%)	577,652 (71.8%)	10 (0%)	0 (0%)	121,824 (24.8%)	369,394 (75.2%)	93 (0%)	0 (0%)	351,659 (31.7%)	757,659 (68.3%)	71 (0%)	0 (0%)
Dementia	26,351 (8%)	292,719 (89%)	9,670 (2.9%)	0 (0%)	46,465 (5.8%)	739,610 (91.9%)	18,632 (2.3%)	0 (0%)	47,300 (9.6%)	441,999 (90%)	2,012 (0.4%)	0 (0%)	125,035 (11.3%)	981,431 (88.5%)	2,923 (0.3%)	0 (0%)
Chronic pulmonary disease	167,148 (50.8%)	161,374 (49.1%)	201 (0.1%)	17 (0%)	328,056 (40.8%)	476,046 (59.2%)	532 (0.1%)	73 (0%)	261,150 (53.2%)	230,061 (46.8%)	89 (0%)	11 (0%)	491,359 (44.3%)	617,787 (55.7%)	177 (0%)	66 (0%)
Rheumatic disease	21,326 (6.5%)	306,076 (92.1%)	1,338 (1.5%)	0 (0%)	59,090 (7.3%)	740,327 (90.2%)	5,285 (2.4%)	5 (0%)	32,366 (6.6%)	454,299 (91.7%)	4,646 (1.7%)	0 (0%)	92,570 (8.3%)	1,001,694 (89.3%)	15,122 (2.4%)	3 (0%)
Peptic ulcer disease	21,419 (6.5%)	307,321 (93.5%)	0 (0%)	0 (0%)	45,029 (5.6%)	759,678 (94.4%)	0 (0%)	0 (0%)	33,855 (6.9%)	457,456 (93.1%)	0 (0%)	0 (0%)	70,761 (6.4%)	1,038,628 (93.6%)	0 (0%)	0 (0%)
Mild liver disease	48,863 (14.9%)	279,058 (84.9%)	819 (0.2%)	0 (0%)	115,402 (14.3%)	688,390 (85.5%)	915 (0.1%)	0 (0%)	100,175 (20.4%)	388,077 (79%)	3,059 (0.6%)	0 (0%)	193,778 (17.5%)	910,855 (82.1%)	4,756 (0.4%)	0 (0%)
Diabetes without chronic complications	12,3547 (37.6%)	205,188 (62.4%)	5 (0%)	0 (0%)	268,552 (33.4%)	536,128 (66.6%)	27 (0%)	0 (0%)	178,907 (36.4%)	310,600 (63.2%)	1,800 (0.4%)	4 (0%)	436,702 (39.4%)	668,234 (60.2%)	4,395 (0.4%)	58 (0%)
Diabetes with chronic complications	54,233 (16.5%)	262,103 (79.7%)	12,404 (3.8%)	0 (0%)	117,527 (14.6%)	668,089 (83%)	19,091 (2.4%)	0 (0%)	111,701 (22.7%)	367,442 (74.8%)	12,168 (2.5%)	0 (0%)	267,237 (24.1%)	816,215 (73.6%)	25,937 (2.3%)	0 (0%)
Hemiplegia or paraplegia	23,420 (7.1%)	304,992 (92.8%)	328 (0.1%)	0 (0%)	30,170 (3.7%)	773,905 (96.2%)	632 (0.1%)	0 (0%)	40,428 (8.2%)	449,533 (91.5%)	1,350 (0.3%)	0 (0%)	58,900 (5.3%)	1,048,834 (94.5%)	1,655 (0.1%)	0 (0%)
Renal disease	65,131 (19.8%)	259,399 (78.9%)	4,210 (1.3%)	0 (0%)	161,663 (20.1%)	634,201 (78.8%)	8,843 (1.1%)	0 (0%)	102,761 (20.9%)	384,814 (78.3%)	3,736 (0.8%)	0 (0%)	325,401 (29.3%)	779,363 (70.3%)	4,625 (0.4%)	0 (0%)
Malignancy, except skin neoplasms	38,912 (11.8%)	288,626 (87.8%)	1,202 (0.4%)	0 (0%)	158,319 (19.7%)	643,288 (79.9%)	3,100 (0.4%)	0 (0%)	58,426 (11.9%)	430,812 (87.7%)	2,073 (0.4%)	0 (0%)	241,187 (21.7%)	861,474 (77.7%)	6,728 (0.6%)	0 (0%)
Moderate or severe liver disease	7,262 (2.2%)	321,478 (97.8%)	0 (0%)	0 (0%)	10,881 (1.4%)	793,820 (98.6%)	6 (0%)	0 (0%)	13,194 (2.7%)	475,937 (96.9%)	2,180 (0.4%)	0 (0%)	19,882 (1.8%)	1,085,157 (97.8%)	4,350 (0.4%)	0 (0%)
Metastatic solid tumor	12,248 (3.7%)	316,279 (96.2%)	213 (0.1%)	0 (0%)	42,151 (5.2%)	761,866 (94.7%)	690 (0.1%)	0 (0%)	18,820 (3.8%)	471,549 (96%)	942 (0.2%)	0 (0%)	64,521 (5.8%)	1,042,617 (94%)	2,251 (0.2%)	0 (0%)
AIDS/HIV	5,437 (1.7%)	323,303 (98.3%)	0 (0%)	0 (0%)	3,124 (0.4%)	801,583 (99.6%)	0 (0%)	0 (0%)	7,471 (1.5%)	483,840 (98.5%)	0 (0%)	0 (0%)	4,850 (0.4%)	1,104,539 (99.6%)	0 (0%)	0 (0%)

^a^Quan enhanced International Classification of Diseases, Ninth Edition, Clinical Modification coding algorithm

^b^Quan enhanced International Classification of Diseases, Tenth Edition, Clinical Modification coding algorithm

### Predictive performance

In MDCD, the frequency of one-year mortality was 5.0% (*N* = 16,412) and 4.9% (*N* = 24,017) in 2013 and 2018, respectively. Meanwhile, the frequency of one-year mortality was 10.3% (*N* = 82,819) and 13.1% (*N* = 145,516) in 2013 and 2018, respectively, in DOD. For each calendar year and database combination, as indicated by the c-statistic, no significant difference in the performance of models 1 versus 2 (MDCD, 2013: 0.725 vs. 0.723; DOD, 2013: 0.789 vs. 0.787; MDCD, 2018: 0.754 vs. 0.752; and DOD, 2018: 0.757 vs. 0.757) to predict one-year mortality was observed. Furthermore, the performance of models was database dependent; a statistically significant improvement in performance was observed in DOD as compared to MDCD in both 2013 and 2018. The performance of each model, including 95% confidence intervals, is further described in Additional file 1: Appendix B.

### Code mapping overlap

The degree of overlap in ICD-9/10-CM diagnosis codes mapping to each comorbidity comprising the CCI between the SNOMED CT and Quan coding algorithms is shown in Table 3. A total of 5,343 diagnosis codes (ICD-9-CM: 1,500; and ICD-10-CM: 3,843) mapped to either coding algorithm of which 4,648 (87.0%) were consistent between algorithms. Among discrepant codes, 553 (ICD-9-CM: 110; and ICD-10-CM: 443) and 147 (ICD-9-CM: 4; and ICD-10-CM: 138) diagnosis codes mapped to only the SNOMED CT and Quan coding algorithms, respectively.

**Table 3:** Overlap in mapping of diagnosis codes for each comorbidity comprising the CCI between coding algorithms

Comorbid condition	ICD-9-CM, *n* (%)			ICD-10-CM, *n* (%)		
	Both	SNOMED CT only^a^	Quan only^a^	Both	SNOMED CT Only^a^	Quan only^a^
Myocardial infraction	42 (100%)	0 (0%)	0 (0%)	24 (92.3%)	2 (7.7%)	0 (0%)
Congestive heart failure	33 (97.1%)	1 (2.9%)	0 (0%)	40 (93%)	1 (2.3%)	2 (4.7%)
Peripheral vascular disease	48 (90.6%)	4 (7.5%)	1 (1.9%)	319 (94.4%)	13 (3.8%)	6 (1.8%)
Cerebrovascular disease	91 (94.8%)	5 (5.2%)	0 (0%)	531 (96.9%)	17 (3.1%)	0 (0%)
Dementia	22 (84.6%)	4 (15.4%)	0 (0%)	18 (100%)	0 (0%)	0 (0%)
Chronic pulmonary disease	58 (95.1%)	2 (3.3%)	1 (1.6%)	85 (97.7%)	1 (1.1%)	1 (1.1%)
Rheumatic disease	14 (82.4%)	2 (11.8%)	1 (5.9%)	465 (78.5%)	125 (21.1%)	2 (0.3%)
Peptic ulcer disease	112 (100%)	0 (0%)	0 (0%)	40 (100%)	0 (0%)	0 (0%)
Mild liver disease	27 (73%)	9 (24.3%)	1 (2.7%)	48 (80%)	12 (20%)	0 (0%)
Diabetes without chronic complications	30 (81.1%)	7 (18.9%)	0 (0%)	72 (75%)	21 (21.9%)	3 (3.1%)
Diabetes with chronic complications	20 (35.7%)	36 (64.3%)	0 (0%)	270 (60.7%)	175 (39.3%)	0 (0%)
Hemiplegia or paraplegia	48 (87.3%)	7 (12.7%)	0 (0%)	55 (83.3%)	11 (16.7%)	0 (0%)
Renal disease	46 (79.3%)	12 (20.7%)	0 (0%)	34 (65.4%)	18 (34.6%)	0 (0%)
Malignancy, except skin neoplasms	738 (98.1%)	14 (1.9%)	0 (0%)	1177 (88.4%)	30 (2.3%)	124 (9.3%)
Moderate or severe liver disease	9 (100%)	0 (0%)	0 (0%)	21 (80.8%)	5 (19.2%)	0 (0%)
Metastatic solid tumor	47 (87%)	7 (13%)	0 (0%)	62 (83.8%)	12 (16.2%)	0 (0%)
AIDS/HIV	1 (100%)	0 (0%)	0 (0%)	1 (100%)	0 (0%)	0 (0%)

^a^Number of diagnosis codes mapping only to the respective coding algorithm

*ICD-9/10-CM* International Classification of Diseases, Ninth and Tenth Revision, Clinical Modification; *CCI* Charlson comorbidity index

Perfect overlap in diagnosis codes was observed for the following comorbidities: ICD-9-CM, MI, PUD, moderate or severe liver disease, and AIDS/HIV; and ICD-10-CM, dementia, PUD and AIDS/HIV. On the other hand, comorbidities with less than 80% overlap in diagnosis codes between adaptations included: rheumatic disease (ICD-9-CM: 82.4%; ICD-10-CM: 78.5%); mild liver disease (ICD-9-CM: 73.0%; ICD-10-CM: 80.0%); diabetes without chronic complication (ICD-9-CM: 81.1%; ICD-10-CM: 75%); diabetes with chronic complication (ICD-9-CM: 35.7%; ICD-10-CM: 60.7%); and renal disease (ICD-9-CM: 79.3%; ICD-10-CM: 65.4%).

### Code mapping diagnostics

The cause of all discrepant ICD-9/10-CM codes mapping to each comorbid condition is summarized in Table 4.

**Table 4:** Code mapping diagnostics of discrepant ICD-9/10-CM codes between coding algorithms

Comorbid condition	Multiple ICD codes to one SNOMED CT code		Deprecated ICD code unmapped to SNOMED CT code	Specificity of SNOMED CT to ICD code mapping
	Information gain^a^	Added noise^b^		
Myocardial infraction	2	0	0	0
Congestive heart failure	0	3	0	1
Peripheral vascular disease	15	9	0	0
Cerebrovascular disease	22	0	0	0
Dementia	4	0	0	0
Chronic pulmonary disease	3	2	0	0
Rheumatic disease	9	121	0	0
Peptic ulcer disease	0	0	0	0
Mild liver disease	20	1	0	1
Diabetes without chronic complications	5	23	3	0
Diabetes with chronic complications	30	181	0	0
Hemiplegia or paraplegia	6	12	0	0
Renal disease	18	12	0	0
Malignancy, except skin neoplasms	2	36	120	10
Moderate or severe liver disease	2	3	0	0
Metastatic solid tumor	0	19	0	0
AIDS/HIV	0	0	0	0
Total	138	422	123	12

^a^Information gain: Discrepant code mapping to clinically relevant code

^b^Add noise: Discrepant code mapping to clinically irrelevant code

*ICD-9/10-CM* International Classification of Diseases, Ninth and Tenth Revision, Clinical Modification

#### Multiple ICD codes to one SNOMED CT code

Accounting for 80.6% (560 of 695) discrepant codes, the mapping of 2 or more ICD-9/10-CM codes to a single SNOMED CT standard code was the primary source of discrepancies between coding algorithms. The frequency of such translation errors was especially pronounced among the code sets for rheumatic disease (*n* = 130) and diabetes with chronic complications (*n* = 211).

Approximately 24.6% (138 of 560) of these discrepant codes were not included in the Quan coding algorithms but, nevertheless, represented clinical conditions associated with their respective comorbid condition. The additional capture of these codes was due to the mapping of multiple ICD-9/10-CM codes to a single SNOMED CT code and led to information gain among the SNOMED CT coding algorithm. Information gain was most prevalent among the code set for diabetes with chronic complications (*n* = 30), CVD (*n* = 22), and mild liver disease (*n* = 20). For example, the SNOMED CT code 443,454 (cerebral infarction) mapped to ICD-10-CM codes G43.6X associated with persistent migraine aura with cerebral infarction, which were not included in the Quan code set for cerebrovascular disease. As such, the SNOMED CT coding algorithm mapped to an additional ICD-10-CM codes for cerebrovascular disease leading to information gain.

On the other hand, 422 of these discrepant codes were deemed to contribute added noise to the code set for their respective comorbid condition. For instance, the SNOMED CT code 192,279 (disorder of kidney due to diabetes mellitus) mapped to 15 ICD-9/10-CM codes. While 7 of these diagnosis codes (250.4, 250.4X, E13.2 and E13.2X) were included in the Quan code sets for diabetes with chronic complications, this led to the additional capture of conditions associated with secondary diabetes (249.4 and 249.4X) and drug or chemical induced diabetes (E09.2 and E09.2X) with renal manifestations by the SNOMED CT coding algorithm.

#### Deprecated ICD code unmapped to SNOMED CT code

The Quan coding algorithm contained a total of 123 deprecated ICD-10-CM codes, which were unmapped to SNOMED CT. These codes were associated with the two following comorbid conditions: malignancy, except skin neoplasms (*n* = 120) and diabetes without chronic complications (*n* = 3). No patient records containing deprecated ICD-10-CM codes were observed in either data source.

#### Specificity of ICD code mapping to SNOMED CT code

Approximately 1.7% (12 of 695) of discrepant codes were due to a lack of specificity in the mapping of ICD codes to SNOMED CT. These errors in mapping were observed among diagnosis codes contained in the code sets for CHF (*n* = 1), mild liver disease (*n* = 1), and malignancy, except skin neoplasms (*n* = 10).

For instance, ICD-10-CM code I13.2 (hypertensive heart and chronic kidney disease with heart failure and with stage 5 chronic kidney disease, or end stage renal disease) mapped to SNOMED CT code 44,784,621 (hypertensive heart and chronic kidney disease). Unfortunately, SNOMED CT code 44,784,621 also mapped to codes such as I13.1 (hypertensive heart and chronic kidney disease without heart failure) thereby making it inappropriate for inclusion in the SNOMED CT code set for CHF. However, modifying the mapping of I13.2 to SNOMED CT code 44,782,728 (hypertensive heart and chronic kidney disease with congestive heart failure) would have permitted for the capture of the code by the SNOMED CT code set.

## Discussion

The current study found no evidence of significant differences in the overall CCI, frequency of individual comorbidities, or performance in predicting one-year mortality among hospitalized patients between the newly adapted SNOMED CT and Quan coding algorithms. In contrast, prior research has shown large discrepancies in patient identification and measurement of the CCI between the OHDSI and the Quan adaptations of the CCI [7, 8]. The improved consistency in patient identification between algorithms was achieved by adapting the SNOMED CT coding algorithm directly from the Quan adaptation of the CCI. Furthermore, all discrepant codes between coding algorithms were carefully vetted by clinical subject matter experts considering the cause and potential impact of each respective discrepant code on patient identification.

While the origins of the ICD system stem from epidemiology, the roots of SNOMED CT may be traced to bioinformatics. Consequently, fundamental differences exist in the constructs of these terminologies. Whereas the ICD system is a taxonomy, SNOMED CT is an ontology and, in contrast to the ICD system, polyhierarchical. For instance, pregnancy related renal disease is classified under pregnancy, childbirth and the puerperium in the ICD system but is associated to kidney disease, disorders of pregnancy, and complications of pregnancy, childbirth and/or puerperium in SNOMED CT. Due to its polyhierarchical nature, SNOMED CT facilitates the aggregation of related concepts in the development of code-based algorithms. As it relates to the translation of coding algorithms from ICD to SNOMED CT, the difference in constructs poses both unique challenges and opportunities.

A total of 5,343 ICD-9/10-CM codes mapped to either coding algorithm among which 695 (13.0%) were inconsistent between algorithms. The primary source of discrepant codes was the mapping of multiple ICD codes to a single SNOMED CT code (*n* = 560), which was especially prevalent among the code sets for rheumatic disease (*n* = 130) and diabetes with chronic complications (*n* = 211). These discrepant codes were in part due to the presence of diagnosis codes of unspecified or not otherwise specified (e.g., unspecified nephritic syndrome) in ICD that are typically represented as higher-level terms within SNOMED CT (e.g., nephritic syndrome). In 24.6% (*n* = 138) of cases, this was associated with the additional capture of clinically relevant diagnosis codes by the SNOMED CT coding algorithm leading to information gain. Although the additional capture of these diagnosis codes represents a technical departure from the Quan adaptation, the difference may be due in part to the advantages of the SNOMED CT construct or differences in clinical opinion. Other sources of discrepant codes included a lack of mapping of deprecated ICD codes to SNOMED CT (*n* = 123), and lack of specificity in the mapping between ICD and SNOMED CT codes (*n* = 12).

Nevertheless, no significant differences in the overall CCI were observed between the SNOMED CT vs. Quan coding algorithms among inpatient visits occurring in either 2013 (MDCD: 3.75 vs. 3.6; and DOD: 3.63 vs. 3.51) or 2018 (MDCD: 4.04 vs. 3.91; and DOD: 4.55 vs. 4.43). Despite a slight increase in patient identification by the SNOMED CT code sets for dementia, renal disease, rheumatic disease and diabetes with chronic complications, no significant difference in the frequency of comorbidities comprising the CCI was observed as indicated by a SMD less than 0.1. These findings reflect the low prevalence of patient records associated with discrepant codes.

In contrast, the currently implemented OHDSI adaptation has been associated with a higher average CCI as compared to the Quan adaptation by both Fortin et al. and Viernes et al. [7, 8] Specifically, Fortin et al. found several comorbid conditions identified in over 5% of the study population by either only the OHDSI coding algorithm (chronic pulmonary disease, diabetes with chronic complications, renal disease, and malignancy) or only the Quan adaptation (peripheral vascular disease, chronic pulmonary disease, and mild liver disease) [7]. Viernes et al. hypothesized the higher average CCI was associated the mapping of the OHDSI SNOMED CT coding algorithm to additional ICD codes although the current study indicates the impact of discrepant codes on the CCI is also a function of the prevalence of each respective discrepant code observed in the study population [8].

The performance between coding algorithms in predicting one-year mortality among hospitalized patients was comparable. However, as indicated by the c-statistic, the predictive performance of the CCI fluctuated between data sources and by calendar year. It follows the impact of data source, vocabulary, and time-dependent effects on the performance of the CCI were consistent between the newly adapted SNOMED CT and Quan coding algorithms in the current study.

## Limitations

The current study was subject to limitations. First, MDCD and DOD do not contain complete capture of patient deaths. As such, the predictive performance of models may have been underestimated. However, the degree of underestimation was expected to be consistent between models thereby preserving the validity of comparisons of performance between models. Second, the SNOMED CT coding algorithm was validated in two large U.S. administrative claims databases. Estimates of predictive performance of the CCI may not be generalizable to other healthcare databases. Nevertheless, in practice, the CCI is most frequently used as a measure of disease burden as opposed to a predictor of one-year mortality. Third, new releases to SNOMED CT are published every 6 months, and, consequently, additional differences between coding algorithms for the CCI may surface over time. Although the newly proposed SNOMED CT coding algorithm represents a significant advancement in terms of transparency and reproducibility, periodic validation and update of the coding algorithm using the methods outlined in this paper may be warranted.

## Conclusion

The current study leveraged standardized vocabularies to repurpose an important tool for conducting observational research in administrative claims data. The newly adapted SNOMED CT coding algorithm possessed comparable performance to the Quan adaptation of the CCI in terms of the measurement of the CCI, patient identification across all comorbid conditions comprising the CCI, and performance in predicting one-year mortality among hospitalized patients; however, the new algorithm may be applied to standardized databases and allows for more consistent application across data sources with disparate medical coding systems. These innovations permit for improved transparency and reproducibility of observational research. Adoption of the SNOMED CT coding algorithm may be promoted through the development and implementation of data analytics tools by international research communities such as OHDSI.

## Supplementary Information

**Additional file 1. Appendix A:** A complete list of codes in the SNOMED CT coding algorithm for the Charlson comorbidity index used to query the database. **Appendix B:** Additional details on the performance of Charlson comorbidity indexcoding algorithms to predict one-year mortality.

**Additional file 2.** Review history.

## Data Availability

The data that support the findings of this study are available from IBM MarketScan® and Optum®.but restrictions apply to the availability of these data, which were used under license for the current study, and so are not publicly available. Data are however available from the authors upon reasonable request and with permission of IBM MarketScan® and Optum®. Please, contact Stephen P Fortin with any data-related requests. All authors read and approved the final manuscript.

## Abbreviations

ASO: Administrative services only
CCI: Charlson comorbidity index
CDM: Common data model
CHF: Congestive heart failure
CVD: Cerebrovascular disease
DOD: Optum® De-Identified Clinformatics Data Mart Database–Date of Death
ICD-9: International classification of diseases, ninth revision
ICD-9/10-CM: International classification of diseases, ninth and tenth revision, clinical modification
IRB: Institutional review board
MDCD: IBM® MarketScan® Multi-State Medicaid Database
MI: Myocardial infarction
OHDSI: Observational health data sciences and informatics
OMOP: Observational medical outcomes partnership
PUD: Peptic ulcer disease
PVD: Peripheral vascular disease

## Declarations

### Review History

Review history available in Additional file 2.
