# Correction to: Adaptation and validation of a coding algorithm for the Charlson Comorbidity Index in administrative claims data using the SNOMED CT standardized vocabulary

**DOI:** 10.1186/s12911-023-02205-4

**Published:** 2023-06-15

**Authors:** Stephen P. Fortin, Jenna Reps, Patrick Ryan

**Affiliations:** Observational Health Data Analytics, Janssen Research & Development, LLC, 920 U.S. Highway 202, Raritan, NJ 08869 USA

**Correction**: *BMC Med Inform Decis Mak* **22**, 261 (2022).

10.1186/s12911-022-02006-1

Following publication of the original article [1], it was reported that there was an error in the code list for ‘moderate or severe liver disease’ in Additional File 1: Appendix A. The corrected Additional file 1 is included in this Correction and the original article has been updated.

## Electronic supplementary material

Below is the link to the electronic supplementary material.

Supplementary Material 1

## References

[1] Fortin SP, Reps J, Ryan P (2022). Adaptation and validation of a coding algorithm for the Charlson Comorbidity Index in administrative claims data using the SNOMED CT standardized vocabulary. BMC Med Inform Decis Mak.

