# Supplementary material for: Adaptation and validation of a coding algorithm for the Charlson Comorbidity Index in administrative claims data using the SNOMED CT standardized vocabulary
Source: BMC Med Inform Decis Mak. 2022 Oct 7;22:261. doi: 10.1186/s12911-022-02006-1 (PMC9541054; doi:10.1186/s12911-022-02006-1)
Supplement: Supplementary file 1 — Additional file 1. Appendix A: A complete list of codes in the SNOMED CT coding algorithm for the Charlson comorbidity index used to query the database. Appendix B: Additional details on the performance of Charlson comorbidity indexcoding algorithms to predict one-year mortality. [file 12911_2022_2006_MOESM1_ESM.docx]

**Supplemental Appendix A**. List of codes in the SNOMED CT coding algorithm for the Charlson comorbidity index

| **Comorbid Condition** | **Concept ID** | **Concept Code** | **Concept Name** | **Vocabulary** |
| --- | --- | --- | --- | --- |
| Peripheral vascular disease | 4316222 | 95443002 | Venous intermittent claudication | SNOMED CT |
| Peripheral vascular disease | 192673 | 82196007 | Vascular insufficiency of intestine | SNOMED CT |
| Peripheral vascular disease | 4134603 | 128321003 | Vascular disorder of intestine | SNOMED CT |
| Peripheral vascular disease | 312939 | 52403007 | Thromboangiitis obliterans | SNOMED CT |
| Peripheral vascular disease | 433222 | 195265003 | Thoracoabdominal aortic aneurysm, ruptured | SNOMED CT |
| Peripheral vascular disease | 436996 | 233984007 | Thoracoabdominal aortic aneurysm | SNOMED CT |
| Peripheral vascular disease | 441051 | 74883004 | Thoracic aortic aneurysm without rupture | SNOMED CT |
| Peripheral vascular disease | 441875 | 195258006 | Thoracic aortic aneurysm which has ruptured | SNOMED CT |
| Peripheral vascular disease | 320646 | 12232008 | Syphilitic aneurysm of aorta | SNOMED CT |
| Peripheral vascular disease | 317305 | 68109007 | Stricture of artery | SNOMED CT |
| Peripheral vascular disease | 312343 | 73067008 | Ruptured aortic aneurysm | SNOMED CT |
| Peripheral vascular disease | 195559 | 14336007 | Ruptured abdominal aortic aneurysm | SNOMED CT |
| Peripheral vascular disease | 44782775 | 34881000119105 | Peripheral vascular disease associated with another disorder | SNOMED CT |
| Peripheral vascular disease | 321052 | 400047006 | Peripheral vascular disease | SNOMED CT |
| Peripheral vascular disease | 317309 | 399957001 | Peripheral arterial occlusive disease | SNOMED CT |
| Peripheral vascular disease | 36712807 | 12237231000119100 | Pain at rest of right lower limb co-occurrent and due to atherosclerosis | SNOMED CT |
| Peripheral vascular disease | 36712805 | 12237111000119100 | Pain at rest of left lower limb co-occurrent and due to atherosclerosis | SNOMED CT |
| Peripheral vascular disease | 4325344 | 428171009 | Pain at rest due to peripheral vascular disease | SNOMED CT |
| Peripheral vascular disease | 37312520 | 792854004 | Ischemic foot ulcer due to atherosclerosis of artery of lower limb | SNOMED CT |
| Peripheral vascular disease | 36712806 | 12237191000119100 | Intermittent claudication of right lower limb co-occurrent and due to atherosclerosis | SNOMED CT |
| Peripheral vascular disease | 36717286 | 12237071000119100 | Intermittent claudication of left lower limb co-occurrent and due to atherosclerosis | SNOMED CT |
| Peripheral vascular disease | 36717006 | 12236951000119100 | Intermittent claudication of bilateral lower limbs co-occurrent and due to atherosclerosis | SNOMED CT |
| Peripheral vascular disease | 37312529 | 792845002 | Intermittent claudication due to atherosclerosis of artery of limb | SNOMED CT |
| Peripheral vascular disease | 442774 | 63491006 | Intermittent claudication | SNOMED CT |
| Peripheral vascular disease | 321882 | 39823006 | Generalized atherosclerosis | SNOMED CT |
| Peripheral vascular disease | 36717279 | 15649901000119100 | Gangrene of right lower limb due to atherosclerosis | SNOMED CT |
| Peripheral vascular disease | 37312531 | 792843009 | Gangrene of limb due to atherosclerosis of artery of limb | SNOMED CT |
| Peripheral vascular disease | 36712963 | 15649991000119100 | Gangrene of left lower limb due to atherosclerosis | SNOMED CT |
| Peripheral vascular disease | 134380 | 37151006 | Erythromelalgia | SNOMED CT |
| Peripheral vascular disease | 432346 | 230730001 | Dissection of vertebral artery | SNOMED CT |
| Peripheral vascular disease | 37016882 | 713029000 | Dissection of thoracoabdominal aorta | SNOMED CT |
| Peripheral vascular disease | 436136 | 233994002 | Dissection of thoracic aorta | SNOMED CT |
| Peripheral vascular disease | 37016889 | 713036004 | Dissection of renal artery | SNOMED CT |
| Peripheral vascular disease | 201043 | 233999007 | Dissection of iliac artery | SNOMED CT |
| Peripheral vascular disease | 37109512 | 720626009 | Dissection of carotid artery | SNOMED CT |
| Peripheral vascular disease | 46272492 | 710864009 | Dissection of artery | SNOMED CT |
| Peripheral vascular disease | 320739 | 308546005 | Dissection of aorta | SNOMED CT |
| Peripheral vascular disease | 4256889 | 408666009 | Dissection of abdominal aorta | SNOMED CT |
| Peripheral vascular disease | 199064 | 111354009 | Chronic vascular insufficiency of intestine | SNOMED CT |
| Peripheral vascular disease | 44782819 | 698816006 | Chronic occlusion of artery of extremity | SNOMED CT |
| Peripheral vascular disease | 35611566 | 12236991000119100 | Bilateral lower limb atherosclerosis pain at rest co-occurrent and due to atherosclerosis | SNOMED CT |
| Peripheral vascular disease | 35615028 | 15649941000119100 | Bilateral atherosclerosis of lower limbs with gangrene | SNOMED CT |
| Peripheral vascular disease | 195834 | 45281005 | Atherosclerosis of renal artery | SNOMED CT |
| Peripheral vascular disease | 40484551 | 442701004 | Atherosclerosis of nonautologous biological bypass graft of limb | SNOMED CT |
| Peripheral vascular disease | 46271459 | 709584004 | Atherosclerosis of bypass graft of lower limb | SNOMED CT |
| Peripheral vascular disease | 40483538 | 442439008 | Atherosclerosis of bypass graft of limb | SNOMED CT |
| Peripheral vascular disease | 40484541 | 442693003 | Atherosclerosis of autologous vein bypass graft of limb | SNOMED CT |
| Peripheral vascular disease | 37110250 | 724440007 | Atherosclerosis of artery of lower limb | SNOMED CT |
| Peripheral vascular disease | 40479625 | 441574008 | Atherosclerosis of artery | SNOMED CT |
| Peripheral vascular disease | 315558 | 51274000 | Atherosclerosis of arteries of the extremities | SNOMED CT |
| Peripheral vascular disease | 312934 | 81817003 | Atherosclerosis of aorta | SNOMED CT |
| Peripheral vascular disease | 318443 | 72092001 | Arteriosclerotic vascular disease | SNOMED CT |
| Peripheral vascular disease | 317577 | 49176002 | Arteriosclerotic gangrene | SNOMED CT |
| Peripheral vascular disease | 317585 | 67362008 | Aortic aneurysm | SNOMED CT |
| Peripheral vascular disease | 443622 | 433068007 | Aneurysm of thoracic aorta | SNOMED CT |
| Peripheral vascular disease | 4332246 | 432119003 | Aneurysm | SNOMED CT |
| Peripheral vascular disease | 321314 | 75878002 | Abdominal aortic aneurysm without rupture | SNOMED CT |
| Peripheral vascular disease | 198177 | 233985008 | Abdominal aortic aneurysm | SNOMED CT |
| Congestive heart failure | 443580 | 417996009 | Systolic heart failure | SNOMED CT |
| Congestive heart failure | 4273632 | 367363000 | Right ventricular failure | SNOMED CT |
| Congestive heart failure | 4195785 | 44313006 | Right heart failure secondary to left heart failure | SNOMED CT |
| Congestive heart failure | 4190773 | 415295002 | Restrictive cardiomyopathy | SNOMED CT |
| Congestive heart failure | 320122 | 195023001 | Nutritional and metabolic cardiomyopathies | SNOMED CT |
| Congestive heart failure | 4172864 | 276514007 | Neonatal cardiac failure | SNOMED CT |
| Congestive heart failure | 316994 | 83105008 | Malignant hypertensive heart disease with congestive heart failure | SNOMED CT |
| Congestive heart failure | 439846 | 85232009 | Left heart failure | SNOMED CT |
| Congestive heart failure | 444101 | 46113002 | Hypertensive heart failure | SNOMED CT |
| Congestive heart failure | 314378 | 5148006 | Hypertensive heart disease with congestive heart failure | SNOMED CT |
| Congestive heart failure | 439694 | 194781004 | Hypertensive heart and renal disease with both (congestive) heart failure and renal failure | SNOMED CT |
| Congestive heart failure | 439696 | 194779001 | Hypertensive heart and renal disease with (congestive) heart failure | SNOMED CT |
| Congestive heart failure | 44782728 | 15781000119107 | Hypertensive heart AND chronic kidney disease with congestive heart failure | SNOMED CT |
| Congestive heart failure | 4004279 | 10091002 | High output heart failure | SNOMED CT |
| Congestive heart failure | 316139 | 84114007 | Heart failure | SNOMED CT |
| Congestive heart failure | 4110961 | 194849004 | Generalized ischemic myocardial dysfunction | SNOMED CT |
| Congestive heart failure | 318773 | 83521008 | Dilated cardiomyopathy secondary to alcohol | SNOMED CT |
| Congestive heart failure | 4163710 | 399020009 | Dilated cardiomyopathy | SNOMED CT |
| Congestive heart failure | 443587 | 418304008 | Diastolic heart failure | SNOMED CT |
| Congestive heart failure | 315295 | 82523003 | Congestive rheumatic heart failure | SNOMED CT |
| Congestive heart failure | 319835 | 42343007 | Congestive heart failure | SNOMED CT |
| Congestive heart failure | 40482727 | 442304009 | Combined systolic and diastolic dysfunction | SNOMED CT |
| Congestive heart failure | 40479192 | 441481004 | Chronic systolic heart failure | SNOMED CT |
| Congestive heart failure | 4014159 | 10335000 | Chronic right-sided heart failure | SNOMED CT |
| Congestive heart failure | 444031 | 48447003 | Chronic heart failure | SNOMED CT |
| Congestive heart failure | 40479576 | 441530006 | Chronic diastolic heart failure | SNOMED CT |
| Congestive heart failure | 4229440 | 88805009 | Chronic congestive heart failure | SNOMED CT |
| Congestive heart failure | 44782719 | 153941000119100 | Chronic combined systolic and diastolic heart failure | SNOMED CT |
| Congestive heart failure | 320746 | 195029002 | Cardiomyopathy associated with another disorder | SNOMED CT |
| Congestive heart failure | 321319 | 85898001 | Cardiomyopathy | SNOMED CT |
| Congestive heart failure | 4215802 | 71892000 | Cardiac asthma | SNOMED CT |
| Congestive heart failure | 4242669 | 92506005 | Biventricular congestive heart failure | SNOMED CT |
| Congestive heart failure | 439698 | 194767001 | Benign hypertensive heart disease with congestive cardiac failure | SNOMED CT |
| Congestive heart failure | 40480603 | 443254009 | Acute systolic heart failure | SNOMED CT |
| Congestive heart failure | 4233424 | 359617009 | Acute right-sided heart failure | SNOMED CT |
| Congestive heart failure | 40480602 | 443253003 | Acute on chronic systolic heart failure | SNOMED CT |
| Congestive heart failure | 37309625 | 16838951000119100 | Acute on chronic right-sided congestive heart failure | SNOMED CT |
| Congestive heart failure | 40481043 | 443344007 | Acute on chronic diastolic heart failure | SNOMED CT |
| Congestive heart failure | 44782733 | 153951000119103 | Acute on chronic combined systolic and diastolic heart failure | SNOMED CT |
| Congestive heart failure | 40481042 | 443343001 | Acute diastolic heart failure | SNOMED CT |
| Congestive heart failure | 44782718 | 153931000119109 | Acute combined systolic and diastolic heart failure | SNOMED CT |
| Malignancy, except skin neoplasms | 138708 | 91855006 | Acute leukemia | SNOMED CT |
| Malignancy, except skin neoplasms | 135496 | 91854005 | Acute leukemia in remission | SNOMED CT |
| Malignancy, except skin neoplasms | 134305 | 91857003 | Acute lymphoid leukemia | SNOMED CT |
| Malignancy, except skin neoplasms | 141816 | 91856007 | Acute lymphoid leukemia in remission | SNOMED CT |
| Malignancy, except skin neoplasms | 36712834 | 12301000132103 | Acute lymphoid leukemia relapse | SNOMED CT |
| Malignancy, except skin neoplasms | 4079686 | 277602003 | Acute megakaryoblastic leukemia | SNOMED CT |
| Malignancy, except skin neoplasms | 135768 | 413441006 | Acute monocytic leukemia | SNOMED CT |
| Malignancy, except skin neoplasms | 140672 | 91858008 | Acute monocytic leukemia in remission | SNOMED CT |
| Malignancy, except skin neoplasms | 4189938 | 413442004 | Acute monocytic/monoblastic leukemia | SNOMED CT |
| Malignancy, except skin neoplasms | 135762 | 91860005 | Acute myeloid leukemia in remission | SNOMED CT |
| Malignancy, except skin neoplasms | 40483761 | 445448008 | Acute myeloid leukemia with myelodysplasia-related changes | SNOMED CT |
| Malignancy, except skin neoplasms | 40481524 | 444911000 | Acute myeloid leukemia with t(9:11)(p22;q23); MLLT3-MLL | SNOMED CT |
| Malignancy, except skin neoplasms | 140352 | 91861009 | Acute myeloid leukemia, disease | SNOMED CT |
| Malignancy, except skin neoplasms | 4003184 | 109991003 | Acute panmyelosis with myelofibrosis | SNOMED CT |
| Malignancy, except skin neoplasms | 4002497 | 110004001 | Acute promyelocytic leukemia, FAB M3 | SNOMED CT |
| Malignancy, except skin neoplasms | 4137687 | 425869007 | Acute promyelocytic leukemia, FAB M3, in remission | SNOMED CT |
| Malignancy, except skin neoplasms | 4003188 | 110007008 | Adult T-cell leukemia/lymphoma | SNOMED CT |
| Malignancy, except skin neoplasms | 4297355 | 404136008 | Aggressive NK-cell leukemia involving skin | SNOMED CT |
| Malignancy, except skin neoplasms | 37396742 | 716655008 | Aggressive systemic mastocytosis | SNOMED CT |
| Malignancy, except skin neoplasms | 4003835 | 109982002 | Alpha heavy chain disease (clinical) | SNOMED CT |
| Malignancy, except skin neoplasms | 40488896 | 448212009 | Anaplastic large cell lymphoma, ALK negative | SNOMED CT |
| Malignancy, except skin neoplasms | 4299149 | 404134006 | Anaplastic large T-cell systemic malignant lymphoma | SNOMED CT |
| Malignancy, except skin neoplasms | 4003021 | 109844006 | Angiosarcoma of liver | SNOMED CT |
| Malignancy, except skin neoplasms | 4079282 | 277589003 | Atypical chronic myeloid leukemia | SNOMED CT |
| Malignancy, except skin neoplasms | 4173963 | 277571004 | B-cell acute lymphoblastic leukemia | SNOMED CT |
| Malignancy, except skin neoplasms | 4082311 | 277473004 | B-cell chronic lymphocytic leukemia | SNOMED CT |
| Malignancy, except skin neoplasms | 434592 | 109979007 | B-cell lymphoma (clinical) | SNOMED CT |
| Malignancy, except skin neoplasms | 4173974 | 277619001 | B-cell prolymphocytic leukemia | SNOMED CT |
| Malignancy, except skin neoplasms | 4041800 | 118617000 | Burkitt's lymphoma (clinical) | SNOMED CT |
| Malignancy, except skin neoplasms | 4097560 | 188512009 | Burkitt's lymphoma of intra-abdominal lymph nodes | SNOMED CT |
| Malignancy, except skin neoplasms | 193428 | 188515006 | Burkitt's lymphoma of intrapelvic lymph nodes | SNOMED CT |
| Malignancy, except skin neoplasms | 4091768 | 188511002 | Burkitt's lymphoma of intrathoracic lymph nodes | SNOMED CT |
| Malignancy, except skin neoplasms | 4097561 | 188513004 | Burkitt's lymphoma of lymph nodes of axilla and upper limb | SNOMED CT |
| Malignancy, except skin neoplasms | 196055 | 188514005 | Burkitt's lymphoma of lymph nodes of inguinal region and lower limb | SNOMED CT |
| Malignancy, except skin neoplasms | 315481 | 188517003 | Burkitt's lymphoma of lymph nodes of multiple sites | SNOMED CT |
| Malignancy, except skin neoplasms | 439293 | 188516007 | Burkitt's lymphoma of spleen | SNOMED CT |
| Malignancy, except skin neoplasms | 435758 | 92516002 | Burkitt's tumor of extranodal AND/OR solid organ site | SNOMED CT |
| Malignancy, except skin neoplasms | 433426 | 92511007 | Burkitt's tumor of lymph nodes of axilla AND/OR upper limb | SNOMED CT |
| Malignancy, except skin neoplasms | 437504 | 92512000 | Burkitt's tumor of lymph nodes of head, face AND/OR neck | SNOMED CT |
| Malignancy, except skin neoplasms | 140057 | 92812005 | Chronic leukemia | SNOMED CT |
| Malignancy, except skin neoplasms | 134597 | 92811003 | Chronic leukemia in remission | SNOMED CT |
| Malignancy, except skin neoplasms | 133438 | 92813000 | Chronic lymphoid leukemia in remission | SNOMED CT |
| Malignancy, except skin neoplasms | 138379 | 92814006 | Chronic lymphoid leukemia, disease | SNOMED CT |
| Malignancy, except skin neoplasms | 136056 | 188745007 | Chronic monocytic leukemia | SNOMED CT |
| Malignancy, except skin neoplasms | 760932 | 122881000119107 | Chronic monocytic leukemia in relapse | SNOMED CT |
| Malignancy, except skin neoplasms | 134603 | 92818009 | Chronic myeloid leukemia | SNOMED CT |
| Malignancy, except skin neoplasms | 132572 | 92817004 | Chronic myeloid leukemia in remission | SNOMED CT |
| Malignancy, except skin neoplasms | 4133599 | 127225006 | Chronic myelomonocytic leukemia | SNOMED CT |
| Malignancy, except skin neoplasms | 4082487 | 277613000 | Cutaneous/peripheral T-cell lymphoma | SNOMED CT |
| Malignancy, except skin neoplasms | 40486171 | 449220000 | Diffuse follicle center lymphoma | SNOMED CT |
| Malignancy, except skin neoplasms | 44808122 | 847741000000106 | Diffuse large B-cell lymphoma | SNOMED CT |
| Malignancy, except skin neoplasms | 4300704 | 404148006 | Diffuse large B-cell lymphoma (nodal/systemic with skin involvement) | SNOMED CT |
| Malignancy, except skin neoplasms | 4003830 | 109962001 | Diffuse non-Hodgkin's lymphoma | SNOMED CT |
| Malignancy, except skin neoplasms | 4003832 | 109966003 | Diffuse non-Hodgkin's lymphoma, immunoblastic (clinical) | SNOMED CT |
| Malignancy, except skin neoplasms | 4001328 | 109965004 | Diffuse non-Hodgkin's lymphoma, lymphoblastic (clinical) | SNOMED CT |
| Malignancy, except skin neoplasms | 4002356 | 109968002 | Diffuse non-Hodgkin's lymphoma, small cell (clinical) | SNOMED CT |
| Malignancy, except skin neoplasms | 4003831 | 109964000 | Diffuse non-Hodgkin's lymphoma, undifferentiated | SNOMED CT |
| Malignancy, except skin neoplasms | 138099 | 93451002 | Erythroleukemia, FAB M6 | SNOMED CT |
| Malignancy, except skin neoplasms | 4139358 | 426642002 | Erythroleukemia, FAB M6 in remission | SNOMED CT |
| Malignancy, except skin neoplasms | 4094548 | 188718006 | Extramedullary plasmacytoma | SNOMED CT |
| Malignancy, except skin neoplasms | 40482893 | 445269007 | Extranodal marginal zone B-cell lymphoma of mucosa-associated lymphoid tissue (MALT-lymphoma) | SNOMED CT |
| Malignancy, except skin neoplasms | 4212994 | 414166008 | Extranodal NK/T-cell lymphoma, nasal type | SNOMED CT |
| Malignancy, except skin neoplasms | 37395837 | 715414009 | Familial malignant neoplasm of pancreas | SNOMED CT |
| Malignancy, except skin neoplasms | 4147411 | 308121000 | Follicular non-Hodgkin's lymphoma | SNOMED CT |
| Malignancy, except skin neoplasms | 45765770 | 702786004 | Follicular non-Hodgkin's lymphoma diffuse follicle center sub-type grade 1 | SNOMED CT |
| Malignancy, except skin neoplasms | 4003833 | 109972003 | Follicular non-Hodgkin's lymphoma, large cell (clinical) | SNOMED CT |
| Malignancy, except skin neoplasms | 4001329 | 109971005 | Follicular non-Hodgkin's lymphoma, mixed small cleaved cell and large cell (clinical) | SNOMED CT |
| Malignancy, except skin neoplasms | 4002357 | 109970006 | Follicular non-Hodgkin's lymphoma, small cleaved cell (clinical) | SNOMED CT |
| Malignancy, except skin neoplasms | 4170421 | 420120006 | Gastrointestinal stromal tumor | SNOMED CT |
| Malignancy, except skin neoplasms | 40486465 | 446124001 | Gastrointestinal stromal tumor of esophagus | SNOMED CT |
| Malignancy, except skin neoplasms | 40490328 | 446925001 | Gastrointestinal stromal tumor of large intestine | SNOMED CT |
| Malignancy, except skin neoplasms | 40492267 | 445737002 | Gastrointestinal stromal tumor of small intestine | SNOMED CT |
| Malignancy, except skin neoplasms | 40492266 | 445736006 | Gastrointestinal stromal tumor of stomach | SNOMED CT |
| Malignancy, except skin neoplasms | 4038845 | 118613001 | Hairy cell leukemia (clinical) | SNOMED CT |
| Malignancy, except skin neoplasms | 4245460 | 93151007 | Hairy cell leukemia of spleen | SNOMED CT |
| Malignancy, except skin neoplasms | 4217892 | 39795003 | Hand-Schüller-Christian disease | SNOMED CT |
| Malignancy, except skin neoplasms | 4288751 | 68979007 | Heavy chain disease | SNOMED CT |
| Malignancy, except skin neoplasms | 4001172 | 109843000 | Hepatoblastoma | SNOMED CT |
| Malignancy, except skin neoplasms | 37396884 | 716859000 | Hereditary diffuse carcinoma of stomach | SNOMED CT |
| Malignancy, except skin neoplasms | 4002494 | 109988003 | Histiocytic sarcoma (clinical) | SNOMED CT |
| Malignancy, except skin neoplasms | 4038839 | 118605002 | Hodgkin lymphoma, nodular lymphocyte predominance (clinical) | SNOMED CT |
| Malignancy, except skin neoplasms | 4038835 | 118599009 | Hodgkin's disease (clinical) | SNOMED CT |
| Malignancy, except skin neoplasms | 436651 | 93528000 | Hodgkin's disease of extranodal AND/OR solid organ site | SNOMED CT |
| Malignancy, except skin neoplasms | 200355 | 93520007 | Hodgkin's disease of intra-abdominal lymph nodes | SNOMED CT |
| Malignancy, except skin neoplasms | 195761 | 93521006 | Hodgkin's disease of intrapelvic lymph nodes | SNOMED CT |
| Malignancy, except skin neoplasms | 440965 | 93522004 | Hodgkin's disease of intrathoracic lymph nodes | SNOMED CT |
| Malignancy, except skin neoplasms | 434877 | 93523009 | Hodgkin's disease of lymph nodes of axilla AND/OR upper limb | SNOMED CT |
| Malignancy, except skin neoplasms | 437818 | 93524003 | Hodgkin's disease of lymph nodes of head, face AND/OR neck | SNOMED CT |
| Malignancy, except skin neoplasms | 436652 | 93525002 | Hodgkin's disease of lymph nodes of inguinal region AND/OR lower limb | SNOMED CT |
| Malignancy, except skin neoplasms | 316356 | 93526001 | Hodgkin's disease of lymph nodes of multiple sites | SNOMED CT |
| Malignancy, except skin neoplasms | 198704 | 93527005 | Hodgkin's disease of spleen | SNOMED CT |
| Malignancy, except skin neoplasms | 4041798 | 118610003 | Hodgkin's disease, lymphocytic depletion (clinical) | SNOMED CT |
| Malignancy, except skin neoplasms | 432267 | 93492006 | Hodgkin's disease, lymphocytic depletion of extranodal AND/OR solid organ site | SNOMED CT |
| Malignancy, except skin neoplasms | 439285 | 188587006 | Hodgkin's disease, lymphocytic depletion of intra-abdominal lymph nodes | SNOMED CT |
| Malignancy, except skin neoplasms | 439282 | 188591001 | Hodgkin's disease, lymphocytic depletion of intrapelvic lymph nodes | SNOMED CT |
| Malignancy, except skin neoplasms | 141243 | 188586002 | Hodgkin's disease, lymphocytic depletion of intrathoracic lymph nodes | SNOMED CT |
| Malignancy, except skin neoplasms | 442151 | 93487009 | Hodgkin's disease, lymphocytic depletion of lymph nodes of axilla AND/OR upper limb | SNOMED CT |
| Malignancy, except skin neoplasms | 433161 | 93488004 | Hodgkin's disease, lymphocytic depletion of lymph nodes of head, face AND/OR neck | SNOMED CT |
| Malignancy, except skin neoplasms | 442150 | 93489007 | Hodgkin's disease, lymphocytic depletion of lymph nodes of inguinal region AND/OR lower limb | SNOMED CT |
| Malignancy, except skin neoplasms | 313980 | 188593003 | Hodgkin's disease, lymphocytic depletion of lymph nodes of multiple sites | SNOMED CT |
| Malignancy, except skin neoplasms | 439281 | 188592008 | Hodgkin's disease, lymphocytic depletion of spleen | SNOMED CT |
| Malignancy, except skin neoplasms | 438373 | 93501005 | Hodgkin's disease, lymphocytic-histiocytic predominance of extranodal AND/OR solid organ site | SNOMED CT |
| Malignancy, except skin neoplasms | 201242 | 93493001 | Hodgkin's disease, lymphocytic-histiocytic predominance of intra-abdominal lymph nodes | SNOMED CT |
| Malignancy, except skin neoplasms | 194876 | 93494007 | Hodgkin's disease, lymphocytic-histiocytic predominance of intrapelvic lymph nodes | SNOMED CT |
| Malignancy, except skin neoplasms | 440351 | 93495008 | Hodgkin's disease, lymphocytic-histiocytic predominance of intrathoracic lymph nodes | SNOMED CT |
| Malignancy, except skin neoplasms | 438703 | 93496009 | Hodgkin's disease, lymphocytic-histiocytic predominance of lymph nodes of axilla AND/OR upper limb | SNOMED CT |
| Malignancy, except skin neoplasms | 442163 | 93497000 | Hodgkin's disease, lymphocytic-histiocytic predominance of lymph nodes of head, face AND/OR neck | SNOMED CT |
| Malignancy, except skin neoplasms | 442162 | 93498005 | Hodgkin's disease, lymphocytic-histiocytic predominance of lymph nodes of inguinal region AND/OR lower limb | SNOMED CT |
| Malignancy, except skin neoplasms | 321522 | 188562004 | Hodgkin's disease, lymphocytic-histiocytic predominance of lymph nodes of multiple sites | SNOMED CT |
| Malignancy, except skin neoplasms | 198381 | 93500006 | Hodgkin's disease, lymphocytic-histiocytic predominance of spleen | SNOMED CT |
| Malignancy, except skin neoplasms | 4038843 | 118609008 | Hodgkin's disease, mixed cellularity (clinical) | SNOMED CT |
| Malignancy, except skin neoplasms | 435203 | 93510002 | Hodgkin's disease, mixed cellularity of extranodal AND/OR solid organ site | SNOMED CT |
| Malignancy, except skin neoplasms | 192268 | 188577007 | Hodgkin's disease, mixed cellularity of intra-abdominal lymph nodes | SNOMED CT |
| Malignancy, except skin neoplasms | 194594 | 188580008 | Hodgkin's disease, mixed cellularity of intrapelvic lymph nodes | SNOMED CT |
| Malignancy, except skin neoplasms | 136655 | 188576003 | Hodgkin's disease, mixed cellularity of intrathoracic lymph nodes | SNOMED CT |
| Malignancy, except skin neoplasms | 434299 | 93505001 | Hodgkin's disease, mixed cellularity of lymph nodes of axilla AND/OR upper limb | SNOMED CT |
| Malignancy, except skin neoplasms | 442161 | 93506000 | Hodgkin's disease, mixed cellularity of lymph nodes of head, face AND/OR neck | SNOMED CT |
| Malignancy, except skin neoplasms | 442160 | 93507009 | Hodgkin's disease, mixed cellularity of lymph nodes of inguinal region AND/OR lower limb | SNOMED CT |
| Malignancy, except skin neoplasms | 315202 | 188582000 | Hodgkin's disease, mixed cellularity of lymph nodes of multiple sites | SNOMED CT |
| Malignancy, except skin neoplasms | 442159 | 93509007 | Hodgkin's disease, mixed cellularity of spleen | SNOMED CT |
| Malignancy, except skin neoplasms | 4038842 | 118608000 | Hodgkin's disease, nodular sclerosis (clinical) | SNOMED CT |
| Malignancy, except skin neoplasms | 434302 | 93519001 | Hodgkin's disease, nodular sclerosis of extranodal AND/OR solid organ site | SNOMED CT |
| Malignancy, except skin neoplasms | 193434 | 188567005 | Hodgkin's disease, nodular sclerosis of intra-abdominal lymph nodes | SNOMED CT |
| Malignancy, except skin neoplasms | 199760 | 188570009 | Hodgkin's disease, nodular sclerosis of intrapelvic lymph nodes | SNOMED CT |
| Malignancy, except skin neoplasms | 136928 | 188566001 | Hodgkin's disease, nodular sclerosis of intrathoracic lymph nodes | SNOMED CT |
| Malignancy, except skin neoplasms | 314600 | 93514006 | Hodgkin's disease, nodular sclerosis of lymph nodes of axilla AND/OR upper limb | SNOMED CT |
| Malignancy, except skin neoplasms | 442158 | 93515007 | Hodgkin's disease, nodular sclerosis of lymph nodes of head, face AND/OR neck | SNOMED CT |
| Malignancy, except skin neoplasms | 442157 | 93516008 | Hodgkin's disease, nodular sclerosis of lymph nodes of inguinal region AND/OR lower limb | SNOMED CT |
| Malignancy, except skin neoplasms | 315763 | 188572001 | Hodgkin's disease, nodular sclerosis of lymph nodes of multiple sites | SNOMED CT |
| Malignancy, except skin neoplasms | 193157 | 93518009 | Hodgkin's disease, nodular sclerosis of spleen | SNOMED CT |
| Malignancy, except skin neoplasms | 4041797 | 118602004 | Hodgkin's granuloma (clinical) | SNOMED CT |
| Malignancy, except skin neoplasms | 439291 | 188536008 | Hodgkin's granuloma of intra-abdominal lymph nodes | SNOMED CT |
| Malignancy, except skin neoplasms | 442156 | 93530003 | Hodgkin's granuloma of intrapelvic lymph nodes | SNOMED CT |
| Malignancy, except skin neoplasms | 440354 | 93531004 | Hodgkin's granuloma of intrathoracic lymph nodes | SNOMED CT |
| Malignancy, except skin neoplasms | 442155 | 93532006 | Hodgkin's granuloma of lymph nodes of axilla AND/OR upper limb | SNOMED CT |
| Malignancy, except skin neoplasms | 442154 | 93533001 | Hodgkin's granuloma of lymph nodes of head, face AND/OR neck | SNOMED CT |
| Malignancy, except skin neoplasms | 442153 | 93534007 | Hodgkin's granuloma of lymph nodes of inguinal region AND/OR lower limb | SNOMED CT |
| Malignancy, except skin neoplasms | 320337 | 188541000 | Hodgkin's granuloma of lymph nodes of multiple sites | SNOMED CT |
| Malignancy, except skin neoplasms | 196355 | 93536009 | Hodgkin's granuloma of spleen | SNOMED CT |
| Malignancy, except skin neoplasms | 198986 | 188529007 | Hodgkin's paragranuloma of intrapelvic lymph nodes | SNOMED CT |
| Malignancy, except skin neoplasms | 438709 | 188524002 | Hodgkin's paragranuloma of intrathoracic lymph nodes | SNOMED CT |
| Malignancy, except skin neoplasms | 442152 | 93541001 | Hodgkin's paragranuloma of lymph nodes of axilla AND/OR upper limb | SNOMED CT |
| Malignancy, except skin neoplasms | 435494 | 93542008 | Hodgkin's paragranuloma of lymph nodes of head, face AND/OR neck | SNOMED CT |
| Malignancy, except skin neoplasms | 442144 | 93543003 | Hodgkin's paragranuloma of lymph nodes of inguinal region AND/OR lower limb | SNOMED CT |
| Malignancy, except skin neoplasms | 140663 | 188531003 | Hodgkin's paragranuloma of lymph nodes of multiple sites | SNOMED CT |
| Malignancy, except skin neoplasms | 4038840 | 118606001 | Hodgkin's sarcoma (clinical) | SNOMED CT |
| Malignancy, except skin neoplasms | 198372 | 93547002 | Hodgkin's sarcoma of intra-abdominal lymph nodes | SNOMED CT |
| Malignancy, except skin neoplasms | 195196 | 93548007 | Hodgkin's sarcoma of intrapelvic lymph nodes | SNOMED CT |
| Malignancy, except skin neoplasms | 433156 | 93549004 | Hodgkin's sarcoma of intrathoracic lymph nodes | SNOMED CT |
| Malignancy, except skin neoplasms | 320049 | 93550004 | Hodgkin's sarcoma of lymph nodes of axilla AND/OR upper limb | SNOMED CT |
| Malignancy, except skin neoplasms | 442146 | 93551000 | Hodgkin's sarcoma of lymph nodes of head, face AND/OR neck | SNOMED CT |
| Malignancy, except skin neoplasms | 442145 | 93552007 | Hodgkin's sarcoma of lymph nodes of inguinal region AND/OR lower limb | SNOMED CT |
| Malignancy, except skin neoplasms | 316649 | 188551004 | Hodgkin's sarcoma of lymph nodes of multiple sites | SNOMED CT |
| Malignancy, except skin neoplasms | 198710 | 93554008 | Hodgkin's sarcoma of spleen | SNOMED CT |
| Malignancy, except skin neoplasms | 441523 | 275524009 | Immunoproliferative neoplasm | SNOMED CT |
| Malignancy, except skin neoplasms | 4142878 | 427374007 | Immunoproliferative neoplasm in remission | SNOMED CT |
| Malignancy, except skin neoplasms | 4003836 | 109985000 | Immunoproliferative small intestinal disease (clinical) | SNOMED CT |
| Malignancy, except skin neoplasms | 4001664 | 109842005 | Intrahepatic bile duct carcinoma | SNOMED CT |
| Malignancy, except skin neoplasms | 40482847 | 445227008 | Juvenile myelomonocytic leukemia | SNOMED CT |
| Malignancy, except skin neoplasms | 434584 | 109385007 | Kaposi's sarcoma (clinical) | SNOMED CT |
| Malignancy, except skin neoplasms | 198085 | 109389001 | Kaposi's sarcoma of gastrointestinal tract | SNOMED CT |
| Malignancy, except skin neoplasms | 254583 | 109390005 | Kaposi's sarcoma of lung | SNOMED CT |
| Malignancy, except skin neoplasms | 312846 | 109391009 | Kaposi's sarcoma of lymph nodes | SNOMED CT |
| Malignancy, except skin neoplasms | 4033893 | 109392002 | Kaposi's sarcoma of multiple organs | SNOMED CT |
| Malignancy, except skin neoplasms | 140958 | 109388009 | Kaposi's sarcoma of palate | SNOMED CT |
| Malignancy, except skin neoplasms | 258981 | 109386008 | Kaposi's sarcoma of skin | SNOMED CT |
| Malignancy, except skin neoplasms | 375479 | 188029000 | Kaposi's sarcoma of soft tissue | SNOMED CT |
| Malignancy, except skin neoplasms | 4038846 | 118614007 | Langerhans cell histiocytosis, disseminated (clinical) | SNOMED CT |
| Malignancy, except skin neoplasms | 4044708 | 129000002 | Langerhans cell histiocytosis, unifocal (clinical) | SNOMED CT |
| Malignancy, except skin neoplasms | 441235 | 277637000 | Large cell anaplastic lymphoma | SNOMED CT |
| Malignancy, except skin neoplasms | 40481357 | 441962003 | Large cell lymphoma of intrapelvic lymph nodes | SNOMED CT |
| Malignancy, except skin neoplasms | 439761 | 93141006 | Letterer-Siwe disease of extranodal AND/OR solid organ site | SNOMED CT |
| Malignancy, except skin neoplasms | 444467 | 93133006 | Letterer-Siwe disease of intra-abdominal lymph nodes | SNOMED CT |
| Malignancy, except skin neoplasms | 196363 | 93134000 | Letterer-Siwe disease of intrapelvic lymph nodes | SNOMED CT |
| Malignancy, except skin neoplasms | 434595 | 93135004 | Letterer-Siwe disease of intrathoracic lymph nodes | SNOMED CT |
| Malignancy, except skin neoplasms | 444466 | 93136003 | Letterer-Siwe disease of lymph nodes of axilla AND/OR upper limb | SNOMED CT |
| Malignancy, except skin neoplasms | 436362 | 93137007 | Letterer-Siwe disease of lymph nodes of head, face AND/OR neck | SNOMED CT |
| Malignancy, except skin neoplasms | 444465 | 93138002 | Letterer-Siwe disease of lymph nodes of inguinal region AND/OR lower limb | SNOMED CT |
| Malignancy, except skin neoplasms | 141814 | 93139005 | Letterer-Siwe disease of lymph nodes of multiple sites | SNOMED CT |
| Malignancy, except skin neoplasms | 197816 | 93140007 | Letterer-Siwe disease of spleen | SNOMED CT |
| Malignancy, except skin neoplasms | 317510 | 93143009 | Leukemia | SNOMED CT |
| Malignancy, except skin neoplasms | 135766 | 93142004 | Leukemia in remission | SNOMED CT |
| Malignancy, except skin neoplasms | 132852 | 93152000 | Leukemic reticuloendotheliosis of extranodal AND/OR solid organ site | SNOMED CT |
| Malignancy, except skin neoplasms | 193429 | 93144003 | Leukemic reticuloendotheliosis of intra-abdominal lymph nodes | SNOMED CT |
| Malignancy, except skin neoplasms | 196650 | 93145002 | Leukemic reticuloendotheliosis of intrapelvic lymph nodes | SNOMED CT |
| Malignancy, except skin neoplasms | 442095 | 93146001 | Leukemic reticuloendotheliosis of intrathoracic lymph nodes | SNOMED CT |
| Malignancy, except skin neoplasms | 439269 | 188648000 | Leukemic reticuloendotheliosis of lymph nodes of axilla and upper limb | SNOMED CT |
| Malignancy, except skin neoplasms | 132570 | 188645002 | Leukemic reticuloendotheliosis of lymph nodes of head, face and neck | SNOMED CT |
| Malignancy, except skin neoplasms | 439268 | 188649008 | Leukemic reticuloendotheliosis of lymph nodes of inguinal region and lower limb | SNOMED CT |
| Malignancy, except skin neoplasms | 318989 | 93150008 | Leukemic reticuloendotheliosis of lymph nodes of multiple sites | SNOMED CT |
| Malignancy, except skin neoplasms | 4001171 | 109841003 | Liver cell carcinoma | SNOMED CT |
| Malignancy, except skin neoplasms | 4038841 | 118607005 | Lymphocyte-rich classical Hodgkin lymphoma | SNOMED CT |
| Malignancy, except skin neoplasms | 4002358 | 109976000 | Lymphoepithelioid lymphoma (clinical) | SNOMED CT |
| Malignancy, except skin neoplasms | 132853 | 188725004 | Lymphoid leukemia | SNOMED CT |
| Malignancy, except skin neoplasms | 760934 | 122951000119108 | Lymphoid leukemia in relapse | SNOMED CT |
| Malignancy, except skin neoplasms | 134596 | 93169003 | Lymphoid leukemia in remission | SNOMED CT |
| Malignancy, except skin neoplasms | 200662 | 188498009 | Lymphosarcoma | SNOMED CT |
| Malignancy, except skin neoplasms | 440957 | 188487008 | Lymphosarcoma and reticulosarcoma | SNOMED CT |
| Malignancy, except skin neoplasms | 197228 | 188502002 | Lymphosarcoma of intra-abdominal lymph nodes | SNOMED CT |
| Malignancy, except skin neoplasms | 201810 | 188505000 | Lymphosarcoma of intrapelvic lymph nodes | SNOMED CT |
| Malignancy, except skin neoplasms | 135194 | 188501009 | Lymphosarcoma of intrathoracic lymph nodes | SNOMED CT |
| Malignancy, except skin neoplasms | 441803 | 188503007 | Lymphosarcoma of lymph nodes of axilla and upper limb | SNOMED CT |
| Malignancy, except skin neoplasms | 438380 | 188500005 | Lymphosarcoma of lymph nodes of head, face and neck | SNOMED CT |
| Malignancy, except skin neoplasms | 198702 | 188504001 | Lymphosarcoma of lymph nodes of inguinal region and lower limb | SNOMED CT |
| Malignancy, except skin neoplasms | 318697 | 188507008 | Lymphosarcoma of lymph nodes of multiple sites | SNOMED CT |
| Malignancy, except skin neoplasms | 195201 | 188506004 | Lymphosarcoma of spleen | SNOMED CT |
| Malignancy, except skin neoplasms | 4041104 | 118612006 | Malignant histiocytosis (clinical) | SNOMED CT |
| Malignancy, except skin neoplasms | 436929 | 93190006 | Malignant histiocytosis of extranodal AND/OR solid organ site | SNOMED CT |
| Malignancy, except skin neoplasms | 198098 | 93182006 | Malignant histiocytosis of intra-abdominal lymph nodes | SNOMED CT |
| Malignancy, except skin neoplasms | 192270 | 93183001 | Malignant histiocytosis of intrapelvic lymph nodes | SNOMED CT |
| Malignancy, except skin neoplasms | 441812 | 93184007 | Malignant histiocytosis of intrathoracic lymph nodes | SNOMED CT |
| Malignancy, except skin neoplasms | 435200 | 93185008 | Malignant histiocytosis of lymph nodes of axilla AND/OR upper limb | SNOMED CT |
| Malignancy, except skin neoplasms | 441525 | 93186009 | Malignant histiocytosis of lymph nodes of head, face AND/OR neck | SNOMED CT |
| Malignancy, except skin neoplasms | 197232 | 93187000 | Malignant histiocytosis of lymph nodes of inguinal region AND/OR lower limb | SNOMED CT |
| Malignancy, except skin neoplasms | 321815 | 93188005 | Malignant histiocytosis of lymph nodes of multiple sites | SNOMED CT |
| Malignancy, except skin neoplasms | 193721 | 93189002 | Malignant histiocytosis of spleen | SNOMED CT |
| Malignancy, except skin neoplasms | 4003834 | 109980005 | Malignant immunoproliferative disease (clinical) | SNOMED CT |
| Malignancy, except skin neoplasms | 432571 | 118600007 | Malignant lymphoma | SNOMED CT |
| Malignancy, except skin neoplasms | 4094544 | 188676008 | Malignant lymphoma - mixed small and large cell | SNOMED CT |
| Malignancy, except skin neoplasms | 440058 | 93199007 | Malignant lymphoma of extranodal AND/OR solid organ site | SNOMED CT |
| Malignancy, except skin neoplasms | 200349 | 93191005 | Malignant lymphoma of intra-abdominal lymph nodes | SNOMED CT |
| Malignancy, except skin neoplasms | 195195 | 93192003 | Malignant lymphoma of intrapelvic lymph nodes | SNOMED CT |
| Malignancy, except skin neoplasms | 435753 | 93193008 | Malignant lymphoma of intrathoracic lymph nodes | SNOMED CT |
| Malignancy, except skin neoplasms | 441521 | 93194002 | Malignant lymphoma of lymph nodes of axilla AND/OR upper limb | SNOMED CT |
| Malignancy, except skin neoplasms | 438698 | 93195001 | Malignant lymphoma of lymph nodes of head, face AND/OR neck | SNOMED CT |
| Malignancy, except skin neoplasms | 192560 | 93196000 | Malignant lymphoma of lymph nodes of inguinal region AND/OR lower limb | SNOMED CT |
| Malignancy, except skin neoplasms | 132841 | 93197009 | Malignant lymphoma of lymph nodes of multiple sites | SNOMED CT |
| Malignancy, except skin neoplasms | 200343 | 93198004 | Malignant lymphoma of spleen | SNOMED CT |
| Malignancy, except skin neoplasms | 4040379 | 118615008 | Malignant mast cell tumor (clinical) | SNOMED CT |
| Malignancy, except skin neoplasms | 4245916 | 93200005 | Malignant mast cell tumor of intra-abdominal lymph nodes | SNOMED CT |
| Malignancy, except skin neoplasms | 200667 | 93201009 | Malignant mast cell tumor of intrapelvic lymph nodes | SNOMED CT |
| Malignancy, except skin neoplasms | 434601 | 93202002 | Malignant mast cell tumor of intrathoracic lymph nodes | SNOMED CT |
| Malignancy, except skin neoplasms | 441245 | 93203007 | Malignant mast cell tumor of lymph nodes of axilla AND/OR upper limb | SNOMED CT |
| Malignancy, except skin neoplasms | 435495 | 93204001 | Malignant mast cell tumor of lymph nodes of head, face AND/OR neck | SNOMED CT |
| Malignancy, except skin neoplasms | 198705 | 93205000 | Malignant mast cell tumor of lymph nodes of inguinal region AND/OR lower limb | SNOMED CT |
| Malignancy, except skin neoplasms | 4095589 | 188032002 | Malignant melanoma of ear and/or external auditory canal | SNOMED CT |
| Malignancy, except skin neoplasms | 4089860 | 188030005 | Malignant melanoma of lip | SNOMED CT |
| Malignancy, except skin neoplasms | 4151263 | 269581007 | Malignant melanoma of lower limb | SNOMED CT |
| Malignancy, except skin neoplasms | 4095592 | 188044004 | Malignant melanoma of scalp and/or neck | SNOMED CT |
| Malignancy, except skin neoplasms | 141232 | 93655004 | Malignant melanoma of skin | SNOMED CT |
| Malignancy, except skin neoplasms | 4244051 | 93215006 | Malignant melanoma of skin of breast | SNOMED CT |
| Malignancy, except skin neoplasms | 434590 | 93224002 | Malignant melanoma of skin of eyelid | SNOMED CT |
| Malignancy, except skin neoplasms | 133713 | 93225001 | Malignant melanoma of skin of face | SNOMED CT |
| Malignancy, except skin neoplasms | 140048 | 93640008 | Malignant melanoma of skin of lip | SNOMED CT |
| Malignancy, except skin neoplasms | 139757 | 93641007 | Malignant melanoma of skin of lower limb | SNOMED CT |
| Malignancy, except skin neoplasms | 4244488 | 93643005 | Malignant melanoma of skin of nose | SNOMED CT |
| Malignancy, except skin neoplasms | 133714 | 93651008 | Malignant melanoma of skin of trunk | SNOMED CT |
| Malignancy, except skin neoplasms | 438983 | 93653006 | Malignant melanoma of skin of upper limb | SNOMED CT |
| Malignancy, except skin neoplasms | 4153890 | 269579005 | Malignant melanoma of trunk | SNOMED CT |
| Malignancy, except skin neoplasms | 4149851 | 269580008 | Malignant melanoma of upper limb | SNOMED CT |
| Malignancy, except skin neoplasms | 4032870 | 109383000 | Malignant mesothelioma of pericardium | SNOMED CT |
| Malignancy, except skin neoplasms | 4111917 | 254645002 | Malignant mesothelioma of pleura | SNOMED CT |
| Malignancy, except skin neoplasms | 197506 | 188366002 | Malignant neoplasm of abdomen | SNOMED CT |
| Malignancy, except skin neoplasms | 40481902 | 443488001 | Malignant neoplasm of anorectum | SNOMED CT |
| Malignancy, except skin neoplasms | 77812 | 188242006 | Malignant neoplasm of anterior wall of urinary bladder | SNOMED CT |
| Malignancy, except skin neoplasms | 377229 | 187833006 | Malignant neoplasm of auditory tube, middle ear and mastoid air cells | SNOMED CT |
| Malignancy, except skin neoplasms | 4091467 | 188156001 | Malignant neoplasm of axillary tail of female breast | SNOMED CT |
| Malignancy, except skin neoplasms | 258084 | 187900002 | Malignant neoplasm of bones of skull and face | SNOMED CT |
| Malignancy, except skin neoplasms | 4091490 | 188280007 | Malignant neoplasm of cerebrum (excluding lobes and ventricles) | SNOMED CT |
| Malignancy, except skin neoplasms | 4095018 | 188015001 | Malignant neoplasm of connective and soft tissue of abdomen | SNOMED CT |
| Malignancy, except skin neoplasms | 4089777 | 187999008 | Malignant neoplasm of connective and soft tissue of hip and lower limb | SNOMED CT |
| Malignancy, except skin neoplasms | 201231 | 188019007 | Malignant neoplasm of connective and soft tissue of pelvis | SNOMED CT |
| Malignancy, except skin neoplasms | 4092358 | 188009001 | Malignant neoplasm of connective and soft tissue of thorax | SNOMED CT |
| Malignancy, except skin neoplasms | 4092235 | 187991006 | Malignant neoplasm of connective and soft tissue of upper limb and shoulder | SNOMED CT |
| Malignancy, except skin neoplasms | 194286 | 188189001 | Malignant neoplasm of corpus uteri, excluding isthmus | SNOMED CT |
| Malignancy, except skin neoplasms | 40491001 | 448675008 | Malignant neoplasm of digestive system | SNOMED CT |
| Malignancy, except skin neoplasms | 374297 | 188261005 | Malignant neoplasm of eyeball excluding conjunctiva, cornea, retina and choroid | SNOMED CT |
| Malignancy, except skin neoplasms | 4095748 | 188191009 | Malignant neoplasm of fundus of corpus uteri | SNOMED CT |
| Malignancy, except skin neoplasms | 73153 | 188241004 | Malignant neoplasm of lateral wall of urinary bladder | SNOMED CT |
| Malignancy, except skin neoplasms | 436045 | 271323007 | Malignant neoplasm of lip, oral cavity and pharynx | SNOMED CT |
| Malignancy, except skin neoplasms | 4246127 | 93870000 | Malignant neoplasm of liver | SNOMED CT |
| Malignancy, except skin neoplasms | 4095432 | 187767006 | Malignant neoplasm of liver and intrahepatic bile ducts | SNOMED CT |
| Malignancy, except skin neoplasms | 4334322 | 430621000 | Malignant neoplasm of lower respiratory tract | SNOMED CT |
| Malignancy, except skin neoplasms | 4095442 | 187828007 | Malignant neoplasm of nasal cavities, middle ear and accessory sinuses | SNOMED CT |
| Malignancy, except skin neoplasms | 4091464 | 188147009 | Malignant neoplasm of nipple and areola of female breast | SNOMED CT |
| Malignancy, except skin neoplasms | 4091469 | 188163001 | Malignant neoplasm of nipple and areola of male breast | SNOMED CT |
| Malignancy, except skin neoplasms | 4092223 | 187906008 | Malignant neoplasm of orbital bone | SNOMED CT |
| Malignancy, except skin neoplasms | 4091486 | 188256008 | Malignant neoplasm of overlapping lesion of urinary organs | SNOMED CT |
| Malignancy, except skin neoplasms | 4089769 | 187952001 | Malignant neoplasm of pelvic bones, sacrum and coccyx | SNOMED CT |
| Malignancy, except skin neoplasms | 4091621 | 188326001 | Malignant neoplasm of peripheral nerve of abdomen | SNOMED CT |
| Malignancy, except skin neoplasms | 4097284 | 188327005 | Malignant neoplasm of peripheral nerve of pelvis | SNOMED CT |
| Malignancy, except skin neoplasms | 4094262 | 188325002 | Malignant neoplasm of peripheral nerve of thorax | SNOMED CT |
| Malignancy, except skin neoplasms | 4094260 | 188321006 | Malignant neoplasm of peripheral nerves and autonomic nervous system | SNOMED CT |
| Malignancy, except skin neoplasms | 4095892 | 188322004 | Malignant neoplasm of peripheral nerves of head, face and neck | SNOMED CT |
| Malignancy, except skin neoplasms | 4097283 | 188324003 | Malignant neoplasm of peripheral nerves of lower limb, including hip | SNOMED CT |
| Malignancy, except skin neoplasms | 4095304 | 188339002 | Malignant neoplasm of pituitary gland and craniopharyngeal duct | SNOMED CT |
| Malignancy, except skin neoplasms | 75756 | 188243001 | Malignant neoplasm of posterior wall of urinary bladder | SNOMED CT |
| Malignancy, except skin neoplasms | 4095430 | 187760008 | Malignant neoplasm of rectum, rectosigmoid junction and anus | SNOMED CT |
| Malignancy, except skin neoplasms | 40482784 | 443679004 | Malignant neoplasm of skeletal system | SNOMED CT |
| Malignancy, except skin neoplasms | 80665 | 188361007 | Malignant neoplasm of thorax | SNOMED CT |
| Malignancy, except skin neoplasms | 440655 | 187637005 | Malignant neoplasm of tongue, tip and lateral border | SNOMED CT |
| Malignancy, except skin neoplasms | 4095168 | 188247000 | Malignant neoplasm, overlapping lesion of bladder | SNOMED CT |
| Malignancy, except skin neoplasms | 4092513 | 188157005 | Malignant neoplasm, overlapping lesion of breast | SNOMED CT |
| Malignancy, except skin neoplasms | 4092515 | 188180002 | Malignant neoplasm, overlapping lesion of cervix uteri | SNOMED CT |
| Malignancy, except skin neoplasms | 4094409 | 188478004 | Malignant neoplasms of independent (primary) multiple sites | SNOMED CT |
| Malignancy, except skin neoplasms | 4110889 | 254980001 | Malignant tumor of acoustic vestibular nerve | SNOMED CT |
| Malignancy, except skin neoplasms | 4180790 | 363406005 | Malignant tumor of colon | SNOMED CT |
| Malignancy, except skin neoplasms | 4116238 | 255077007 | Malignant tumor of digestive organ | SNOMED CT |
| Malignancy, except skin neoplasms | 4112734 | 254611009 | Malignant tumor of endocrine pancreas | SNOMED CT |
| Malignancy, except skin neoplasms | 4114222 | 255056009 | Malignant tumor of head and neck | SNOMED CT |
| Malignancy, except skin neoplasms | 443389 | 363357005 | Malignant tumor of ill-defined site | SNOMED CT |
| Malignancy, except skin neoplasms | 4089530 | 187653008 | Malignant tumor of lateral floor of mouth | SNOMED CT |
| Malignancy, except skin neoplasms | 4177242 | 363504005 | Malignant tumor of lower limb | SNOMED CT |
| Malignancy, except skin neoplasms | 4147164 | 269475001 | Malignant tumor of lymphoid hemopoietic and related tissue | SNOMED CT |
| Malignancy, except skin neoplasms | 4094863 | 187752007 | Malignant tumor of Meckel's diverticulum | SNOMED CT |
| Malignancy, except skin neoplasms | 4095312 | 187692001 | Malignant tumor of nasopharynx | SNOMED CT |
| Malignancy, except skin neoplasms | 4114198 | 254969001 | Malignant tumor of olfactory tract | SNOMED CT |
| Malignancy, except skin neoplasms | 25189 | 363505006 | Malignant tumor of oral cavity | SNOMED CT |
| Malignancy, except skin neoplasms | 4180793 | 363418001 | Malignant tumor of pancreas | SNOMED CT |
| Malignancy, except skin neoplasms | 4177236 | 363484005 | Malignant tumor of pelvis | SNOMED CT |
| Malignancy, except skin neoplasms | 4089665 | 187801002 | Malignant tumor of peritoneum and retroperitoneum | SNOMED CT |
| Malignancy, except skin neoplasms | 4116235 | 255072001 | Malignant tumor of salivary gland | SNOMED CT |
| Malignancy, except skin neoplasms | 443397 | 363509000 | Malignant tumor of small intestine | SNOMED CT |
| Malignancy, except skin neoplasms | 4118989 | 302816009 | Malignant tumor of soft tissue of head, face and neck | SNOMED CT |
| Malignancy, except skin neoplasms | 4177112 | 363432004 | Malignant tumor of trachea | SNOMED CT |
| Malignancy, except skin neoplasms | 4114221 | 255052006 | Malignant tumor of unknown origin | SNOMED CT |
| Malignancy, except skin neoplasms | 4181485 | 363503004 | Malignant tumor of upper limb | SNOMED CT |
| Malignancy, except skin neoplasms | 4177113 | 363438000 | Malignant tumor of vertebral column | SNOMED CT |
| Malignancy, except skin neoplasms | 40481901 | 443487006 | Mantle cell lymphoma | SNOMED CT |
| Malignancy, except skin neoplasms | 40479608 | 441559006 | Mantle cell lymphoma of spleen | SNOMED CT |
| Malignancy, except skin neoplasms | 40490918 | 447100004 | Marginal zone lymphoma | SNOMED CT |
| Malignancy, except skin neoplasms | 36684461 | 116691000119101 | Marginal zone lymphoma of spleen | SNOMED CT |
| Malignancy, except skin neoplasms | 4002496 | 110002002 | Mast cell leukemia (clinical) | SNOMED CT |
| Malignancy, except skin neoplasms | 4264693 | 397009000 | Mast cell malignancy | SNOMED CT |
| Malignancy, except skin neoplasms | 4096968 | 188669003 | Mast cell malignancy of lymph nodes of multiple sites | SNOMED CT |
| Malignancy, except skin neoplasms | 4094542 | 188668006 | Mast cell malignancy of spleen | SNOMED CT |
| Malignancy, except skin neoplasms | 4225982 | 421418009 | Mature T-cell AND/OR NK cell neoplasm | SNOMED CT |
| Malignancy, except skin neoplasms | 313159 | 188754005 | Megakaryocytic leukemia | SNOMED CT |
| Malignancy, except skin neoplasms | 136930 | 94148006 | Megakaryocytic leukemia in remission | SNOMED CT |
| Malignancy, except skin neoplasms | 4033891 | 109378008 | Mesothelioma (malignant, clinical disorder) | SNOMED CT |
| Malignancy, except skin neoplasms | 4001666 | 109853004 | Mesothelioma of peritoneum | SNOMED CT |
| Malignancy, except skin neoplasms | 321526 | 188744006 | Monocytic leukemia | SNOMED CT |
| Malignancy, except skin neoplasms | 437233 | 109989006 | Multiple myeloma | SNOMED CT |
| Malignancy, except skin neoplasms | 436059 | 94704006 | Multiple myeloma in remission | SNOMED CT |
| Malignancy, except skin neoplasms | 4040380 | 118618005 | Mycosis fungoides (clinical) | SNOMED CT |
| Malignancy, except skin neoplasms | 135204 | 94715001 | Mycosis fungoides of extranodal AND/OR solid organ site | SNOMED CT |
| Malignancy, except skin neoplasms | 201813 | 94707004 | Mycosis fungoides of intra-abdominal lymph nodes | SNOMED CT |
| Malignancy, except skin neoplasms | 442169 | 94708009 | Mycosis fungoides of intrapelvic lymph nodes | SNOMED CT |
| Malignancy, except skin neoplasms | 134879 | 94709001 | Mycosis fungoides of intrathoracic lymph nodes | SNOMED CT |
| Malignancy, except skin neoplasms | 141524 | 94710006 | Mycosis fungoides of lymph nodes of axilla AND/OR upper limb | SNOMED CT |
| Malignancy, except skin neoplasms | 138377 | 94711005 | Mycosis fungoides of lymph nodes of head, face AND/OR neck | SNOMED CT |
| Malignancy, except skin neoplasms | 194593 | 94712003 | Mycosis fungoides of lymph nodes of inguinal region AND/OR lower limb | SNOMED CT |
| Malignancy, except skin neoplasms | 135764 | 188627002 | Mycosis fungoides of lymph nodes of multiple sites | SNOMED CT |
| Malignancy, except skin neoplasms | 442168 | 94714002 | Mycosis fungoides of spleen | SNOMED CT |
| Malignancy, except skin neoplasms | 40492268 | 445738007 | Myelodysplastic/myeloproliferative disease | SNOMED CT |
| Malignancy, except skin neoplasms | 140666 | 188732008 | Myeloid leukemia | SNOMED CT |
| Malignancy, except skin neoplasms | 760933 | 122901000119109 | Myeloid leukemia in relapse | SNOMED CT |
| Malignancy, except skin neoplasms | 132850 | 94716000 | Myeloid leukemia in remission | SNOMED CT |
| Malignancy, except skin neoplasms | 140967 | 94719007 | Myeloid sarcoma | SNOMED CT |
| Malignancy, except skin neoplasms | 132575 | 94718004 | Myeloid sarcoma in remission | SNOMED CT |
| Malignancy, except skin neoplasms | 4054513 | 126675008 | Neoplasm of accessory sinus | SNOMED CT |
| Malignancy, except skin neoplasms | 4180312 | 363227003 | Neoplasm of extremity | SNOMED CT |
| Malignancy, except skin neoplasms | 4160342 | 371481006 | Neoplasm of eye region | SNOMED CT |
| Malignancy, except skin neoplasms | 4130672 | 127016006 | Neoplasm of multiple endocrine glands | SNOMED CT |
| Malignancy, except skin neoplasms | 4131761 | 126920004 | Neoplasm of placenta | SNOMED CT |
| Malignancy, except skin neoplasms | 4308811 | 387837005 | Neoplasm of soft tissue | SNOMED CT |
| Malignancy, except skin neoplasms | 4133600 | 127230005 | Neoplasm of spleen | SNOMED CT |
| Malignancy, except skin neoplasms | 4149840 | 269476000 | Nodular lymphoma | SNOMED CT |
| Malignancy, except skin neoplasms | 194878 | 95194004 | Nodular lymphoma of extranodal AND/OR solid organ site | SNOMED CT |
| Malignancy, except skin neoplasms | 200338 | 95186006 | Nodular lymphoma of intra-abdominal lymph nodes | SNOMED CT |
| Malignancy, except skin neoplasms | 198088 | 95187002 | Nodular lymphoma of intrapelvic lymph nodes | SNOMED CT |
| Malignancy, except skin neoplasms | 435492 | 95188007 | Nodular lymphoma of intrathoracic lymph nodes | SNOMED CT |
| Malignancy, except skin neoplasms | 437505 | 188612002 | Nodular lymphoma of lymph nodes of axilla and upper limb | SNOMED CT |
| Malignancy, except skin neoplasms | 435207 | 188609000 | Nodular lymphoma of lymph nodes of head, face and neck | SNOMED CT |
| Malignancy, except skin neoplasms | 198707 | 188613007 | Nodular lymphoma of lymph nodes of inguinal region and lower limb | SNOMED CT |
| Malignancy, except skin neoplasms | 320347 | 95192000 | Nodular lymphoma of lymph nodes of multiple sites | SNOMED CT |
| Malignancy, except skin neoplasms | 198374 | 95193005 | Nodular lymphoma of spleen | SNOMED CT |
| Malignancy, except skin neoplasms | 4038838 | 118601006 | Non-Hodgkin's lymphoma (clinical) | SNOMED CT |
| Malignancy, except skin neoplasms | 40487528 | 447989004 | Non-Hodgkin's lymphoma of extranodal site | SNOMED CT |
| Malignancy, except skin neoplasms | 133419 | 109267002 | Overlapping malignant melanoma of skin | SNOMED CT |
| Malignancy, except skin neoplasms | 198695 | 109847004 | Overlapping malignant neoplasm of biliary tract | SNOMED CT |
| Malignancy, except skin neoplasms | 198082 | 109879008 | Overlapping malignant neoplasm of body of uterus | SNOMED CT |
| Malignancy, except skin neoplasms | 4032866 | 109347009 | Overlapping malignant neoplasm of bone and articular cartilage | SNOMED CT |
| Malignancy, except skin neoplasms | 4033318 | 109348004 | Overlapping malignant neoplasm of bone and articular cartilage of limbs | SNOMED CT |
| Malignancy, except skin neoplasms | 4003693 | 109912006 | Overlapping malignant neoplasm of brain | SNOMED CT |
| Malignancy, except skin neoplasms | 372849 | 109911004 | Overlapping malignant neoplasm of brain and other parts of the central nervous system | SNOMED CT |
| Malignancy, except skin neoplasms | 258375 | 109371002 | Overlapping malignant neoplasm of bronchus and lung | SNOMED CT |
| Malignancy, except skin neoplasms | 79740 | 109838007 | Overlapping malignant neoplasm of colon | SNOMED CT |
| Malignancy, except skin neoplasms | 25748 | 109835005 | Overlapping malignant neoplasm of esophagus | SNOMED CT |
| Malignancy, except skin neoplasms | 438095 | 109948008 | Overlapping malignant neoplasm of eye and adnexa (primary) | SNOMED CT |
| Malignancy, except skin neoplasms | 133711 | 109886000 | Overlapping malignant neoplasm of female breast | SNOMED CT |
| Malignancy, except skin neoplasms | 196051 | 109878000 | Overlapping malignant neoplasm of female genital organs | SNOMED CT |
| Malignancy, except skin neoplasms | 140955 | 109830000 | Overlapping malignant neoplasm of floor of mouth | SNOMED CT |
| Malignancy, except skin neoplasms | 321234 | 109384006 | Overlapping malignant neoplasm of heart, mediastinum and pleura | SNOMED CT |
| Malignancy, except skin neoplasms | 440044 | 109368005 | Overlapping malignant neoplasm of hypopharynx | SNOMED CT |
| Malignancy, except skin neoplasms | 22839 | 109369002 | Overlapping malignant neoplasm of larynx | SNOMED CT |
| Malignancy, except skin neoplasms | 133710 | 109822001 | Overlapping malignant neoplasm of lip | SNOMED CT |
| Malignancy, except skin neoplasms | 444224 | 109833003 | Overlapping malignant neoplasm of lip, oral cavity and pharynx | SNOMED CT |
| Malignancy, except skin neoplasms | 28356 | 109824000 | Overlapping malignant neoplasm of major salivary gland | SNOMED CT |
| Malignancy, except skin neoplasms | 4003684 | 109887009 | Overlapping malignant neoplasm of male breast | SNOMED CT |
| Malignancy, except skin neoplasms | 196645 | 109874003 | Overlapping malignant neoplasm of male genital organs | SNOMED CT |
| Malignancy, except skin neoplasms | 432558 | 109367000 | Overlapping malignant neoplasm of nasopharynx | SNOMED CT |
| Malignancy, except skin neoplasms | 438691 | 109832008 | Overlapping malignant neoplasm of oropharynx | SNOMED CT |
| Malignancy, except skin neoplasms | 4001170 | 109831001 | Overlapping malignant neoplasm of palate | SNOMED CT |
| Malignancy, except skin neoplasms | 192261 | 109848009 | Overlapping malignant neoplasm of pancreas | SNOMED CT |
| Malignancy, except skin neoplasms | 4002343 | 109919002 | Overlapping malignant neoplasm of peripheral nerves and autonomic nervous system | SNOMED CT |
| Malignancy, except skin neoplasms | 438090 | 109839004 | Overlapping malignant neoplasm of rectum, anus and anal canal | SNOMED CT |
| Malignancy, except skin neoplasms | 198092 | 109851002 | Overlapping malignant neoplasm of retroperitoneum and peritoneum | SNOMED CT |
| Malignancy, except skin neoplasms | 199747 | 109837002 | Overlapping malignant neoplasm of small intestine | SNOMED CT |
| Malignancy, except skin neoplasms | 378081 | 109349007 | Overlapping malignant neoplasm of soft tissues | SNOMED CT |
| Malignancy, except skin neoplasms | 197803 | 109836006 | Overlapping malignant neoplasm of stomach | SNOMED CT |
| Malignancy, except skin neoplasms | 436043 | 109823006 | Overlapping malignant neoplasm of tongue | SNOMED CT |
| Malignancy, except skin neoplasms | 4002498 | 110013004 | Overlapping malignant neoplasm of tonsil | SNOMED CT |
| Malignancy, except skin neoplasms | 4003029 | 109885001 | Overlapping malignant neoplasm of vulva | SNOMED CT |
| Malignancy, except skin neoplasms | 434881 | 109977009 | Peripheral T-cell lymphoma (clinical) | SNOMED CT |
| Malignancy, except skin neoplasms | 133154 | 95210003 | Plasma cell leukemia | SNOMED CT |
| Malignancy, except skin neoplasms | 760936 | 122981000119101 | Plasma cell leukemia in relapse | SNOMED CT |
| Malignancy, except skin neoplasms | 133158 | 95209008 | Plasma cell leukemia in remission | SNOMED CT |
| Malignancy, except skin neoplasms | 443743 | 415111003 | Plasma cell neoplasm | SNOMED CT |
| Malignancy, except skin neoplasms | 4216139 | 415112005 | Plasmacytoma | SNOMED CT |
| Malignancy, except skin neoplasms | 135214 | 109992005 | Polycythemia vera (clinical) | SNOMED CT |
| Malignancy, except skin neoplasms | 373152 | 307649006 | Primary central nervous system lymphoma | SNOMED CT |
| Malignancy, except skin neoplasms | 4301668 | 404143002 | Primary cutaneous follicular center B-cell lymphoma | SNOMED CT |
| Malignancy, except skin neoplasms | 36716501 | 722529000 | Primary malignant epithelial neoplasm of nasopharynx | SNOMED CT |
| Malignancy, except skin neoplasms | 439392 | 372087000 | Primary malignant neoplasm | SNOMED CT |
| Malignancy, except skin neoplasms | 36684472 | 148911000119107 | Primary malignant neoplasm of abdomen | SNOMED CT |
| Malignancy, except skin neoplasms | 193971 | 371962007 | Primary malignant neoplasm of abdominal esophagus | SNOMED CT |
| Malignancy, except skin neoplasms | 259748 | 93659005 | Primary malignant neoplasm of accessory sinus | SNOMED CT |
| Malignancy, except skin neoplasms | 4162115 | 371963002 | Primary malignant neoplasm of adrenal cortex | SNOMED CT |
| Malignancy, except skin neoplasms | 198104 | 93665005 | Primary malignant neoplasm of adrenal gland | SNOMED CT |
| Malignancy, except skin neoplasms | 4162859 | 371966005 | Primary malignant neoplasm of adrenal medulla | SNOMED CT |
| Malignancy, except skin neoplasms | 441225 | 371967001 | Primary malignant neoplasm of ampulla of Vater | SNOMED CT |
| Malignancy, except skin neoplasms | 436348 | 93669004 | Primary malignant neoplasm of anal canal | SNOMED CT |
| Malignancy, except skin neoplasms | 436643 | 93670003 | Primary malignant neoplasm of anterior aspect of epiglottis | SNOMED CT |
| Malignancy, except skin neoplasms | 432844 | 93671004 | Primary malignant neoplasm of anterior mediastinum | SNOMED CT |
| Malignancy, except skin neoplasms | 438982 | 93672006 | Primary malignant neoplasm of anterior portion of floor of mouth | SNOMED CT |
| Malignancy, except skin neoplasms | 436042 | 371968006 | Primary malignant neoplasm of anterior two-thirds of tongue | SNOMED CT |
| Malignancy, except skin neoplasms | 438080 | 93674007 | Primary malignant neoplasm of anterior wall of nasopharynx | SNOMED CT |
| Malignancy, except skin neoplasms | 40649300 | 93675008 | Primary malignant neoplasm of anterior wall of urinary bladder | SNOMED CT |
| Malignancy, except skin neoplasms | 80045 | 93676009 | Primary malignant neoplasm of anus | SNOMED CT |
| Malignancy, except skin neoplasms | 433143 | 93679002 | Primary malignant neoplasm of appendix | SNOMED CT |
| Malignancy, except skin neoplasms | 444203 | 128466006 | Primary malignant neoplasm of articular cartilage | SNOMED CT |
| Malignancy, except skin neoplasms | 4247719 | 93683002 | Primary malignant neoplasm of ascending colon | SNOMED CT |
| Malignancy, except skin neoplasms | 441513 | 372092003 | Primary malignant neoplasm of axillary tail of breast | SNOMED CT |
| Malignancy, except skin neoplasms | 36684817 | 353421000119109 | Primary malignant neoplasm of axillary tail of left female breast | SNOMED CT |
| Malignancy, except skin neoplasms | 36684820 | 353501000119104 | Primary malignant neoplasm of axillary tail of right female breast | SNOMED CT |
| Malignancy, except skin neoplasms | 256633 | 93687001 | Primary malignant neoplasm of base of tongue | SNOMED CT |
| Malignancy, except skin neoplasms | 194589 | 371970002 | Primary malignant neoplasm of biliary tract | SNOMED CT |
| Malignancy, except skin neoplasms | 196360 | 93689003 | Primary malignant neoplasm of bladder | SNOMED CT |
| Malignancy, except skin neoplasms | 434293 | 93715005 | Primary malignant neoplasm of body of pancreas | SNOMED CT |
| Malignancy, except skin neoplasms | 79749 | 93716006 | Primary malignant neoplasm of body of penis | SNOMED CT |
| Malignancy, except skin neoplasms | 193422 | 93717002 | Primary malignant neoplasm of body of stomach | SNOMED CT |
| Malignancy, except skin neoplasms | 4162860 | 371971003 | Primary malignant neoplasm of body of uterus | SNOMED CT |
| Malignancy, except skin neoplasms | 76914 | 93725000 | Primary malignant neoplasm of bone | SNOMED CT |
| Malignancy, except skin neoplasms | 4246802 | 93723007 | Primary malignant neoplasm of bone of skull | SNOMED CT |
| Malignancy, except skin neoplasms | 4156114 | 371975007 | Primary malignant neoplasm of border of tongue | SNOMED CT |
| Malignancy, except skin neoplasms | 380055 | 93727008 | Primary malignant neoplasm of brain | SNOMED CT |
| Malignancy, except skin neoplasms | 441806 | 93726004 | Primary malignant neoplasm of brain stem | SNOMED CT |
| Malignancy, except skin neoplasms | 4003674 | 109834009 | Primary malignant neoplasm of branchial cleft | SNOMED CT |
| Malignancy, except skin neoplasms | 4162253 | 372137005 | Primary malignant neoplasm of breast | SNOMED CT |
| Malignancy, except skin neoplasms | 4187851 | 373090000 | Primary malignant neoplasm of breast lower inner quadrant | SNOMED CT |
| Malignancy, except skin neoplasms | 4188545 | 373091001 | Primary malignant neoplasm of breast lower outer quadrant | SNOMED CT |
| Malignancy, except skin neoplasms | 4158563 | 373089009 | Primary malignant neoplasm of breast upper inner quadrant | SNOMED CT |
| Malignancy, except skin neoplasms | 4187850 | 373088001 | Primary malignant neoplasm of breast upper outer quadrant | SNOMED CT |
| Malignancy, except skin neoplasms | 432264 | 93728003 | Primary malignant neoplasm of broad ligament | SNOMED CT |
| Malignancy, except skin neoplasms | 134579 | 371976008 | Primary malignant neoplasm of buccal mucosa | SNOMED CT |
| Malignancy, except skin neoplasms | 432838 | 93738008 | Primary malignant neoplasm of cardia of stomach | SNOMED CT |
| Malignancy, except skin neoplasms | 27235 | 93740003 | Primary malignant neoplasm of carotid body | SNOMED CT |
| Malignancy, except skin neoplasms | 4246029 | 93743001 | Primary malignant neoplasm of cauda equina | SNOMED CT |
| Malignancy, except skin neoplasms | 432837 | 371977004 | Primary malignant neoplasm of cecum | SNOMED CT |
| Malignancy, except skin neoplasms | 4247822 | 93744007 | Primary malignant neoplasm of central nervous system | SNOMED CT |
| Malignancy, except skin neoplasms | 432845 | 93745008 | Primary malignant neoplasm of central portion of female breast | SNOMED CT |
| Malignancy, except skin neoplasms | 433149 | 93746009 | Primary malignant neoplasm of cerebellum | SNOMED CT |
| Malignancy, except skin neoplasms | 436926 | 93747000 | Primary malignant neoplasm of cerebral meninges | SNOMED CT |
| Malignancy, except skin neoplasms | 432848 | 93748005 | Primary malignant neoplasm of cerebral ventricle | SNOMED CT |
| Malignancy, except skin neoplasms | 380661 | 93749002 | Primary malignant neoplasm of cerebrum | SNOMED CT |
| Malignancy, except skin neoplasms | 441224 | 371978009 | Primary malignant neoplasm of cervical esophagus | SNOMED CT |
| Malignancy, except skin neoplasms | 378696 | 93755007 | Primary malignant neoplasm of choroid | SNOMED CT |
| Malignancy, except skin neoplasms | 4246808 | 93756008 | Primary malignant neoplasm of ciliary body (primary) | SNOMED CT |
| Malignancy, except skin neoplasms | 197225 | 371980003 | Primary malignant neoplasm of clitoris | SNOMED CT |
| Malignancy, except skin neoplasms | 4003675 | 109840002 | Primary malignant neoplasm of cloacogenic zone | SNOMED CT |
| Malignancy, except skin neoplasms | 197500 | 93761005 | Primary malignant neoplasm of colon | SNOMED CT |
| Malignancy, except skin neoplasms | 255192 | 371981004 | Primary malignant neoplasm of commissure of lip | SNOMED CT |
| Malignancy, except skin neoplasms | 373151 | 93764002 | Primary malignant neoplasm of conjunctiva (primary) | SNOMED CT |
| Malignancy, except skin neoplasms | 375490 | 93766000 | Primary malignant neoplasm of cornea (primary) | SNOMED CT |
| Malignancy, except skin neoplasms | 433975 | 93767009 | Primary malignant neoplasm of cranial nerve | SNOMED CT |
| Malignancy, except skin neoplasms | 442134 | 93768004 | Primary malignant neoplasm of craniopharyngeal duct | SNOMED CT |
| Malignancy, except skin neoplasms | 4003028 | 109876001 | Primary malignant neoplasm of descended testis | SNOMED CT |
| Malignancy, except skin neoplasms | 441800 | 93771007 | Primary malignant neoplasm of descending colon | SNOMED CT |
| Malignancy, except skin neoplasms | 42709931 | 449803009 | Primary malignant neoplasm of dome of urinary bladder | SNOMED CT |
| Malignancy, except skin neoplasms | 434587 | 93773005 | Primary malignant neoplasm of dorsal surface of tongue | SNOMED CT |
| Malignancy, except skin neoplasms | 435752 | 93775003 | Primary malignant neoplasm of duodenum | SNOMED CT |
| Malignancy, except skin neoplasms | 441805 | 93779009 | Primary malignant neoplasm of endocervix | SNOMED CT |
| Malignancy, except skin neoplasms | 133420 | 371983001 | Primary malignant neoplasm of endocrine gland | SNOMED CT |
| Malignancy, except skin neoplasms | 4247238 | 93781006 | Primary malignant neoplasm of endometrium | SNOMED CT |
| Malignancy, except skin neoplasms | 80340 | 93783009 | Primary malignant neoplasm of epididymis | SNOMED CT |
| Malignancy, except skin neoplasms | 26638 | 371984007 | Primary malignant neoplasm of esophagus | SNOMED CT |
| Malignancy, except skin neoplasms | 140046 | 93787005 | Primary malignant neoplasm of ethmoidal sinus | SNOMED CT |
| Malignancy, except skin neoplasms | 436358 | 93789008 | Primary malignant neoplasm of exocervix | SNOMED CT |
| Malignancy, except skin neoplasms | 40486896 | 446189008 | Primary malignant neoplasm of extrahepatic bile duct | SNOMED CT |
| Malignancy, except skin neoplasms | 374874 | 371986009 | Primary malignant neoplasm of eye | SNOMED CT |
| Malignancy, except skin neoplasms | 201801 | 371987000 | Primary malignant neoplasm of fallopian tube | SNOMED CT |
| Malignancy, except skin neoplasms | 137809 | 93796005 | Primary malignant neoplasm of female breast | SNOMED CT |
| Malignancy, except skin neoplasms | 201238 | 93797001 | Primary malignant neoplasm of female genital organ | SNOMED CT |
| Malignancy, except skin neoplasms | 135750 | 93802007 | Primary malignant neoplasm of floor of mouth | SNOMED CT |
| Malignancy, except skin neoplasms | 441233 | 93807001 | Primary malignant neoplasm of frontal lobe | SNOMED CT |
| Malignancy, except skin neoplasms | 132258 | 93808006 | Primary malignant neoplasm of frontal sinus | SNOMED CT |
| Malignancy, except skin neoplasms | 435751 | 93809003 | Primary malignant neoplasm of fundus of stomach | SNOMED CT |
| Malignancy, except skin neoplasms | 197806 | 372139008 | Primary malignant neoplasm of gallbladder | SNOMED CT |
| Malignancy, except skin neoplasms | 4179720 | 363745004 | Primary malignant neoplasm of gastrointestinal tract | SNOMED CT |
| Malignancy, except skin neoplasms | 438370 | 371989002 | Primary malignant neoplasm of glans penis | SNOMED CT |
| Malignancy, except skin neoplasms | 260336 | 93816002 | Primary malignant neoplasm of glottis | SNOMED CT |
| Malignancy, except skin neoplasms | 437224 | 93818001 | Primary malignant neoplasm of greater curvature of stomach | SNOMED CT |
| Malignancy, except skin neoplasms | 140950 | 371990006 | Primary malignant neoplasm of gum | SNOMED CT |
| Malignancy, except skin neoplasms | 438694 | 371991005 | Primary malignant neoplasm of hard palate | SNOMED CT |
| Malignancy, except skin neoplasms | 442131 | 93824007 | Primary malignant neoplasm of head | SNOMED CT |
| Malignancy, except skin neoplasms | 440649 | 372119009 | Primary malignant neoplasm of head of pancreas | SNOMED CT |
| Malignancy, except skin neoplasms | 316644 | 93825008 | Primary malignant neoplasm of heart | SNOMED CT |
| Malignancy, except skin neoplasms | 438979 | 93826009 | Primary malignant neoplasm of hepatic flexure of colon | SNOMED CT |
| Malignancy, except skin neoplasms | 434577 | 93829002 | Primary malignant neoplasm of hypopharyngeal aspect of aryepiglottic fold | SNOMED CT |
| Malignancy, except skin neoplasms | 439746 | 93831006 | Primary malignant neoplasm of hypopharynx | SNOMED CT |
| Malignancy, except skin neoplasms | 4311480 | 93832004 | Primary malignant neoplasm of ileum | SNOMED CT |
| Malignancy, except skin neoplasms | 435485 | 109357005 | Primary malignant neoplasm of ill-defined site | SNOMED CT |
| Malignancy, except skin neoplasms | 132832 | 93835002 | Primary malignant neoplasm of inner aspect of lip | SNOMED CT |
| Malignancy, except skin neoplasms | 137219 | 93836001 | Primary malignant neoplasm of inner aspect of lower lip | SNOMED CT |
| Malignancy, except skin neoplasms | 138351 | 93837005 | Primary malignant neoplasm of inner aspect of upper lip | SNOMED CT |
| Malignancy, except skin neoplasms | 195482 | 371992003 | Primary malignant neoplasm of intestinal tract | SNOMED CT |
| Malignancy, except skin neoplasms | 197804 | 93839008 | Primary malignant neoplasm of intra-abdominal organs | SNOMED CT |
| Malignancy, except skin neoplasms | 40490929 | 447109003 | Primary malignant neoplasm of intrahepatic bile duct | SNOMED CT |
| Malignancy, except skin neoplasms | 4247331 | 93841009 | Primary malignant neoplasm of intrathoracic organs | SNOMED CT |
| Malignancy, except skin neoplasms | 25486 | 93843007 | Primary malignant neoplasm of islets of Langerhans | SNOMED CT |
| Malignancy, except skin neoplasms | 192847 | 93844001 | Primary malignant neoplasm of isthmus of uterus | SNOMED CT |
| Malignancy, except skin neoplasms | 436913 | 93846004 | Primary malignant neoplasm of jejunum | SNOMED CT |
| Malignancy, except skin neoplasms | 440036 | 93848003 | Primary malignant neoplasm of junctional zone of tongue | SNOMED CT |
| Malignancy, except skin neoplasms | 198985 | 93849006 | Primary malignant neoplasm of kidney | SNOMED CT |
| Malignancy, except skin neoplasms | 437501 | 93850006 | Primary malignant neoplasm of labia majora | SNOMED CT |
| Malignancy, except skin neoplasms | 441802 | 93851005 | Primary malignant neoplasm of labia minora | SNOMED CT |
| Malignancy, except skin neoplasms | 4313056 | 423195009 | Primary malignant neoplasm of lacrimal drainage system | SNOMED CT |
| Malignancy, except skin neoplasms | 372567 | 371993008 | Primary malignant neoplasm of lacrimal gland | SNOMED CT |
| Malignancy, except skin neoplasms | 4246125 | 93854002 | Primary malignant neoplasm of large intestine | SNOMED CT |
| Malignancy, except skin neoplasms | 436352 | 109370001 | Primary malignant neoplasm of laryngeal cartilage | SNOMED CT |
| Malignancy, except skin neoplasms | 26052 | 371995001 | Primary malignant neoplasm of larynx | SNOMED CT |
| Malignancy, except skin neoplasms | 440344 | 93860002 | Primary malignant neoplasm of lateral portion of floor of mouth | SNOMED CT |
| Malignancy, except skin neoplasms | 438692 | 93861003 | Primary malignant neoplasm of lateral wall of nasopharynx | SNOMED CT |
| Malignancy, except skin neoplasms | 440345 | 93862005 | Primary malignant neoplasm of lateral wall of oropharynx | SNOMED CT |
| Malignancy, except skin neoplasms | 40650072 | 93863000 | Primary malignant neoplasm of lateral wall of urinary bladder | SNOMED CT |
| Malignancy, except skin neoplasms | 434292 | 93867004 | Primary malignant neoplasm of lesser curvature of stomach | SNOMED CT |
| Malignancy, except skin neoplasms | 434289 | 93868009 | Primary malignant neoplasm of lingual tonsil | SNOMED CT |
| Malignancy, except skin neoplasms | 4155171 | 371996000 | Primary malignant neoplasm of lip | SNOMED CT |
| Malignancy, except skin neoplasms | 201519 | 95214007 | Primary malignant neoplasm of liver | SNOMED CT |
| Malignancy, except skin neoplasms | 436640 | 93871001 | Primary malignant neoplasm of long bone of lower limb | SNOMED CT |
| Malignancy, except skin neoplasms | 440335 | 371997009 | Primary malignant neoplasm of lower gum | SNOMED CT |
| Malignancy, except skin neoplasms | 432263 | 93874009 | Primary malignant neoplasm of lower inner quadrant of female breast | SNOMED CT |
| Malignancy, except skin neoplasms | 201518 | 93875005 | Primary malignant neoplasm of lower limb | SNOMED CT |
| Malignancy, except skin neoplasms | 4157454 | 372110008 | Primary malignant neoplasm of lower lobe, bronchus or lung | SNOMED CT |
| Malignancy, except skin neoplasms | 441515 | 93876006 | Primary malignant neoplasm of lower outer quadrant of female breast | SNOMED CT |
| Malignancy, except skin neoplasms | 193138 | 371998004 | Primary malignant neoplasm of lower third of esophagus | SNOMED CT |
| Malignancy, except skin neoplasms | 257503 | 93882009 | Primary malignant neoplasm of main bronchus | SNOMED CT |
| Malignancy, except skin neoplasms | 4247836 | 93883004 | Primary malignant neoplasm of major salivary gland | SNOMED CT |
| Malignancy, except skin neoplasms | 135489 | 93884005 | Primary malignant neoplasm of male breast | SNOMED CT |
| Malignancy, except skin neoplasms | 197507 | 93885006 | Primary malignant neoplasm of male genital organ | SNOMED CT |
| Malignancy, except skin neoplasms | 379756 | 93886007 | Primary malignant neoplasm of mandible | SNOMED CT |
| Malignancy, except skin neoplasms | 137800 | 93889000 | Primary malignant neoplasm of maxillary sinus | SNOMED CT |
| Malignancy, except skin neoplasms | 432256 | 93890009 | Primary malignant neoplasm of Meckel's diverticulum | SNOMED CT |
| Malignancy, except skin neoplasms | 438693 | 93891008 | Primary malignant neoplasm of mediastinum | SNOMED CT |
| Malignancy, except skin neoplasms | 4002340 | 109915008 | Primary malignant neoplasm of meninges | SNOMED CT |
| Malignancy, except skin neoplasms | 4247336 | 93894000 | Primary malignant neoplasm of middle ear | SNOMED CT |
| Malignancy, except skin neoplasms | 256646 | 372112000 | Primary malignant neoplasm of middle lobe, bronchus or lung | SNOMED CT |
| Malignancy, except skin neoplasms | 437805 | 371999007 | Primary malignant neoplasm of middle third of esophagus | SNOMED CT |
| Malignancy, except skin neoplasms | 4247842 | 93915004 | Primary malignant neoplasm of myometrium | SNOMED CT |
| Malignancy, except skin neoplasms | 438367 | 93917007 | Primary malignant neoplasm of nasal cavity | SNOMED CT |
| Malignancy, except skin neoplasms | 36684473 | 226521000119108 | Primary malignant neoplasm of nasopharynx | SNOMED CT |
| Malignancy, except skin neoplasms | 376918 | 93923002 | Primary malignant neoplasm of nervous system | SNOMED CT |
| Malignancy, except skin neoplasms | 432559 | 93928006 | Primary malignant neoplasm of occipital lobe | SNOMED CT |
| Malignancy, except skin neoplasms | 4246137 | 93931007 | Primary malignant neoplasm of optic nerve | SNOMED CT |
| Malignancy, except skin neoplasms | 439404 | 372001002 | Primary malignant neoplasm of oral cavity | SNOMED CT |
| Malignancy, except skin neoplasms | 441809 | 93932000 | Primary malignant neoplasm of orbit | SNOMED CT |
| Malignancy, except skin neoplasms | 432833 | 93933005 | Primary malignant neoplasm of oropharynx | SNOMED CT |
| Malignancy, except skin neoplasms | 200051 | 93934004 | Primary malignant neoplasm of ovary | SNOMED CT |
| Malignancy, except skin neoplasms | 134290 | 372002009 | Primary malignant neoplasm of palate | SNOMED CT |
| Malignancy, except skin neoplasms | 199754 | 372003004 | Primary malignant neoplasm of pancreas | SNOMED CT |
| Malignancy, except skin neoplasms | 433423 | 93939009 | Primary malignant neoplasm of pancreatic duct | SNOMED CT |
| Malignancy, except skin neoplasms | 442122 | 93941005 | Primary malignant neoplasm of paraganglion | SNOMED CT |
| Malignancy, except skin neoplasms | 196047 | 93942003 | Primary malignant neoplasm of parametrium | SNOMED CT |
| Malignancy, except skin neoplasms | 139753 | 93943008 | Primary malignant neoplasm of parathyroid gland | SNOMED CT |
| Malignancy, except skin neoplasms | 200963 | 93944002 | Primary malignant neoplasm of paraurethral glands | SNOMED CT |
| Malignancy, except skin neoplasms | 438086 | 93946000 | Primary malignant neoplasm of parietal lobe | SNOMED CT |
| Malignancy, except skin neoplasms | 441510 | 93948004 | Primary malignant neoplasm of parietal pleura | SNOMED CT |
| Malignancy, except skin neoplasms | 434588 | 372004005 | Primary malignant neoplasm of parotid gland | SNOMED CT |
| Malignancy, except skin neoplasms | 4246141 | 93951006 | Primary malignant neoplasm of pelvic bone | SNOMED CT |
| Malignancy, except skin neoplasms | 72566 | 372115003 | Primary malignant neoplasm of pelvic bones, sacrum and coccyx | SNOMED CT |
| Malignancy, except skin neoplasms | 198988 | 93953009 | Primary malignant neoplasm of pelvis | SNOMED CT |
| Malignancy, except skin neoplasms | 195483 | 372005006 | Primary malignant neoplasm of penis | SNOMED CT |
| Malignancy, except skin neoplasms | 4307721 | 422736007 | Primary malignant neoplasm of peripheral nerve | SNOMED CT |
| Malignancy, except skin neoplasms | 4003694 | 109921007 | Primary malignant neoplasm of peripheral nerve of head, face AND/OR neck | SNOMED CT |
| Malignancy, except skin neoplasms | 4003179 | 109947003 | Primary malignant neoplasm of peripheral nerves of trunk | SNOMED CT |
| Malignancy, except skin neoplasms | 4003175 | 109931000 | Primary malignant neoplasm of peripheral nerves of upper limb | SNOMED CT |
| Malignancy, except skin neoplasms | 28083 | 93961004 | Primary malignant neoplasm of pharynx | SNOMED CT |
| Malignancy, except skin neoplasms | 26361 | 93962006 | Primary malignant neoplasm of pineal gland | SNOMED CT |
| Malignancy, except skin neoplasms | 24296 | 93964007 | Primary malignant neoplasm of pituitary gland | SNOMED CT |
| Malignancy, except skin neoplasms | 36715801 | 721567004 | Primary malignant neoplasm of placenta | SNOMED CT |
| Malignancy, except skin neoplasms | 78093 | 93966009 | Primary malignant neoplasm of pleura | SNOMED CT |
| Malignancy, except skin neoplasms | 436922 | 93967000 | Primary malignant neoplasm of postcricoid region | SNOMED CT |
| Malignancy, except skin neoplasms | 435474 | 93968005 | Primary malignant neoplasm of posterior hypopharyngeal wall | SNOMED CT |
| Malignancy, except skin neoplasms | 435487 | 93969002 | Primary malignant neoplasm of posterior mediastinum | SNOMED CT |
| Malignancy, except skin neoplasms | 436344 | 93970001 | Primary malignant neoplasm of posterior wall of nasopharynx | SNOMED CT |
| Malignancy, except skin neoplasms | 439739 | 93971002 | Primary malignant neoplasm of posterior wall of oropharynx | SNOMED CT |
| Malignancy, except skin neoplasms | 40650479 | 93972009 | Primary malignant neoplasm of posterior wall of urinary bladder | SNOMED CT |
| Malignancy, except skin neoplasms | 440339 | 372006007 | Primary malignant neoplasm of prepuce | SNOMED CT |
| Malignancy, except skin neoplasms | 200962 | 93974005 | Primary malignant neoplasm of prostate | SNOMED CT |
| Malignancy, except skin neoplasms | 438089 | 93976007 | Primary malignant neoplasm of pyloric antrum | SNOMED CT |
| Malignancy, except skin neoplasms | 192255 | 93977003 | Primary malignant neoplasm of pylorus | SNOMED CT |
| Malignancy, except skin neoplasms | 435190 | 93978008 | Primary malignant neoplasm of pyriform sinus | SNOMED CT |
| Malignancy, except skin neoplasms | 438699 | 93980002 | Primary malignant neoplasm of rectosigmoid junction | SNOMED CT |
| Malignancy, except skin neoplasms | 74582 | 93984006 | Primary malignant neoplasm of rectum | SNOMED CT |
| Malignancy, except skin neoplasms | 195480 | 93985007 | Primary malignant neoplasm of renal pelvis | SNOMED CT |
| Malignancy, except skin neoplasms | 4311499 | 93986008 | Primary malignant neoplasm of respiratory tract | SNOMED CT |
| Malignancy, except skin neoplasms | 377811 | 93987004 | Primary malignant neoplasm of retina (primary) | SNOMED CT |
| Malignancy, except skin neoplasms | 433704 | 93989001 | Primary malignant neoplasm of retromolar area | SNOMED CT |
| Malignancy, except skin neoplasms | 198091 | 94092006 | Primary malignant neoplasm of retroperitoneum | SNOMED CT |
| Malignancy, except skin neoplasms | 438977 | 372107001 | Primary malignant neoplasm of ribs and/or sternum and/or clavicle | SNOMED CT |
| Malignancy, except skin neoplasms | 441520 | 93994001 | Primary malignant neoplasm of round ligament of uterus | SNOMED CT |
| Malignancy, except skin neoplasms | 79758 | 372009000 | Primary malignant neoplasm of scrotum | SNOMED CT |
| Malignancy, except skin neoplasms | 434880 | 94003005 | Primary malignant neoplasm of short bone of lower limb | SNOMED CT |
| Malignancy, except skin neoplasms | 435493 | 94004004 | Primary malignant neoplasm of short bone of upper limb | SNOMED CT |
| Malignancy, except skin neoplasms | 436635 | 94006002 | Primary malignant neoplasm of sigmoid colon | SNOMED CT |
| Malignancy, except skin neoplasms | 192836 | 94048009 | Primary malignant neoplasm of small intestine | SNOMED CT |
| Malignancy, except skin neoplasms | 254282 | 94049001 | Primary malignant neoplasm of soft palate | SNOMED CT |
| Malignancy, except skin neoplasms | 376647 | 372010005 | Primary malignant neoplasm of soft tissues | SNOMED CT |
| Malignancy, except skin neoplasms | 197807 | 94050001 | Primary malignant neoplasm of soft tissues of abdomen | SNOMED CT |
| Malignancy, except skin neoplasms | 438094 | 94057003 | Primary malignant neoplasm of soft tissues of lower limb | SNOMED CT |
| Malignancy, except skin neoplasms | 4247358 | 94059000 | Primary malignant neoplasm of soft tissues of pelvis | SNOMED CT |
| Malignancy, except skin neoplasms | 317801 | 94062002 | Primary malignant neoplasm of soft tissues of thorax | SNOMED CT |
| Malignancy, except skin neoplasms | 197808 | 94063007 | Primary malignant neoplasm of soft tissues of trunk | SNOMED CT |
| Malignancy, except skin neoplasms | 436357 | 372012002 | Primary malignant neoplasm of soft tissues of upper limb | SNOMED CT |
| Malignancy, except skin neoplasms | 193719 | 372013007 | Primary malignant neoplasm of spermatic cord | SNOMED CT |
| Malignancy, except skin neoplasms | 136639 | 94067008 | Primary malignant neoplasm of sphenoidal sinus | SNOMED CT |
| Malignancy, except skin neoplasms | 135491 | 94068003 | Primary malignant neoplasm of spinal cord | SNOMED CT |
| Malignancy, except skin neoplasms | 134295 | 94069006 | Primary malignant neoplasm of spinal meninges | SNOMED CT |
| Malignancy, except skin neoplasms | 193418 | 94071006 | Primary malignant neoplasm of spleen | SNOMED CT |
| Malignancy, except skin neoplasms | 437798 | 94072004 | Primary malignant neoplasm of splenic flexure of colon | SNOMED CT |
| Malignancy, except skin neoplasms | 196044 | 372014001 | Primary malignant neoplasm of stomach | SNOMED CT |
| Malignancy, except skin neoplasms | 259755 | 94075002 | Primary malignant neoplasm of subglottis | SNOMED CT |
| Malignancy, except skin neoplasms | 439738 | 94076001 | Primary malignant neoplasm of sublingual gland | SNOMED CT |
| Malignancy, except skin neoplasms | 4312691 | 94077005 | Primary malignant neoplasm of submaxillary gland | SNOMED CT |
| Malignancy, except skin neoplasms | 441223 | 94078000 | Primary malignant neoplasm of superior wall of nasopharynx | SNOMED CT |
| Malignancy, except skin neoplasms | 261514 | 94080006 | Primary malignant neoplasm of supraglottis | SNOMED CT |
| Malignancy, except skin neoplasms | 432843 | 94082003 | Primary malignant neoplasm of tail of pancreas | SNOMED CT |
| Malignancy, except skin neoplasms | 433976 | 94086000 | Primary malignant neoplasm of temporal lobe | SNOMED CT |
| Malignancy, except skin neoplasms | 433716 | 94087009 | Primary malignant neoplasm of testis | SNOMED CT |
| Malignancy, except skin neoplasms | 197799 | 372016004 | Primary malignant neoplasm of the peritoneum | SNOMED CT |
| Malignancy, except skin neoplasms | 135476 | 372017008 | Primary malignant neoplasm of thoracic esophagus | SNOMED CT |
| Malignancy, except skin neoplasms | 438368 | 94096009 | Primary malignant neoplasm of thymus | SNOMED CT |
| Malignancy, except skin neoplasms | 133424 | 94098005 | Primary malignant neoplasm of thyroid gland | SNOMED CT |
| Malignancy, except skin neoplasms | 437498 | 94101009 | Primary malignant neoplasm of tongue | SNOMED CT |
| Malignancy, except skin neoplasms | 31509 | 372020000 | Primary malignant neoplasm of tonsil | SNOMED CT |
| Malignancy, except skin neoplasms | 433709 | 94102002 | Primary malignant neoplasm of tonsillar fossa | SNOMED CT |
| Malignancy, except skin neoplasms | 440047 | 94103007 | Primary malignant neoplasm of tonsillar pillar | SNOMED CT |
| Malignancy, except skin neoplasms | 432262 | 94104001 | Primary malignant neoplasm of trachea | SNOMED CT |
| Malignancy, except skin neoplasms | 432257 | 94105000 | Primary malignant neoplasm of transverse colon | SNOMED CT |
| Malignancy, except skin neoplasms | 435484 | 94109006 | Primary malignant neoplasm of trigone of urinary bladder | SNOMED CT |
| Malignancy, except skin neoplasms | 4312698 | 94111002 | Primary malignant neoplasm of trunk | SNOMED CT |
| Malignancy, except skin neoplasms | 436054 | 94113004 | Primary malignant neoplasm of undescended testis | SNOMED CT |
| Malignancy, except skin neoplasms | 435478 | 372022008 | Primary malignant neoplasm of upper gum | SNOMED CT |
| Malignancy, except skin neoplasms | 440956 | 94115006 | Primary malignant neoplasm of upper inner quadrant of female breast | SNOMED CT |
| Malignancy, except skin neoplasms | 81239 | 94116007 | Primary malignant neoplasm of upper limb | SNOMED CT |
| Malignancy, except skin neoplasms | 81237 | 372133009 | Primary malignant neoplasm of upper limb bones and scapula | SNOMED CT |
| Malignancy, except skin neoplasms | 261236 | 372135002 | Primary malignant neoplasm of upper lobe, bronchus or lung | SNOMED CT |
| Malignancy, except skin neoplasms | 436353 | 94117003 | Primary malignant neoplasm of upper outer quadrant of female breast | SNOMED CT |
| Malignancy, except skin neoplasms | 252840 | 94118008 | Primary malignant neoplasm of upper respiratory tract | SNOMED CT |
| Malignancy, except skin neoplasms | 432260 | 372023003 | Primary malignant neoplasm of upper third of esophagus | SNOMED CT |
| Malignancy, except skin neoplasms | 201517 | 94120006 | Primary malignant neoplasm of urachus | SNOMED CT |
| Malignancy, except skin neoplasms | 200054 | 94121005 | Primary malignant neoplasm of ureter | SNOMED CT |
| Malignancy, except skin neoplasms | 76924 | 94122003 | Primary malignant neoplasm of ureteric orifice of urinary bladder | SNOMED CT |
| Malignancy, except skin neoplasms | 196049 | 94123008 | Primary malignant neoplasm of urethra | SNOMED CT |
| Malignancy, except skin neoplasms | 436923 | 94124002 | Primary malignant neoplasm of urinary bladder neck | SNOMED CT |
| Malignancy, except skin neoplasms | 76349 | 94125001 | Primary malignant neoplasm of urinary system | SNOMED CT |
| Malignancy, except skin neoplasms | 200052 | 94126000 | Primary malignant neoplasm of uterine adnexa | SNOMED CT |
| Malignancy, except skin neoplasms | 196359 | 372024009 | Primary malignant neoplasm of uterine cervix | SNOMED CT |
| Malignancy, except skin neoplasms | 45770892 | 10708511000119100 | Primary malignant neoplasm of uterus | SNOMED CT |
| Malignancy, except skin neoplasms | 434285 | 94129007 | Primary malignant neoplasm of uvula | SNOMED CT |
| Malignancy, except skin neoplasms | 196048 | 372025005 | Primary malignant neoplasm of vagina | SNOMED CT |
| Malignancy, except skin neoplasms | 438360 | 94132005 | Primary malignant neoplasm of vallecula | SNOMED CT |
| Malignancy, except skin neoplasms | 437220 | 94134006 | Primary malignant neoplasm of ventral surface of tongue | SNOMED CT |
| Malignancy, except skin neoplasms | 133969 | 94135007 | Primary malignant neoplasm of vermilion border of lip | SNOMED CT |
| Malignancy, except skin neoplasms | 132565 | 372026006 | Primary malignant neoplasm of vermilion border of lower lip | SNOMED CT |
| Malignancy, except skin neoplasms | 138074 | 372027002 | Primary malignant neoplasm of vermilion border of upper lip | SNOMED CT |
| Malignancy, except skin neoplasms | 75488 | 372028007 | Primary malignant neoplasm of vertebral column | SNOMED CT |
| Malignancy, except skin neoplasms | 261808 | 94138009 | Primary malignant neoplasm of vestibule of mouth | SNOMED CT |
| Malignancy, except skin neoplasms | 441230 | 94140004 | Primary malignant neoplasm of visceral pleura | SNOMED CT |
| Malignancy, except skin neoplasms | 195197 | 94143002 | Primary malignant neoplasm of vulva | SNOMED CT |
| Malignancy, except skin neoplasms | 432254 | 94144008 | Primary malignant neoplasm of Waldeyer's ring | SNOMED CT |
| Malignancy, except skin neoplasms | 40481522 | 444910004 | Primary mediastinal (thymic) large B-cell lymphoma | SNOMED CT |
| Malignancy, except skin neoplasms | 443719 | 415287001 | Relapsing chronic myeloid leukemia | SNOMED CT |
| Malignancy, except skin neoplasms | 436920 | 373168002 | Reticulosarcoma | SNOMED CT |
| Malignancy, except skin neoplasms | 200659 | 95224004 | Reticulosarcoma of intra-abdominal lymph nodes | SNOMED CT |
| Malignancy, except skin neoplasms | 192265 | 95225003 | Reticulosarcoma of intrapelvic lymph nodes | SNOMED CT |
| Malignancy, except skin neoplasms | 435193 | 95226002 | Reticulosarcoma of intrathoracic lymph nodes | SNOMED CT |
| Malignancy, except skin neoplasms | 441241 | 188492005 | Reticulosarcoma of lymph nodes of axilla and upper limb | SNOMED CT |
| Malignancy, except skin neoplasms | 438106 | 188489006 | Reticulosarcoma of lymph nodes of head, face and neck | SNOMED CT |
| Malignancy, except skin neoplasms | 200660 | 188493000 | Reticulosarcoma of lymph nodes of inguinal region and lower limb | SNOMED CT |
| Malignancy, except skin neoplasms | 139759 | 95230004 | Reticulosarcoma of lymph nodes of multiple sites | SNOMED CT |
| Malignancy, except skin neoplasms | 442128 | 95231000 | Reticulosarcoma of spleen | SNOMED CT |
| Malignancy, except skin neoplasms | 40488812 | 446643000 | Sarcoma of dendritic cells (accessory cells) | SNOMED CT |
| Malignancy, except skin neoplasms | 4115271 | 254601002 | Sarcoma of liver | SNOMED CT |
| Malignancy, except skin neoplasms | 315497 | 302855005 | Subacute leukemia | SNOMED CT |
| Malignancy, except skin neoplasms | 4143848 | 427056005 | Subacute leukemia in remission | SNOMED CT |
| Malignancy, except skin neoplasms | 136656 | 188726003 | Subacute lymphoid leukemia | SNOMED CT |
| Malignancy, except skin neoplasms | 4139054 | 426370008 | Subacute lymphoid leukemia in remission | SNOMED CT |
| Malignancy, except skin neoplasms | 313430 | 188746008 | Subacute monocytic leukemia | SNOMED CT |
| Malignancy, except skin neoplasms | 135499 | 188736006 | Subacute myeloid leukemia | SNOMED CT |
| Malignancy, except skin neoplasms | 4143382 | 425749006 | Subacute myeloid leukemia in remission | SNOMED CT |
| Malignancy, except skin neoplasms | 4041799 | 118611004 | Sézary's disease (clinical) | SNOMED CT |
| Malignancy, except skin neoplasms | 135759 | 95264000 | Sézary's disease of extranodal AND/OR solid organ site | SNOMED CT |
| Malignancy, except skin neoplasms | 439267 | 188632001 | Sézary's disease of intra-abdominal lymph nodes | SNOMED CT |
| Malignancy, except skin neoplasms | 439270 | 188635004 | Sézary's disease of intrapelvic lymph nodes | SNOMED CT |
| Malignancy, except skin neoplasms | 439265 | 188631008 | Sézary's disease of intrathoracic lymph nodes | SNOMED CT |
| Malignancy, except skin neoplasms | 138378 | 188633006 | Sézary's disease of lymph nodes of axilla AND/OR upper limb | SNOMED CT |
| Malignancy, except skin neoplasms | 135765 | 95260009 | Sézary's disease of lymph nodes of head, face AND/OR neck | SNOMED CT |
| Malignancy, except skin neoplasms | 439266 | 188634000 | Sézary's disease of lymph nodes of inguinal region and lower limb | SNOMED CT |
| Malignancy, except skin neoplasms | 199762 | 95261008 | Sézary's disease of lymph nodes of inguinal region AND/OR lower limb | SNOMED CT |
| Malignancy, except skin neoplasms | 140664 | 188637007 | Sézary's disease of lymph nodes of multiple sites | SNOMED CT |
| Malignancy, except skin neoplasms | 195760 | 95263006 | Sézary's disease of spleen | SNOMED CT |
| Malignancy, except skin neoplasms | 4079683 | 277567002 | T-cell prolymphocytic leukemia | SNOMED CT |
| Malignancy, except skin neoplasms | 4003182 | 109975001 | T-zone lymphoma (clinical) | SNOMED CT |
| Malignancy, except skin neoplasms | 4098597 | 190818004 | Waldenström macroglobulinemia | SNOMED CT |
| Renal disease | 44784439 | 698591006 | Benign hypertensive renal disease with renal failure | SNOMED CT |
| Renal disease | 312358 | 20917003 | Chronic glomerulonephritis | SNOMED CT |
| Renal disease | 442075 | 197616000 | Chronic glomerulonephritis associated with another disorder | SNOMED CT |
| Renal disease | 46271022 | 709044004 | Chronic kidney disease | SNOMED CT |
| Renal disease | 443614 | 431855005 | Chronic kidney disease stage 1 | SNOMED CT |
| Renal disease | 443601 | 431856006 | Chronic kidney disease stage 2 | SNOMED CT |
| Renal disease | 443597 | 433144002 | Chronic kidney disease stage 3 | SNOMED CT |
| Renal disease | 45763854 | 700378005 | Chronic kidney disease stage 3A | SNOMED CT |
| Renal disease | 45763855 | 700379002 | Chronic kidney disease stage 3B | SNOMED CT |
| Renal disease | 443612 | 431857002 | Chronic kidney disease stage 4 | SNOMED CT |
| Renal disease | 443611 | 433146000 | Chronic kidney disease stage 5 | SNOMED CT |
| Renal disease | 44782690 | 129161000119100 | Chronic kidney disease stage 5 due to hypertension | SNOMED CT |
| Renal disease | 4056462 | 197613008 | Chronic mesangial proliferative glomerulonephritis | SNOMED CT |
| Renal disease | 46270347 | 28191000119109 | Chronic nephritic syndrome with membranous glomerulonephritis | SNOMED CT |
| Renal disease | 4055899 | 197714009 | Chronic nephritic syndrome, dense deposit disease | SNOMED CT |
| Renal disease | 4056480 | 197715005 | Chronic nephritic syndrome, diffuse crescentic glomerulonephritis | SNOMED CT |
| Renal disease | 4059463 | 197712008 | Chronic nephritic syndrome, diffuse endocapillary proliferative glomerulonephritis | SNOMED CT |
| Renal disease | 4059584 | 197713003 | Chronic nephritic syndrome, diffuse mesangiocapillary glomerulonephritis | SNOMED CT |
| Renal disease | 45770906 | 195791000119101 | Chronic proliferative glomerulonephritis | SNOMED CT |
| Renal disease | 198185 | 90688005 | Chronic renal failure | SNOMED CT |
| Renal disease | 193782 | 46177005 | End-stage renal disease | SNOMED CT |
| Renal disease | 44784621 | 8501000119104 | Hypertensive heart and chronic kidney disease | SNOMED CT |
| Renal disease | 439694 | 194781004 | Hypertensive heart and renal disease with both (congestive) heart failure and renal failure | SNOMED CT |
| Renal disease | 439695 | 194780003 | Hypertensive heart and renal disease with renal failure | SNOMED CT |
| Renal disease | 443919 | 49220004 | Hypertensive renal failure | SNOMED CT |
| Renal disease | 198124 | 90708001 | Kidney disease | SNOMED CT |
| Renal disease | 43020455 | 285841000119104 | Malignant hypertensive end stage renal disease | SNOMED CT |
| Renal disease | 252365 | 77182004 | Membranous glomerulonephritis | SNOMED CT |
| Renal disease | 4146996 | 35546006 | Mesangial proliferative glomerulonephritis | SNOMED CT |
| Renal disease | 433257 | 80321008 | Mesangiocapillary glomerulonephritis | SNOMED CT |
| Renal disease | 4298809 | 7724006 | Nephritic syndrome | SNOMED CT |
| Renal disease | 192359 | 42399005 | Renal failure syndrome | SNOMED CT |
| Renal disease | 197921 | 16726004 | Renal osteodystrophy | SNOMED CT |
| Renal disease | 42539502 | 737295003 | Transplanted kidney present | SNOMED CT |
| Diabetes, without chronic complications | 4196141 | 314903002 | Arthropathy due to type 2 diabetes mellitus | SNOMED CT |
| Diabetes, without chronic complications | 443735 | 420662003 | Coma due to diabetes mellitus | SNOMED CT |
| Diabetes, without chronic complications | 4221933 | 420996007 | Coma due to malnutrition-related diabetes mellitus | SNOMED CT |
| Diabetes, without chronic complications | 45769832 | 72021000119109 | Dermopathy due to type 1 diabetes mellitus | SNOMED CT |
| Diabetes, without chronic complications | 43531616 | 1531000119102 | Dermopathy due to type 2 diabetes mellitus | SNOMED CT |
| Diabetes, without chronic complications | 201820 | 73211009 | Diabetes mellitus | SNOMED CT |
| Diabetes, without chronic complications | 4008576 | 111552007 | Diabetes mellitus without complication | SNOMED CT |
| Diabetes, without chronic complications | 443727 | 420422005 | Diabetic ketoacidosis | SNOMED CT |
| Diabetes, without chronic complications | 37016348 | 367991000119101 | Hyperglycemia due to type 1 diabetes mellitus | SNOMED CT |
| Diabetes, without chronic complications | 37016349 | 368051000119109 | Hyperglycemia due to type 2 diabetes mellitus | SNOMED CT |
| Diabetes, without chronic complications | 443592 | 428896009 | Hyperosmolality due to uncontrolled type 1 diabetes mellitus | SNOMED CT |
| Diabetes, without chronic complications | 4226238 | 422126006 | Hyperosmolar coma due to diabetes mellitus | SNOMED CT |
| Diabetes, without chronic complications | 201531 | 190330002 | Hyperosmolar coma due to type 1 diabetes mellitus | SNOMED CT |
| Diabetes, without chronic complications | 201530 | 190331003 | Hyperosmolar coma due to type 2 diabetes mellitus | SNOMED CT |
| Diabetes, without chronic complications | 4226798 | 421725003 | Hypoglycemic coma due to diabetes mellitus | SNOMED CT |
| Diabetes, without chronic complications | 4228112 | 421437000 | Hypoglycemic coma due to type 1 diabetes mellitus | SNOMED CT |
| Diabetes, without chronic complications | 36714116 | 719216001 | Hypoglycemic coma due to type 2 diabetes mellitus | SNOMED CT |
| Diabetes, without chronic complications | 4095288 | 26298008 | Ketoacidotic coma due to diabetes mellitus | SNOMED CT |
| Diabetes, without chronic complications | 4224254 | 421075007 | Ketoacidotic coma due to type 1 diabetes mellitus | SNOMED CT |
| Diabetes, without chronic complications | 4228443 | 421847006 | Ketoacidotic coma due to type 2 diabetes mellitus | SNOMED CT |
| Diabetes, without chronic complications | 4327944 | 75524006 | Malnutrition related diabetes mellitus | SNOMED CT |
| Diabetes, without chronic complications | 4096041 | 190406000 | Malnutrition-related diabetes mellitus with ketoacidosis | SNOMED CT |
| Diabetes, without chronic complications | 4096042 | 190412005 | Malnutrition-related diabetes mellitus without complications | SNOMED CT |
| Diabetes, without chronic complications | 201254 | 46635009 | Type 1 diabetes mellitus | SNOMED CT |
| Diabetes, without chronic complications | 4152858 | 314893005 | Type 1 diabetes mellitus with arthropathy | SNOMED CT |
| Diabetes, without chronic complications | 4099214 | 190368000 | Type 1 diabetes mellitus with ulcer | SNOMED CT |
| Diabetes, without chronic complications | 443412 | 313435000 | Type 1 diabetes mellitus without complication | SNOMED CT |
| Diabetes, without chronic complications | 201826 | 44054006 | Type 2 diabetes mellitus | SNOMED CT |
| Diabetes, without chronic complications | 4099651 | 190389009 | Type 2 diabetes mellitus with ulcer | SNOMED CT |
| Diabetes, without chronic complications | 4193704 | 313436004 | Type 2 diabetes mellitus without complication | SNOMED CT |
| Diabetes, without chronic complications | 40482801 | 443694000 | Type II diabetes mellitus uncontrolled | SNOMED CT |
| Diabetes, with chronic complications | 4175440 | 50620007 | Autonomic neuropathy due to diabetes mellitus | SNOMED CT |
| Diabetes, with chronic complications | 37016767 | 712882000 | Autonomic neuropathy due to type 1 diabetes mellitus | SNOMED CT |
| Diabetes, with chronic complications | 37016768 | 712883005 | Autonomic neuropathy due to type 2 diabetes mellitus | SNOMED CT |
| Diabetes, with chronic complications | 376979 | 43959009 | Cataract due to diabetes mellitus | SNOMED CT |
| Diabetes, with chronic complications | 4225656 | 421920002 | Cataract due to diabetes mellitus type 1 | SNOMED CT |
| Diabetes, with chronic complications | 4221495 | 420756003 | Cataract due to diabetes mellitus type 2 | SNOMED CT |
| Diabetes, with chronic complications | 43531578 | 771000119108 | Chronic kidney disease due to type 2 diabetes mellitus | SNOMED CT |
| Diabetes, with chronic complications | 442793 | 74627003 | Complication due to diabetes mellitus | SNOMED CT |
| Diabetes, with chronic complications | 4048028 | 230577008 | Diabetic mononeuropathy | SNOMED CT |
| Diabetes, with chronic complications | 435216 | 420868002 | Disorder due to type 1 diabetes mellitus | SNOMED CT |
| Diabetes, with chronic complications | 443732 | 422014003 | Disorder due to type 2 diabetes mellitus | SNOMED CT |
| Diabetes, with chronic complications | 443767 | 25093002 | Disorder of eye due to diabetes mellitus | SNOMED CT |
| Diabetes, with chronic complications | 4224419 | 421256007 | Disorder of eye due to malnutrition related diabetes mellitus | SNOMED CT |
| Diabetes, with chronic complications | 42538169 | 739681000 | Disorder of eye due to type 1 diabetes mellitus | SNOMED CT |
| Diabetes, with chronic complications | 443733 | 422099009 | Disorder of eye due to type 2 diabetes mellitus | SNOMED CT |
| Diabetes, with chronic complications | 192279 | 127013003 | Disorder of kidney due to diabetes mellitus | SNOMED CT |
| Diabetes, with chronic complications | 443730 | 422088007 | Disorder of nervous system due to diabetes mellitus | SNOMED CT |
| Diabetes, with chronic complications | 4224879 | 420683009 | Disorder of nervous system due to malnutrition related diabetes mellitus | SNOMED CT |
| Diabetes, with chronic complications | 377821 | 421468001 | Disorder of nervous system due to type 1 diabetes mellitus | SNOMED CT |
| Diabetes, with chronic complications | 376065 | 421326000 | Disorder of nervous system due to type 2 diabetes mellitus | SNOMED CT |
| Diabetes, with chronic complications | 4226354 | 422275004 | Gangrene due to diabetes mellitus | SNOMED CT |
| Diabetes, with chronic complications | 4223303 | 420825003 | Gangrene due to type 1 diabetes mellitus | SNOMED CT |
| Diabetes, with chronic complications | 4222876 | 421631007 | Gangrene due to type 2 diabetes mellitus | SNOMED CT |
| Diabetes, with chronic complications | 4191611 | 39058009 | Lumbosacral radiculoplexus neuropathy due to diabetes mellitus | SNOMED CT |
| Diabetes, with chronic complications | 4143857 | 427571000 | Lumbosacral radiculoplexus neuropathy due to type 1 diabetes mellitus | SNOMED CT |
| Diabetes, with chronic complications | 4140466 | 427027005 | Lumbosacral radiculoplexus neuropathy due to type 2 diabetes mellitus | SNOMED CT |
| Diabetes, with chronic complications | 45770830 | 97331000119101 | Macular edema and retinopathy due to type 2 diabetes mellitus | SNOMED CT |
| Diabetes, with chronic complications | 380097 | 312912001 | Macular edema due to diabetes mellitus | SNOMED CT |
| Diabetes, with chronic complications | 4096671 | 190410002 | Malnutrition-related diabetes mellitus with peripheral circulatory complications | SNOMED CT |
| Diabetes, with chronic complications | 4096670 | 190407009 | Malnutrition-related diabetes mellitus with renal complications | SNOMED CT |
| Diabetes, with chronic complications | 378743 | 312903003 | Mild nonproliferative retinopathy due to diabetes mellitus | SNOMED CT |
| Diabetes, with chronic complications | 37016179 | 138881000119106 | Mild nonproliferative retinopathy due to type 1 diabetes mellitus | SNOMED CT |
| Diabetes, with chronic complications | 45757435 | 138911000119106 | Mild nonproliferative retinopathy due to type 2 diabetes mellitus | SNOMED CT |
| Diabetes, with chronic complications | 377552 | 312904009 | Moderate nonproliferative retinopathy due to diabetes mellitus | SNOMED CT |
| Diabetes, with chronic complications | 37016180 | 138891000119109 | Moderate nonproliferative retinopathy due to type 1 diabetes mellitus | SNOMED CT |
| Diabetes, with chronic complications | 45770881 | 138921000119104 | Moderate nonproliferative retinopathy due to type 2 diabetes mellitus | SNOMED CT |
| Diabetes, with chronic complications | 4225055 | 420918009 | Mononeuropathy due to type 1 diabetes mellitus | SNOMED CT |
| Diabetes, with chronic complications | 4222415 | 420436000 | Mononeuropathy due to type 2 diabetes mellitus | SNOMED CT |
| Diabetes, with chronic complications | 40480000 | 441628001 | Multiple complications due to diabetes mellitus | SNOMED CT |
| Diabetes, with chronic complications | 4099652 | 190411003 | Multiple complications due to malnutrition related diabetes | SNOMED CT |
| Diabetes, with chronic complications | 4044391 | 230572002 | Neuropathy due to diabetes mellitus | SNOMED CT |
| Diabetes, with chronic complications | 45763583 | 60961000119107 | Nonproliferative diabetic retinopathy due to type 1 diabetes mellitus | SNOMED CT |
| Diabetes, with chronic complications | 43530656 | 1551000119108 | Nonproliferative retinopathy due to type 2 diabetes mellitus | SNOMED CT |
| Diabetes, with chronic complications | 4131908 | 127014009 | Peripheral angiopathy due to diabetes mellitus | SNOMED CT |
| Diabetes, with chronic complications | 318712 | 421365002 | Peripheral circulatory disorder due to type 1 diabetes mellitus | SNOMED CT |
| Diabetes, with chronic complications | 443729 | 422166005 | Peripheral circulatory disorder due to type 2 diabetes mellitus | SNOMED CT |
| Diabetes, with chronic complications | 321822 | 421895002 | Peripheral vascular disorder due to diabetes mellitus | SNOMED CT |
| Diabetes, with chronic complications | 376112 | 49455004 | Polyneuropathy due to diabetes mellitus | SNOMED CT |
| Diabetes, with chronic complications | 37017431 | 713705003 | Polyneuropathy due to type 1 diabetes mellitus | SNOMED CT |
| Diabetes, with chronic complications | 37017432 | 713706002 | Polyneuropathy due to type 2 diabetes mellitus | SNOMED CT |
| Diabetes, with chronic complications | 380096 | 59276001 | Proliferative retinopathy due to diabetes mellitus | SNOMED CT |
| Diabetes, with chronic complications | 45763584 | 60971000119101 | Proliferative retinopathy due to type 1 diabetes mellitus | SNOMED CT |
| Diabetes, with chronic complications | 43530685 | 1501000119109 | Proliferative retinopathy due to type 2 diabetes mellitus | SNOMED CT |
| Diabetes, with chronic complications | 200687 | 421893009 | Renal disorder due to type 1 diabetes mellitus | SNOMED CT |
| Diabetes, with chronic complications | 443731 | 420279001 | Renal disorder due to type 2 diabetes mellitus | SNOMED CT |
| Diabetes, with chronic complications | 4174977 | 4855003 | Retinopathy due to diabetes mellitus | SNOMED CT |
| Diabetes, with chronic complications | 4227210 | 420789003 | Retinopathy due to type 1 diabetes mellitus | SNOMED CT |
| Diabetes, with chronic complications | 376114 | 312905005 | Severe nonproliferative retinopathy due to diabetes mellitus | SNOMED CT |
| Diabetes, with chronic complications | 4290822 | 399872003 | Severe nonproliferative retinopathy with clinically significant macular edema due to diabetes mellitus | SNOMED CT |
| Diabetes, with chronic complications | 4266637 | 399873008 | Severe nonproliferative retinopathy without macular edema due to diabetes mellitus | SNOMED CT |
| Diabetes, with chronic complications | 4338901 | 232023006 | Traction detachment of retina due to diabetes mellitus | SNOMED CT |
| Diabetes, with chronic complications | 45769873 | 82571000119107 | Traction detachment of retina due to type 1 diabetes mellitus | SNOMED CT |
| Diabetes, with chronic complications | 45773064 | 82541000119100 | Traction detachment of retina due to type 2 diabetes mellitus | SNOMED CT |
| Cerebrovascular disease | 4164092 | 29322000 | Acute cerebrovascular insufficiency | SNOMED CT |
| Cerebrovascular disease | 374060 | 288723005 | Acute ill-defined cerebrovascular disease | SNOMED CT |
| Cerebrovascular disease | 42535426 | 291581000119109 | Acute nontraumatic subdural hemorrhage | SNOMED CT |
| Cerebrovascular disease | 4338523 | 88032003 | Amaurosis fugax | SNOMED CT |
| Cerebrovascular disease | 4108360 | 195210002 | Anterior cerebral artery syndrome | SNOMED CT |
| Cerebrovascular disease | 40480002 | 441630004 | Aphasia as late effect of cerebrovascular disease | SNOMED CT |
| Cerebrovascular disease | 43530679 | 145741000119101 | Apraxia as late effect of cerebrovascular disease | SNOMED CT |
| Cerebrovascular disease | 443551 | 428668000 | Apraxia due to cerebrovascular accident | SNOMED CT |
| Cerebrovascular disease | 43531622 | 29941000119105 | Ataxia as sequela of cerebrovascular disease | SNOMED CT |
| Cerebrovascular disease | 4006294 | 111296006 | Basilar artery embolism | SNOMED CT |
| Cerebrovascular disease | 374055 | 64009001 | Basilar artery syndrome | SNOMED CT |
| Cerebrovascular disease | 4338227 | 88174006 | Basilar artery thrombosis | SNOMED CT |
| Cerebrovascular disease | 759831 | 106241000119108 | Bilateral paralytic syndrome as late effect of stroke | SNOMED CT |
| Cerebrovascular disease | 4319328 | 95454007 | Brain stem hemorrhage | SNOMED CT |
| Cerebrovascular disease | 4111710 | 195212005 | Brainstem stroke syndrome | SNOMED CT |
| Cerebrovascular disease | 4213731 | 80606009 | Carotid artery embolism | SNOMED CT |
| Cerebrovascular disease | 4288310 | 69798007 | Carotid artery obstruction | SNOMED CT |
| Cerebrovascular disease | 313226 | 266254007 | Carotid artery occlusion | SNOMED CT |
| Cerebrovascular disease | 4112020 | 195200006 | Carotid artery syndrome hemispheric | SNOMED CT |
| Cerebrovascular disease | 4311124 | 86003009 | Carotid artery thrombosis | SNOMED CT |
| Cerebrovascular disease | 4111711 | 195213000 | Cerebellar stroke syndrome | SNOMED CT |
| Cerebrovascular disease | 4045749 | 230724001 | Cerebral amyloid angiopathy | SNOMED CT |
| Cerebrovascular disease | 380747 | 28366008 | Cerebral arteritis | SNOMED CT |
| Cerebrovascular disease | 45766199 | 703311009 | Cerebral arteritis due to infectious disease | SNOMED CT |
| Cerebrovascular disease | 372924 | 20059004 | Cerebral artery occlusion | SNOMED CT |
| Cerebrovascular disease | 316437 | 55382008 | Cerebral atherosclerosis | SNOMED CT |
| Cerebrovascular disease | 4190891 | 390936003 | Cerebral autosomal dominant arteriopathy with subcortical infarcts and leukoencephalopathy | SNOMED CT |
| Cerebrovascular disease | 375557 | 75543006 | Cerebral embolism | SNOMED CT |
| Cerebrovascular disease | 376713 | 274100004 | Cerebral hemorrhage | SNOMED CT |
| Cerebrovascular disease | 4110189 | 195185009 | Cerebral infarct due to thrombosis of precerebral arteries | SNOMED CT |
| Cerebrovascular disease | 443454 | 432504007 | Cerebral infarction | SNOMED CT |
| Cerebrovascular disease | 4111714 | 195230003 | Cerebral infarction due to cerebral venous thrombosis, non-pyogenic | SNOMED CT |
| Cerebrovascular disease | 4108356 | 195190007 | Cerebral infarction due to embolism of cerebral arteries | SNOMED CT |
| Cerebrovascular disease | 45772786 | 705128004 | Cerebral infarction due to embolism of middle cerebral artery | SNOMED CT |
| Cerebrovascular disease | 4110190 | 195186005 | Cerebral infarction due to embolism of precerebral arteries | SNOMED CT |
| Cerebrovascular disease | 46273649 | 34181000119102 | Cerebral infarction due to occlusion of basilar artery | SNOMED CT |
| Cerebrovascular disease | 46270031 | 125081000119106 | Cerebral infarction due to occlusion of precerebral artery | SNOMED CT |
| Cerebrovascular disease | 4110192 | 195189003 | Cerebral infarction due to thrombosis of cerebral arteries | SNOMED CT |
| Cerebrovascular disease | 45767658 | 705130002 | Cerebral infarction due to thrombosis of middle cerebral artery | SNOMED CT |
| Cerebrovascular disease | 374384 | 287731003 | Cerebral ischemia | SNOMED CT |
| Cerebrovascular disease | 441874 | 71444005 | Cerebral thrombosis | SNOMED CT |
| Cerebrovascular disease | 45766121 | 703218000 | Cerebral vasoconstriction syndrome | SNOMED CT |
| Cerebrovascular disease | 381316 | 230690007 | Cerebrovascular accident | SNOMED CT |
| Cerebrovascular disease | 381591 | 62914000 | Cerebrovascular disease | SNOMED CT |
| Cerebrovascular disease | 43530851 | 609382000 | Chronic non-traumatic intracranial subdural hemorrhage | SNOMED CT |
| Cerebrovascular disease | 4176892 | 49422009 | Cortical hemorrhage | SNOMED CT |
| Cerebrovascular disease | 4121341 | 302909007 | Diffuse cerebrovascular disease | SNOMED CT |
| Cerebrovascular disease | 43022059 | 473449006 | Disease of non-coronary systemic artery | SNOMED CT |
| Cerebrovascular disease | 4159164 | 371158002 | Disorder of basilar artery | SNOMED CT |
| Cerebrovascular disease | 4153380 | 371160000 | Disorder of carotid artery | SNOMED CT |
| Cerebrovascular disease | 37016924 | 713081000 | Dissection of cerebral artery | SNOMED CT |
| Cerebrovascular disease | 43530687 | 133981000119106 | Dysarthria as late effects of cerebrovascular disease | SNOMED CT |
| Cerebrovascular disease | 443465 | 426033005 | Dysphagia as a late effect of cerebrovascular accident | SNOMED CT |
| Cerebrovascular disease | 40479575 | 441529001 | Dysphasia as late effect of cerebrovascular disease | SNOMED CT |
| Cerebrovascular disease | 43530688 | 133991000119109 | Fluency disorder as sequela of cerebrovascular disease | SNOMED CT |
| Cerebrovascular disease | 44782781 | 48601000119107 | Hemiplegia and/or hemiparesis following stroke | SNOMED CT |
| Cerebrovascular disease | 40481762 | 442024001 | Hemiplegia as late effect of cerebrovascular disease | SNOMED CT |
| Cerebrovascular disease | 40484522 | 442676003 | Hemiplegia of dominant side as late effect of cerebrovascular disease | SNOMED CT |
| Cerebrovascular disease | 40484513 | 442668000 | Hemiplegia of nondominant side as late effect of cerebrovascular disease | SNOMED CT |
| Cerebrovascular disease | 443916 | 32895009 | Hereditary disease | SNOMED CT |
| Cerebrovascular disease | 4180158 | 363137000 | Hereditary disorder by system | SNOMED CT |
| Cerebrovascular disease | 312938 | 50490005 | Hypertensive encephalopathy | SNOMED CT |
| Cerebrovascular disease | 4043731 | 230692004 | Infarction - precerebral | SNOMED CT |
| Cerebrovascular disease | 4110185 | 195168007 | Intracerebral hemorrhage, intraventricular | SNOMED CT |
| Cerebrovascular disease | 4110186 | 195169004 | Intracerebral hemorrhage, multiple localized | SNOMED CT |
| Cerebrovascular disease | 4353709 | 250845006 | Intracerebral vascular finding | SNOMED CT |
| Cerebrovascular disease | 439847 | 1386000 | Intracranial hemorrhage | SNOMED CT |
| Cerebrovascular disease | 4179912 | 297157005 | Intracranial venous thrombosis | SNOMED CT |
| Cerebrovascular disease | 4046360 | 230698000 | Lacunar infarction | SNOMED CT |
| Cerebrovascular disease | 434056 | 195239002 | Late effects of cerebrovascular disease | SNOMED CT |
| Cerebrovascular disease | 4027461 | 22811006 | Leukoencephalopathy | SNOMED CT |
| Cerebrovascular disease | 4110194 | 195209007 | Middle cerebral artery syndrome | SNOMED CT |
| Cerebrovascular disease | 197303 | 425642008 | Monoplegia of dominant lower limb as a late effect of cerebrovascular accident | SNOMED CT |
| Cerebrovascular disease | 443525 | 427065003 | Monoplegia of dominant upper limb as a late effect of cerebrovascular accident | SNOMED CT |
| Cerebrovascular disease | 40480938 | 441887006 | Monoplegia of lower limb as late effect of cerebrovascular disease | SNOMED CT |
| Cerebrovascular disease | 40480946 | 441894009 | Monoplegia of nondominant lower limb as a late effect of cerebrovascular accident | SNOMED CT |
| Cerebrovascular disease | 40482266 | 442181008 | Monoplegia of nondominant upper limb as a late effect of cerebrovascular accident | SNOMED CT |
| Cerebrovascular disease | 40481842 | 442097001 | Monoplegia of upper limb as late effect of cerebrovascular disease | SNOMED CT |
| Cerebrovascular disease | 378774 | 69116000 | Moyamoya disease | SNOMED CT |
| Cerebrovascular disease | 381036 | 73192008 | Multiple AND bilateral precerebral artery stenosis | SNOMED CT |
| Cerebrovascular disease | 4144154 | 425957003 | Non-traumatic intracerebral ventricular hemorrhage | SNOMED CT |
| Cerebrovascular disease | 4111709 | 195176009 | Non-traumatic subdural hemorrhage | SNOMED CT |
| Cerebrovascular disease | 314667 | 42970005 | Nonpyogenic thrombosis of intracranial venous sinus | SNOMED CT |
| Cerebrovascular disease | 436430 | 397809001 | Nontraumatic extradural hemorrhage | SNOMED CT |
| Cerebrovascular disease | 4111716 | 195233001 | Occlusion and stenosis of anterior cerebral artery | SNOMED CT |
| Cerebrovascular disease | 4112024 | 195235008 | Occlusion and stenosis of cerebellar arteries | SNOMED CT |
| Cerebrovascular disease | 4112023 | 195232006 | Occlusion and stenosis of middle cerebral artery | SNOMED CT |
| Cerebrovascular disease | 4111717 | 195234007 | Occlusion and stenosis of posterior cerebral artery | SNOMED CT |
| Cerebrovascular disease | 372654 | 425882004 | Paralytic syndrome as late effect of stroke | SNOMED CT |
| Cerebrovascular disease | 443609 | 430959006 | Paralytic syndrome of dominant side as late effect of stroke | SNOMED CT |
| Cerebrovascular disease | 443599 | 430947007 | Paralytic syndrome of nondominant side as late effect of stroke | SNOMED CT |
| Cerebrovascular disease | 43530742 | 361000119103 | Paralytic syndrome on one side of the body as late effect of cerebrovascular accident | SNOMED CT |
| Cerebrovascular disease | 4110195 | 195211003 | Posterior cerebral artery syndrome | SNOMED CT |
| Cerebrovascular disease | 42872891 | 450886002 | Posterior reversible encephalopathy syndrome | SNOMED CT |
| Cerebrovascular disease | 443239 | 266253001 | Precerebral arterial occlusion | SNOMED CT |
| Cerebrovascular disease | 4045737 | 230699008 | Pure motor lacunar infarction | SNOMED CT |
| Cerebrovascular disease | 4045738 | 230700009 | Pure sensory lacunar infarction | SNOMED CT |
| Cerebrovascular disease | 40482301 | 442212003 | Residual cognitive deficit as late effect of cerebrovascular accident | SNOMED CT |
| Cerebrovascular disease | 45773220 | 700467001 | Reversible cerebral vasoconstriction syndrome | SNOMED CT |
| Cerebrovascular disease | 40480449 | 441735003 | Sensory disorder as a late effect of cerebrovascular disease | SNOMED CT |
| Cerebrovascular disease | 43530623 | 1131000119105 | Sequela of cerebrovascular accident | SNOMED CT |
| Cerebrovascular disease | 4112026 | 195243003 | Sequelae of cerebral infarction | SNOMED CT |
| Cerebrovascular disease | 4111721 | 195241001 | Sequelae of intracerebral hemorrhage | SNOMED CT |
| Cerebrovascular disease | 4111720 | 195240000 | Sequelae of subarachnoid hemorrhage | SNOMED CT |
| Cerebrovascular disease | 40481354 | 441960006 | Speech and language deficit as late effect of cerebrovascular accident | SNOMED CT |
| Cerebrovascular disease | 43530674 | 142851000119103 | Spontaneous cerebellar hemorrhage | SNOMED CT |
| Cerebrovascular disease | 43530727 | 291571000119106 | Spontaneous cerebral hemorrhage | SNOMED CT |
| Cerebrovascular disease | 42539269 | 291541000119104 | Spontaneous hemorrhage of brain stem | SNOMED CT |
| Cerebrovascular disease | 42535425 | 291531000119108 | Spontaneous hemorrhage of cerebral hemisphere | SNOMED CT |
| Cerebrovascular disease | 42535424 | 291521000119105 | Spontaneous hemorrhage of cortical intracerebral hemisphere | SNOMED CT |
| Cerebrovascular disease | 42538062 | 738779002 | Spontaneous intracranial hemorrhage | SNOMED CT |
| Cerebrovascular disease | 4148906 | 270907008 | Spontaneous subarachnoid hemorrhage | SNOMED CT |
| Cerebrovascular disease | 432923 | 21454007 | Subarachnoid hemorrhage | SNOMED CT |
| Cerebrovascular disease | 4108952 | 195155004 | Subarachnoid hemorrhage from carotid siphon and bifurcation | SNOMED CT |
| Cerebrovascular disease | 4111708 | 195160000 | Subarachnoid hemorrhage from vertebral artery | SNOMED CT |
| Cerebrovascular disease | 433505 | 15258001 | Subclavian steal syndrome | SNOMED CT |
| Cerebrovascular disease | 4049659 | 20908003 | Subcortical hemorrhage | SNOMED CT |
| Cerebrovascular disease | 439040 | 35486000 | Subdural hemorrhage | SNOMED CT |
| Cerebrovascular disease | 433195 | 87224000 | Transient arterial retinal occlusion | SNOMED CT |
| Cerebrovascular disease | 373503 | 266257000 | Transient cerebral ischemia | SNOMED CT |
| Cerebrovascular disease | 437306 | 230736007 | Transient global amnesia | SNOMED CT |
| Cerebrovascular disease | 4274969 | 65084004 | Vertebral artery embolism | SNOMED CT |
| Cerebrovascular disease | 434656 | 34781003 | Vertebral artery syndrome | SNOMED CT |
| Cerebrovascular disease | 4273526 | 64775002 | Vertebral artery thrombosis | SNOMED CT |
| Cerebrovascular disease | 376714 | 195199008 | Vertebrobasilar artery syndrome | SNOMED CT |
| Cerebrovascular disease | 43531621 | 26021000119107 | Vertigo as sequela of cerebrovascular disease | SNOMED CT |
| Cerebrovascular disease | 43531583 | 87551000119101 | Visual disturbance as sequela of cerebrovascular disease | SNOMED CT |
| Cerebrovascular disease | 44782753 | 148871000119109 | Weakness as a late effect of stroke | SNOMED CT |
| Cerebrovascular disease | 43530744 | 40161000119102 | Weakness of face muscles as sequela of stroke | SNOMED CT |
| AIDS/HIV | 37017595 | 713897006 | Burkitt lymphoma co-occurrent with human immunodeficiency virus infection | SNOMED CT |
| AIDS/HIV | 37017319 | 713571008 | Disorder of central nervous system co-occurrent with human immunodeficiency virus infection | SNOMED CT |
| AIDS/HIV | 37017244 | 713484001 | Disorder of respiratory system co-occurrent with human immunodeficiency virus infection | SNOMED CT |
| AIDS/HIV | 4262297 | 397763006 | Human immunodeficiency virus encephalopathy | SNOMED CT |
| AIDS/HIV | 439727 | 86406008 | Human immunodeficiency virus infection | SNOMED CT |
| AIDS/HIV | 4092686 | 186708007 | Human immunodeficiency virus infection with secondary clinical infectious disease | SNOMED CT |
| AIDS/HIV | 37017446 | 713722001 | Infection caused by Cytomegalovirus co-occurrent with human immunodeficiency virus infection | SNOMED CT |
| AIDS/HIV | 37017248 | 713490002 | Infection caused by Pneumocystis co-occurrent with human immunodeficiency virus infection | SNOMED CT |
| AIDS/HIV | 37017320 | 713572001 | Malignant neoplastic disease co-occurrent with human immunodeficiency virus infection | SNOMED CT |
| AIDS/HIV | 40484012 | 442537007 | Non-Hodgkin lymphoma associated with Human immunodeficiency virus infection | SNOMED CT |
| AIDS/HIV | 37017579 | 713880000 | Opportunistic mycosis co-occurrent with human immunodeficiency virus infection | SNOMED CT |
| Chronic pulmonary disease | 45771045 | 708038006 | Acute exacerbation of asthma | SNOMED CT |
| Chronic pulmonary disease | 40483342 | 445378003 | Acute exacerbation of bronchiectasis | SNOMED CT |
| Chronic pulmonary disease | 257004 | 195951007 | Acute exacerbation of chronic obstructive airways disease | SNOMED CT |
| Chronic pulmonary disease | 43530693 | 1751000119100 | Acute exacerbation of chronic obstructive airways disease with asthma | SNOMED CT |
| Chronic pulmonary disease | 46270376 | 293241000119100 | Acute exacerbation of chronic obstructive bronchitis | SNOMED CT |
| Chronic pulmonary disease | 45773005 | 708094006 | Acute exacerbation of intrinsic asthma | SNOMED CT |
| Chronic pulmonary disease | 46270082 | 135181000119109 | Acute exacerbation of mild persistent asthma | SNOMED CT |
| Chronic pulmonary disease | 46273487 | 135171000119106 | Acute exacerbation of moderate persistent asthma | SNOMED CT |
| Chronic pulmonary disease | 45769438 | 708090002 | Acute severe exacerbation of asthma | SNOMED CT |
| Chronic pulmonary disease | 45769442 | 708095007 | Acute severe exacerbation of immunoglobin E-mediated allergic asthma | SNOMED CT |
| Chronic pulmonary disease | 45769443 | 708096008 | Acute severe exacerbation of intrinsic asthma | SNOMED CT |
| Chronic pulmonary disease | 45769352 | 707981009 | Acute severe exacerbation of mild persistent asthma | SNOMED CT |
| Chronic pulmonary disease | 45769351 | 707980005 | Acute severe exacerbation of moderate persistent asthma | SNOMED CT |
| Chronic pulmonary disease | 45769350 | 707979007 | Acute severe exacerbation of severe persistent asthma | SNOMED CT |
| Chronic pulmonary disease | 37116845 | 733858005 | Acute severe refractory exacerbation of asthma | SNOMED CT |
| Chronic pulmonary disease | 4191479 | 389145006 | Allergic asthma | SNOMED CT |
| Chronic pulmonary disease | 4236624 | 90623003 | Aluminosis of lung | SNOMED CT |
| Chronic pulmonary disease | 256450 | 22607003 | Asbestosis | SNOMED CT |
| Chronic pulmonary disease | 46269801 | 10692681000119100 | Aspirin exacerbated respiratory disease | SNOMED CT |
| Chronic pulmonary disease | 4250128 | 407674008 | Aspirin-induced asthma | SNOMED CT |
| Chronic pulmonary disease | 317009 | 195967001 | Asthma | SNOMED CT |
| Chronic pulmonary disease | 4148124 | 266356006 | Atrophic (senile) emphysema | SNOMED CT |
| Chronic pulmonary disease | 437588 | 67242002 | Bagassosis | SNOMED CT |
| Chronic pulmonary disease | 4032314 | 14700006 | Bauxite fibrosis of lung | SNOMED CT |
| Chronic pulmonary disease | 4221139 | 8247009 | Berylliosis | SNOMED CT |
| Chronic pulmonary disease | 439853 | 69339004 | Bird-fanciers' lung | SNOMED CT |
| Chronic pulmonary disease | 256449 | 12295008 | Bronchiectasis | SNOMED CT |
| Chronic pulmonary disease | 256451 | 32398004 | Bronchitis | SNOMED CT |
| Chronic pulmonary disease | 4133623 | 278976004 | Bulla of lung | SNOMED CT |
| Chronic pulmonary disease | 4311814 | 85761009 | Byssinosis | SNOMED CT |
| Chronic pulmonary disease | 4302900 | 78723001 | Cannabinosis | SNOMED CT |
| Chronic pulmonary disease | 4286497 | 68328006 | Centriacinar emphysema | SNOMED CT |
| Chronic pulmonary disease | 4051466 | 233678006 | Childhood asthma | SNOMED CT |
| Chronic pulmonary disease | 256448 | 195949008 | Chronic asthmatic bronchitis | SNOMED CT |
| Chronic pulmonary disease | 42539089 | 737180005 | Chronic bronchiolitis | SNOMED CT |
| Chronic pulmonary disease | 255841 | 63480004 | Chronic bronchitis | SNOMED CT |
| Chronic pulmonary disease | 4110048 | 195957006 | Chronic bullous emphysema | SNOMED CT |
| Chronic pulmonary disease | 4195892 | 79955004 | Chronic cor pulmonale | SNOMED CT |
| Chronic pulmonary disease | 4112814 | 196053000 | Chronic drug-induced interstitial lung disorders | SNOMED CT |
| Chronic pulmonary disease | 255573 | 13645005 | Chronic obstructive lung disease | SNOMED CT |
| Chronic pulmonary disease | 4110056 | 196001008 | Chronic obstructive pulmonary disease with acute lower respiratory infection | SNOMED CT |
| Chronic pulmonary disease | 44782732 | 133971000119108 | Chronic pulmonary embolism | SNOMED CT |
| Chronic pulmonary disease | 315831 | 87837008 | Chronic pulmonary heart disease | SNOMED CT |
| Chronic pulmonary disease | 252348 | 196049002 | Chronic pulmonary radiation disease | SNOMED CT |
| Chronic pulmonary disease | 3655113 | 846635004 | Chronic respiratory condition caused by vapors | SNOMED CT |
| Chronic pulmonary disease | 252946 | 29422001 | Coal workers' pneumoconiosis | SNOMED CT |
| Chronic pulmonary disease | 313236 | 409663006 | Cough variant asthma | SNOMED CT |
| Chronic pulmonary disease | 4328679 | 430476004 | Diffuse panbronchiolitis | SNOMED CT |
| Chronic pulmonary disease | 40493243 | 445928005 | Eisenmenger's syndrome | SNOMED CT |
| Chronic pulmonary disease | 259043 | 57686001 | Emphysematous bleb of lung | SNOMED CT |
| Chronic pulmonary disease | 258780 | 185086009 | Emphysematous bronchitis | SNOMED CT |
| Chronic pulmonary disease | 4138760 | 425969006 | Exacerbation of intermittent asthma | SNOMED CT |
| Chronic pulmonary disease | 45768908 | 707440005 | Exercise induced bronchospasm | SNOMED CT |
| Chronic pulmonary disease | 444084 | 37471005 | Extrinsic allergic alveolitis | SNOMED CT |
| Chronic pulmonary disease | 435298 | 18690003 | Farmers' lung | SNOMED CT |
| Chronic pulmonary disease | 4322799 | 71193007 | Fibrosis of lung caused by radiation | SNOMED CT |
| Chronic pulmonary disease | 4027669 | 13151001 | Flax-dressers' disease | SNOMED CT |
| Chronic pulmonary disease | 4066407 | 17385007 | Graphite fibrosis of lung | SNOMED CT |
| Chronic pulmonary disease | 434670 | 48347002 | Humidifier lung | SNOMED CT |
| Chronic pulmonary disease | 312950 | 424643009 | IgE-mediated allergic asthma | SNOMED CT |
| Chronic pulmonary disease | 4145497 | 266361008 | Intrinsic asthma | SNOMED CT |
| Chronic pulmonary disease | 4119298 | 233679003 | Late onset asthma | SNOMED CT |
| Chronic pulmonary disease | 434975 | 25897000 | Malt-workers' lung | SNOMED CT |
| Chronic pulmonary disease | 438175 | 86638007 | Maple-bark strippers' lung | SNOMED CT |
| Chronic pulmonary disease | 4146581 | 427679007 | Mild intermittent asthma | SNOMED CT |
| Chronic pulmonary disease | 4143828 | 426979002 | Mild persistent asthma | SNOMED CT |
| Chronic pulmonary disease | 4110051 | 195977004 | Mixed asthma | SNOMED CT |
| Chronic pulmonary disease | 4112826 | 195953005 | Mixed simple and mucopurulent chronic bronchitis | SNOMED CT |
| Chronic pulmonary disease | 4142738 | 427295004 | Moderate persistent asthma | SNOMED CT |
| Chronic pulmonary disease | 257905 | 74417001 | Mucopurulent chronic bronchitis | SNOMED CT |
| Chronic pulmonary disease | 433233 | 52333004 | Mushroom workers' lung | SNOMED CT |
| Chronic pulmonary disease | 4177944 | 4981000 | Panacinar emphysema | SNOMED CT |
| Chronic pulmonary disease | 259044 | 40122008 | Pneumoconiosis | SNOMED CT |
| Chronic pulmonary disease | 4112676 | 196017002 | Pneumoconiosis associated with tuberculosis | SNOMED CT |
| Chronic pulmonary disease | 254389 | 17996008 | Pneumoconiosis due to inorganic dust | SNOMED CT |
| Chronic pulmonary disease | 442125 | 805002 | Pneumoconiosis due to silica | SNOMED CT |
| Chronic pulmonary disease | 256146 | 426853005 | Pneumoconiosis due to silicate | SNOMED CT |
| Chronic pulmonary disease | 4249010 | 73144008 | Pneumoconiosis due to talc | SNOMED CT |
| Chronic pulmonary disease | 258781 | 74015002 | Pneumonopathy due to inhalation of dust | SNOMED CT |
| Chronic pulmonary disease | 261325 | 87433001 | Pulmonary emphysema | SNOMED CT |
| Chronic pulmonary disease | 4167085 | 274096000 | Pulmonary heart disease | SNOMED CT |
| Chronic pulmonary disease | 4167085 | 274096000 | Pulmonary heart disease | SNOMED CT |
| Chronic pulmonary disease | 4266525 | 62371005 | Pulmonary siderosis | SNOMED CT |
| Chronic pulmonary disease | 4117865 | 286964001 | Radiation respiratory disease | SNOMED CT |
| Chronic pulmonary disease | 4152913 | 370221004 | Severe asthma | SNOMED CT |
| Chronic pulmonary disease | 4145356 | 426656000 | Severe persistent asthma | SNOMED CT |
| Chronic pulmonary disease | 261889 | 61937009 | Simple chronic bronchitis | SNOMED CT |
| Chronic pulmonary disease | 4196950 | 51277007 | Stannosis | SNOMED CT |
| Chronic pulmonary disease | 443890 | 13394002 | Suberosis | SNOMED CT |
| Chronic pulmonary disease | 45768910 | 707444001 | Uncomplicated asthma | SNOMED CT |
| Chronic pulmonary disease | 45768963 | 707511009 | Uncomplicated mild persistent asthma | SNOMED CT |
| Chronic pulmonary disease | 45768964 | 707512002 | Uncomplicated moderate persistent asthma | SNOMED CT |
| Chronic pulmonary disease | 45768965 | 707513007 | Uncomplicated severe persistent asthma | SNOMED CT |
| Metastatic solid tumor | 4160276 | 430556008 | Malignant neoplasm of genital structure | SNOMED CT |
| Metastatic solid tumor | 443392 | 363346000 | Malignant neoplastic disease | SNOMED CT |
| Metastatic solid tumor | 4131422 | 127250009 | Neoplasm of intra-abdominal lymph nodes | SNOMED CT |
| Metastatic solid tumor | 4131428 | 127267002 | Neoplasm of intrapelvic lymph node | SNOMED CT |
| Metastatic solid tumor | 4131304 | 127245003 | Neoplasm of intrathoracic lymph nodes | SNOMED CT |
| Metastatic solid tumor | 4130839 | 127232002 | Neoplasm of lymph node | SNOMED CT |
| Metastatic solid tumor | 4131938 | 127261001 | Neoplasm of lymph node of lower limb | SNOMED CT |
| Metastatic solid tumor | 4133007 | 127274007 | Neoplasm of lymph nodes of multiple sites | SNOMED CT |
| Metastatic solid tumor | 4130842 | 127254000 | Neoplasm of lymph nodes of upper limb | SNOMED CT |
| Metastatic solid tumor | 443252 | 254289008 | Post-transplant neoplasia | SNOMED CT |
| Metastatic solid tumor | 439392 | 372087000 | Primary malignant neoplasm | SNOMED CT |
| Metastatic solid tumor | 193144 | 94161006 | Secondary malignant neoplasm of adrenal gland | SNOMED CT |
| Metastatic solid tumor | 4312802 | 94186002 | Secondary malignant neoplasm of bladder | SNOMED CT |
| Metastatic solid tumor | 78097 | 94222008 | Secondary malignant neoplasm of bone | SNOMED CT |
| Metastatic solid tumor | 4246450 | 94217008 | Secondary malignant neoplasm of bone marrow | SNOMED CT |
| Metastatic solid tumor | 4246451 | 94225005 | Secondary malignant neoplasm of brain | SNOMED CT |
| Metastatic solid tumor | 378087 | 188462001 | Secondary malignant neoplasm of brain and spinal cord | SNOMED CT |
| Metastatic solid tumor | 4312290 | 94246001 | Secondary malignant neoplasm of cerebral meninges | SNOMED CT |
| Metastatic solid tumor | 140960 | 94297009 | Secondary malignant neoplasm of female breast | SNOMED CT |
| Metastatic solid tumor | 4247962 | 94313005 | Secondary malignant neoplasm of gastrointestinal tract | SNOMED CT |
| Metastatic solid tumor | 192568 | 94347008 | Secondary malignant neoplasm of intra-abdominal lymph nodes | SNOMED CT |
| Metastatic solid tumor | 4312023 | 94348003 | Secondary malignant neoplasm of intra-abdominal organs | SNOMED CT |
| Metastatic solid tumor | 200959 | 94350006 | Secondary malignant neoplasm of intrapelvic lymph nodes | SNOMED CT |
| Metastatic solid tumor | 439751 | 94351005 | Secondary malignant neoplasm of intrathoracic lymph nodes | SNOMED CT |
| Metastatic solid tumor | 196053 | 94360002 | Secondary malignant neoplasm of kidney | SNOMED CT |
| Metastatic solid tumor | 200348 | 94365007 | Secondary malignant neoplasm of large intestine | SNOMED CT |
| Metastatic solid tumor | 46273652 | 353741000119106 | Secondary malignant neoplasm of left lung | SNOMED CT |
| Metastatic solid tumor | 4281027 | 369523007 | Secondary malignant neoplasm of left ovary | SNOMED CT |
| Metastatic solid tumor | 198700 | 94381002 | Secondary malignant neoplasm of liver | SNOMED CT |
| Metastatic solid tumor | 44806773 | 813671000000107 | Secondary malignant neoplasm of liver and intrahepatic bile duct | SNOMED CT |
| Metastatic solid tumor | 254591 | 94391008 | Secondary malignant neoplasm of lung | SNOMED CT |
| Metastatic solid tumor | 318096 | 94392001 | Secondary malignant neoplasm of lymph node | SNOMED CT |
| Metastatic solid tumor | 442182 | 94395004 | Secondary malignant neoplasm of lymph nodes of lower limb | SNOMED CT |
| Metastatic solid tumor | 320342 | 94396003 | Secondary malignant neoplasm of lymph nodes of multiple sites | SNOMED CT |
| Metastatic solid tumor | 434298 | 94398002 | Secondary malignant neoplasm of lymph nodes of upper limb | SNOMED CT |
| Metastatic solid tumor | 434875 | 94409002 | Secondary malignant neoplasm of mediastinum | SNOMED CT |
| Metastatic solid tumor | 373425 | 94442001 | Secondary malignant neoplasm of nervous system | SNOMED CT |
| Metastatic solid tumor | 199752 | 94455000 | Secondary malignant neoplasm of ovary | SNOMED CT |
| Metastatic solid tumor | 72266 | 94493005 | Secondary malignant neoplasm of pleura | SNOMED CT |
| Metastatic solid tumor | 4147162 | 269473008 | Secondary malignant neoplasm of respiratory and digestive systems | SNOMED CT |
| Metastatic solid tumor | 253717 | 94515004 | Secondary malignant neoplasm of respiratory tract | SNOMED CT |
| Metastatic solid tumor | 4315806 | 94628003 | Secondary malignant neoplasm of retroperitoneum | SNOMED CT |
| Metastatic solid tumor | 196925 | 188445006 | Secondary malignant neoplasm of retroperitoneum and peritoneum | SNOMED CT |
| Metastatic solid tumor | 46270513 | 353561000119103 | Secondary malignant neoplasm of right lung | SNOMED CT |
| Metastatic solid tumor | 4281030 | 369530001 | Secondary malignant neoplasm of right ovary | SNOMED CT |
| Metastatic solid tumor | 136354 | 94579000 | Secondary malignant neoplasm of skin | SNOMED CT |
| Metastatic solid tumor | 198371 | 94580002 | Secondary malignant neoplasm of small intestine | SNOMED CT |
| Metastatic solid tumor | 4314071 | 94649002 | Secondary malignant neoplasm of trunk | SNOMED CT |
| Metastatic solid tumor | 4158910 | 274088005 | Secondary malignant neoplasm of unknown site | SNOMED CT |
| Metastatic solid tumor | 78987 | 94663008 | Secondary malignant neoplasm of urinary system | SNOMED CT |
| Metastatic solid tumor | 432851 | 128462008 | Secondary malignant neoplastic disease | SNOMED CT |
| Moderate or severe liver disease | 4340386 | 235881000 | Alcoholic hepatic failure | SNOMED CT |
| Moderate or severe liver disease | 28779 | 17709002 | Bleeding esophageal varices | SNOMED CT |
| Moderate or severe liver disease | 4340390 | 235886005 | Chronic hepatic failure | SNOMED CT |
| Moderate or severe liver disease | 24966 | 28670008 | Esophageal varices | SNOMED CT |
| Moderate or severe liver disease | 4111998 | 195474004 | Esophageal varices associated with another disorder | SNOMED CT |
| Moderate or severe liver disease | 4112183 | 195475003 | Esophageal varices with bleeding, associated with another disorder | SNOMED CT |
| Moderate or severe liver disease | 22340 | 14223005 | Esophageal varices without bleeding | SNOMED CT |
| Moderate or severe liver disease | 4237824 | 91109007 | Gastric varices | SNOMED CT |
| Moderate or severe liver disease | 46269818 | 1082621000119100 | Hepatic coma due to alcoholic liver failure | SNOMED CT |
| Moderate or severe liver disease | 46269836 | 1085091000119100 | Hepatic coma due to chronic hepatic failure | SNOMED CT |
| Moderate or severe liver disease | 4029488 | 13920009 | Hepatic encephalopathy | SNOMED CT |
| Moderate or severe liver disease | 4245975 | 59927004 | Hepatic failure | SNOMED CT |
| Moderate or severe liver disease | 196455 | 51292008 | Hepatorenal syndrome | SNOMED CT |
| Moderate or severe liver disease | 192680 | 34742003 | Portal hypertension | SNOMED CT |
| Moderate or severe liver disease | 40481531 | 444918006 | Sequela of chronic liver disease | SNOMED CT |
| Moderate or severe liver disease | 4026136 | 197356006 | Toxic liver disease with hepatic necrosis | SNOMED CT |
| Moderate or severe liver disease | 4277276 | 65617004 | Veno-occlusive disease of the liver | SNOMED CT |
| Hemiplegia or paraplegia | 4102342 | 192970008 | Cauda equina syndrome | SNOMED CT |
| Hemiplegia or paraplegia | 193186 | 12454008 | Cauda equina syndrome with neurogenic bladder | SNOMED CT |
| Hemiplegia or paraplegia | 134331 | 8663007 | Cauda equina syndrome without neurogenic bladder | SNOMED CT |
| Hemiplegia or paraplegia | 4134120 | 128188000 | Cerebral palsy | SNOMED CT |
| Hemiplegia or paraplegia | 4104204 | 29188005 | Complete bilateral paralysis | SNOMED CT |
| Hemiplegia or paraplegia | 43530718 | 290401000119108 | Complete paraplegia | SNOMED CT |
| Hemiplegia or paraplegia | 40480416 | 441705005 | Complete tetraplegia due to lesion at C1-C4 level | SNOMED CT |
| Hemiplegia or paraplegia | 40481376 | 441980007 | Complete tetraplegia due to lesion at C5-C7 level | SNOMED CT |
| Hemiplegia or paraplegia | 4173811 | 275468009 | Congenital quadriplegia | SNOMED CT |
| Hemiplegia or paraplegia | 372880 | 54099005 | Diplegia of upper limbs | SNOMED CT |
| Hemiplegia or paraplegia | 132617 | 58193001 | Diplegic cerebral palsy | SNOMED CT |
| Hemiplegia or paraplegia | 372613 | 80935004 | Flaccid hemiplegia | SNOMED CT |
| Hemiplegia or paraplegia | 40481757 | 442020005 | Flaccid hemiplegia of dominant side | SNOMED CT |
| Hemiplegia or paraplegia | 40481820 | 442077006 | Flaccid hemiplegia of nondominant side | SNOMED CT |
| Hemiplegia or paraplegia | 4101738 | 192966000 | Flaccid paraplegia | SNOMED CT |
| Hemiplegia or paraplegia | 4104848 | 192964002 | Flaccid tetraplegia | SNOMED CT |
| Hemiplegia or paraplegia | 374022 | 50582007 | Hemiplegia | SNOMED CT |
| Hemiplegia or paraplegia | 40482237 | 442155009 | Hemiplegia of dominant side | SNOMED CT |
| Hemiplegia or paraplegia | 40480429 | 441717007 | Hemiplegia of nondominant side | SNOMED CT |
| Hemiplegia or paraplegia | 134031 | 43486001 | Hemiplegic cerebral palsy | SNOMED CT |
| Hemiplegia or paraplegia | 192901 | 39912006 | Hereditary spastic paraplegia | SNOMED CT |
| Hemiplegia or paraplegia | 37019108 | 714279000 | Human T-cell lymphotropic virus 1-associated myelopathy | SNOMED CT |
| Hemiplegia or paraplegia | 43530719 | 290411000119106 | Incomplete paraplegia | SNOMED CT |
| Hemiplegia or paraplegia | 40480514 | 441794001 | Incomplete tetraplegia due to lesion at C5-C7 level | SNOMED CT |
| Hemiplegia or paraplegia | 40480066 | 441688003 | Incomplete tetraplegia due to spinal cord lesion at C1-C4 level | SNOMED CT |
| Hemiplegia or paraplegia | 374336 | 1593000 | Infantile hemiplegia | SNOMED CT |
| Hemiplegia or paraplegia | 381548 | 86022000 | Monoplegia | SNOMED CT |
| Hemiplegia or paraplegia | 195240 | 80420005 | Monoplegia of lower limb | SNOMED CT |
| Hemiplegia or paraplegia | 4144328 | 426167003 | Monoplegia of lower limb affecting dominant side | SNOMED CT |
| Hemiplegia or paraplegia | 4141654 | 426934001 | Monoplegia of lower limb affecting non-dominant side | SNOMED CT |
| Hemiplegia or paraplegia | 379012 | 41764006 | Monoplegia of upper limb | SNOMED CT |
| Hemiplegia or paraplegia | 380393 | 426536006 | Monoplegia of upper limb affecting non-dominant side | SNOMED CT |
| Hemiplegia or paraplegia | 380393 | 426536006 | Monoplegia of upper limb affecting non-dominant side | SNOMED CT |
| Hemiplegia or paraplegia | 40481345 | 441951003 | Monoplegia of upper limb of dominant side | SNOMED CT |
| Hemiplegia or paraplegia | 442543 | 56409008 | Monoplegic cerebral palsy | SNOMED CT |
| Hemiplegia or paraplegia | 440377 | 44695005 | Paralysis | SNOMED CT |
| Hemiplegia or paraplegia | 374377 | 29426003 | Paralytic syndrome | SNOMED CT |
| Hemiplegia or paraplegia | 43531639 | 609557004 | Paralytic syndrome on one side of the body | SNOMED CT |
| Hemiplegia or paraplegia | 192606 | 60389000 | Paraplegia | SNOMED CT |
| Hemiplegia or paraplegia | 44782711 | 22881000119100 | Quadriplegia with quadriparesis | SNOMED CT |
| Hemiplegia or paraplegia | 375528 | 79633009 | Spastic hemiplegia | SNOMED CT |
| Hemiplegia or paraplegia | 40480944 | 441892008 | Spastic hemiplegia of dominant side | SNOMED CT |
| Hemiplegia or paraplegia | 40480435 | 441722007 | Spastic hemiplegia of nondominant side | SNOMED CT |
| Hemiplegia or paraplegia | 44806793 | 813921000000104 | Spastic hemiplegic cerebral palsy | SNOMED CT |
| Hemiplegia or paraplegia | 4101739 | 192967009 | Spastic paraplegia | SNOMED CT |
| Hemiplegia or paraplegia | 4102341 | 192965001 | Spastic tetraplegia | SNOMED CT |
| Hemiplegia or paraplegia | 374914 | 11538006 | Tetraplegia | SNOMED CT |
| Mild liver disease | 201343 | 9953008 | Acute alcoholic liver disease | SNOMED CT |
| Mild liver disease | 196463 | 420054005 | Alcoholic cirrhosis | SNOMED CT |
| Mild liver disease | 193256 | 50325005 | Alcoholic fatty liver | SNOMED CT |
| Mild liver disease | 4340385 | 235880004 | Alcoholic fibrosis and sclerosis of liver | SNOMED CT |
| Mild liver disease | 4340383 | 235875008 | Alcoholic hepatitis | SNOMED CT |
| Mild liver disease | 201612 | 41309000 | Alcoholic liver damage | SNOMED CT |
| Mild liver disease | 46269816 | 1082601000119100 | Ascites due to alcoholic cirrhosis | SNOMED CT |
| Mild liver disease | 200762 | 408335007 | Autoimmune hepatitis | SNOMED CT |
| Mild liver disease | 192675 | 1761006 | Biliary cirrhosis | SNOMED CT |
| Mild liver disease | 4058696 | 197364000 | Central hemorrhagic necrosis of liver | SNOMED CT |
| Mild liver disease | 4026125 | 197284004 | Chronic active hepatitis | SNOMED CT |
| Mild liver disease | 200763 | 76783007 | Chronic hepatitis | SNOMED CT |
| Mild liver disease | 198964 | 128302006 | Chronic hepatitis C | SNOMED CT |
| Mild liver disease | 4238978 | 57339008 | Chronic lobular hepatitis | SNOMED CT |
| Mild liver disease | 201613 | 79720007 | Chronic nonalcoholic liver disease | SNOMED CT |
| Mild liver disease | 199867 | 41889008 | Chronic persistent hepatitis | SNOMED CT |
| Mild liver disease | 4012113 | 10295004 | Chronic viral hepatitis | SNOMED CT |
| Mild liver disease | 192240 | 235869004 | Chronic viral hepatitis B with hepatitis D | SNOMED CT |
| Mild liver disease | 439674 | 186639003 | Chronic viral hepatitis B without delta-agent | SNOMED CT |
| Mild liver disease | 763021 | 435101000124104 | Chronic viral hepatitis C with hepatic coma | SNOMED CT |
| Mild liver disease | 194692 | 266468003 | Cirrhosis - non-alcoholic | SNOMED CT |
| Mild liver disease | 4059284 | 197279005 | Cirrhosis and chronic liver disease | SNOMED CT |
| Mild liver disease | 4064161 | 19943007 | Cirrhosis of liver | SNOMED CT |
| Mild liver disease | 194984 | 235856003 | Disease of liver | SNOMED CT |
| Mild liver disease | 46273476 | 1092801000119100 | Hepatic ascites co-occurrent with chronic active hepatitis due to toxic liver disease | SNOMED CT |
| Mild liver disease | 46269835 | 1085021000119100 | Hepatic ascites due to chronic alcoholic hepatitis | SNOMED CT |
| Mild liver disease | 439675 | 186624004 | Hepatic coma due to acute hepatitis B with delta agent | SNOMED CT |
| Mild liver disease | 4308946 | 424340000 | Hepatic coma due to chronic hepatitis B | SNOMED CT |
| Mild liver disease | 196029 | 40946000 | Hepatic coma due to viral hepatitis | SNOMED CT |
| Mild liver disease | 4267417 | 62484002 | Hepatic fibrosis | SNOMED CT |
| Mild liver disease | 4340948 | 235901004 | Hepatic fibrosis with hepatic sclerosis | SNOMED CT |
| Mild liver disease | 194417 | 17890003 | Hepatic infarction | SNOMED CT |
| Mild liver disease | 4337543 | 87248009 | Hepatic necrosis | SNOMED CT |
| Mild liver disease | 4340394 | 235899008 | Hepatic sclerosis | SNOMED CT |
| Mild liver disease | 4159144 | 371067004 | Hepatopulmonary syndrome | SNOMED CT |
| Mild liver disease | 194990 | 128241005 | Inflammatory disease of liver | SNOMED CT |
| Mild liver disease | 4240725 | 58008004 | Peliosis hepatis | SNOMED CT |
| Mild liver disease | 4135822 | 31712002 | Primary biliary cholangitis | SNOMED CT |
| Mild liver disease | 4046123 | 12368000 | Secondary biliary cirrhosis | SNOMED CT |
| Mild liver disease | 4059290 | 197321007 | Steatosis of liver | SNOMED CT |
| Mild liver disease | 4059299 | 197361008 | Toxic liver disease with chronic active hepatitis | SNOMED CT |
| Mild liver disease | 4059298 | 197359004 | Toxic liver disease with chronic persistent hepatitis | SNOMED CT |
| Mild liver disease | 4058695 | 197362001 | Toxic liver disease with fibrosis and cirrhosis of liver | SNOMED CT |
| Mild liver disease | 42537742 | 737297006 | Transplanted liver present | SNOMED CT |
| Mild liver disease | 198683 | 111891008 | Viral hepatitis B without hepatic coma | SNOMED CT |
| Mild liver disease | 193693 | 111896003 | Viral hepatitis without hepatic coma | SNOMED CT |
| Peptic ulcer disease | 4027729 | 12847006 | Acute duodenal ulcer with hemorrhage | SNOMED CT |
| Peptic ulcer disease | 441062 | 87756006 | Acute duodenal ulcer with hemorrhage AND obstruction | SNOMED CT |
| Peptic ulcer disease | 4336230 | 86895006 | Acute duodenal ulcer with hemorrhage AND perforation | SNOMED CT |
| Peptic ulcer disease | 435855 | 51847008 | Acute duodenal ulcer with hemorrhage AND with perforation but without obstruction | SNOMED CT |
| Peptic ulcer disease | 434402 | 66767006 | Acute duodenal ulcer with hemorrhage but without obstruction | SNOMED CT |
| Peptic ulcer disease | 437021 | 41986000 | Acute duodenal ulcer with hemorrhage, with perforation AND with obstruction | SNOMED CT |
| Peptic ulcer disease | 4265479 | 61347001 | Acute duodenal ulcer with perforation | SNOMED CT |
| Peptic ulcer disease | 434070 | 62936002 | Acute duodenal ulcer with perforation AND obstruction | SNOMED CT |
| Peptic ulcer disease | 435578 | 22511002 | Acute duodenal ulcer with perforation but without obstruction | SNOMED CT |
| Peptic ulcer disease | 4138962 | 32490005 | Acute duodenal ulcer without hemorrhage AND without perforation | SNOMED CT |
| Peptic ulcer disease | 435859 | 75342000 | Acute duodenal ulcer without hemorrhage AND without perforation but with obstruction | SNOMED CT |
| Peptic ulcer disease | 440755 | 23693000 | Acute duodenal ulcer without hemorrhage, without perforation AND without obstruction | SNOMED CT |
| Peptic ulcer disease | 4231580 | 89748001 | Acute gastric ulcer with hemorrhage | SNOMED CT |
| Peptic ulcer disease | 198467 | 46708007 | Acute gastric ulcer with hemorrhage and obstruction | SNOMED CT |
| Peptic ulcer disease | 4169592 | 48974009 | Acute gastric ulcer with hemorrhage and perforation | SNOMED CT |
| Peptic ulcer disease | 199855 | 17067009 | Acute gastric ulcer with hemorrhage AND with perforation but without obstruction | SNOMED CT |
| Peptic ulcer disease | 193795 | 70418001 | Acute gastric ulcer with hemorrhage but without obstruction | SNOMED CT |
| Peptic ulcer disease | 195845 | 53337006 | Acute gastric ulcer with hemorrhage, with perforation and with obstruction | SNOMED CT |
| Peptic ulcer disease | 4057953 | 19850005 | Acute gastric ulcer with perforation | SNOMED CT |
| Peptic ulcer disease | 200137 | 43694004 | Acute gastric ulcer with perforation AND obstruction | SNOMED CT |
| Peptic ulcer disease | 194680 | 90628007 | Acute gastric ulcer with perforation but without obstruction | SNOMED CT |
| Peptic ulcer disease | 4195231 | 67964002 | Acute gastric ulcer without hemorrhage AND without perforation | SNOMED CT |
| Peptic ulcer disease | 192954 | 81225008 | Acute gastric ulcer without hemorrhage AND without perforation but with obstruction | SNOMED CT |
| Peptic ulcer disease | 199062 | 54053008 | Acute gastric ulcer without hemorrhage, without perforation AND without obstruction | SNOMED CT |
| Peptic ulcer disease | 4274491 | 63954007 | Acute gastrojejunal ulcer with hemorrhage | SNOMED CT |
| Peptic ulcer disease | 441063 | 72408002 | Acute gastrojejunal ulcer with hemorrhage and obstruction | SNOMED CT |
| Peptic ulcer disease | 4217947 | 81387001 | Acute gastrojejunal ulcer with hemorrhage and perforation | SNOMED CT |
| Peptic ulcer disease | 441328 | 66673003 | Acute gastrojejunal ulcer with hemorrhage and with perforation but without obstruction | SNOMED CT |
| Peptic ulcer disease | 438468 | 59515005 | Acute gastrojejunal ulcer with hemorrhage but without obstruction | SNOMED CT |
| Peptic ulcer disease | 442314 | 58711008 | Acute gastrojejunal ulcer with hemorrhage, with perforation and with obstruction | SNOMED CT |
| Peptic ulcer disease | 4280942 | 66636001 | Acute gastrojejunal ulcer with perforation | SNOMED CT |
| Peptic ulcer disease | 435846 | 72219001 | Acute gastrojejunal ulcer with perforation AND obstruction | SNOMED CT |
| Peptic ulcer disease | 437598 | 72395008 | Acute gastrojejunal ulcer with perforation but without obstruction | SNOMED CT |
| Peptic ulcer disease | 4147683 | 30514008 | Acute gastrojejunal ulcer without hemorrhage AND without perforation | SNOMED CT |
| Peptic ulcer disease | 432951 | 10389003 | Acute gastrojejunal ulcer without hemorrhage AND without perforation but with obstruction | SNOMED CT |
| Peptic ulcer disease | 436460 | 77987006 | Acute gastrojejunal ulcer without hemorrhage, without perforation AND without obstruction | SNOMED CT |
| Peptic ulcer disease | 4046500 | 12274003 | Acute peptic ulcer with hemorrhage | SNOMED CT |
| Peptic ulcer disease | 23237 | 43406003 | Acute peptic ulcer with hemorrhage AND obstruction | SNOMED CT |
| Peptic ulcer disease | 4006994 | 111353003 | Acute peptic ulcer with hemorrhage and perforation | SNOMED CT |
| Peptic ulcer disease | 27026 | 47064007 | Acute peptic ulcer with hemorrhage AND with perforation but without obstruction | SNOMED CT |
| Peptic ulcer disease | 31335 | 22157005 | Acute peptic ulcer with hemorrhage but without obstruction | SNOMED CT |
| Peptic ulcer disease | 194986 | 28945005 | Acute peptic ulcer with hemorrhage, with perforation AND with obstruction | SNOMED CT |
| Peptic ulcer disease | 4194543 | 79118000 | Acute peptic ulcer with perforation | SNOMED CT |
| Peptic ulcer disease | 26718 | 35681000 | Acute peptic ulcer with perforation AND obstruction | SNOMED CT |
| Peptic ulcer disease | 30439 | 34921009 | Acute peptic ulcer with perforation but without obstruction | SNOMED CT |
| Peptic ulcer disease | 4163865 | 45485004 | Acute peptic ulcer without hemorrhage AND without perforation | SNOMED CT |
| Peptic ulcer disease | 195584 | 58085004 | Acute peptic ulcer without hemorrhage AND without perforation but with obstruction | SNOMED CT |
| Peptic ulcer disease | 198187 | 3023008 | Acute peptic ulcer without hemorrhage, without perforation AND without obstruction | SNOMED CT |
| Peptic ulcer disease | 4232181 | 89469000 | Chronic duodenal ulcer with hemorrhage | SNOMED CT |
| Peptic ulcer disease | 437323 | 34021006 | Chronic duodenal ulcer with hemorrhage AND obstruction | SNOMED CT |
| Peptic ulcer disease | 4289830 | 36975000 | Chronic duodenal ulcer with hemorrhage AND perforation | SNOMED CT |
| Peptic ulcer disease | 438796 | 81142005 | Chronic duodenal ulcer with hemorrhage AND with perforation but without obstruction | SNOMED CT |
| Peptic ulcer disease | 436148 | 62341002 | Chronic duodenal ulcer with hemorrhage but without obstruction | SNOMED CT |
| Peptic ulcer disease | 440756 | 86258000 | Chronic duodenal ulcer with hemorrhage, with perforation AND with obstruction | SNOMED CT |
| Peptic ulcer disease | 4173408 | 49916007 | Chronic duodenal ulcer with perforation | SNOMED CT |
| Peptic ulcer disease | 439058 | 60551006 | Chronic duodenal ulcer with perforation AND obstruction | SNOMED CT |
| Peptic ulcer disease | 432354 | 34602004 | Chronic duodenal ulcer with perforation but without obstruction | SNOMED CT |
| Peptic ulcer disease | 4222896 | 40214005 | Chronic duodenal ulcer without hemorrhage AND without perforation | SNOMED CT |
| Peptic ulcer disease | 443770 | 28082003 | Chronic duodenal ulcer without hemorrhage AND without perforation but with obstruction | SNOMED CT |
| Peptic ulcer disease | 433246 | 57940000 | Chronic duodenal ulcer without hemorrhage, without perforation AND without obstruction | SNOMED CT |
| Peptic ulcer disease | 4211001 | 57246001 | Chronic gastric ulcer with hemorrhage | SNOMED CT |
| Peptic ulcer disease | 201885 | 85859006 | Chronic gastric ulcer with hemorrhage and with obstruction | SNOMED CT |
| Peptic ulcer disease | 4294973 | 76181002 | Chronic gastric ulcer with hemorrhage and with perforation | SNOMED CT |
| Peptic ulcer disease | 196442 | 74341002 | Chronic gastric ulcer with hemorrhage AND with perforation but without obstruction | SNOMED CT |
| Peptic ulcer disease | 197018 | 76078009 | Chronic gastric ulcer with hemorrhage but without obstruction | SNOMED CT |
| Peptic ulcer disease | 198801 | 85787009 | Chronic gastric ulcer with hemorrhage, with perforation and with obstruction | SNOMED CT |
| Peptic ulcer disease | 4150681 | 31301004 | Chronic gastric ulcer with perforation | SNOMED CT |
| Peptic ulcer disease | 4206315 | 55483002 | Chronic gastric ulcer with perforation AND with obstruction | SNOMED CT |
| Peptic ulcer disease | 197914 | 36246001 | Chronic gastric ulcer with perforation but without obstruction | SNOMED CT |
| Peptic ulcer disease | 4296611 | 76796008 | Chronic gastric ulcer without hemorrhage AND without perforation | SNOMED CT |
| Peptic ulcer disease | 195583 | 60531007 | Chronic gastric ulcer without hemorrhage AND without perforation but with obstruction | SNOMED CT |
| Peptic ulcer disease | 200769 | 1567007 | Chronic gastric ulcer without hemorrhage, without perforation AND without obstruction | SNOMED CT |
| Peptic ulcer disease | 433515 | 62838000 | Chronic gastrojejunal ulcer with hemorrhage | SNOMED CT |
| Peptic ulcer disease | 436729 | 90257004 | Chronic gastrojejunal ulcer with hemorrhage and obstruction | SNOMED CT |
| Peptic ulcer disease | 4164920 | 45640006 | Chronic gastrojejunal ulcer with hemorrhage and perforation | SNOMED CT |
| Peptic ulcer disease | 437326 | 46523000 | Chronic gastrojejunal ulcer with hemorrhage and with perforation but without obstruction | SNOMED CT |
| Peptic ulcer disease | 443779 | 24001002 | Chronic gastrojejunal ulcer with hemorrhage, with perforation and with obstruction | SNOMED CT |
| Peptic ulcer disease | 4101870 | 2807004 | Chronic gastrojejunal ulcer with perforation | SNOMED CT |
| Peptic ulcer disease | 444102 | 10897002 | Chronic gastrojejunal ulcer with perforation AND with obstruction | SNOMED CT |
| Peptic ulcer disease | 435579 | 62477005 | Chronic gastrojejunal ulcer with perforation but without obstruction | SNOMED CT |
| Peptic ulcer disease | 4177387 | 4269005 | Chronic gastrojejunal ulcer without hemorrhage AND without perforation | SNOMED CT |
| Peptic ulcer disease | 434400 | 56579005 | Chronic gastrojejunal ulcer without hemorrhage AND without perforation but with obstruction | SNOMED CT |
| Peptic ulcer disease | 438795 | 41626001 | Chronic gastrojejunal ulcer without hemorrhage, without perforation AND without obstruction | SNOMED CT |
| Peptic ulcer disease | 4174044 | 49232000 | Chronic peptic ulcer with hemorrhage | SNOMED CT |
| Peptic ulcer disease | 24076 | 56461008 | Chronic peptic ulcer with hemorrhage AND obstruction | SNOMED CT |
| Peptic ulcer disease | 4247008 | 61300005 | Chronic peptic ulcer with hemorrhage AND perforation | SNOMED CT |
| Peptic ulcer disease | 22665 | 55746001 | Chronic peptic ulcer with hemorrhage AND with perforation but without obstruction | SNOMED CT |
| Peptic ulcer disease | 30770 | 81518000 | Chronic peptic ulcer with hemorrhage but without obstruction | SNOMED CT |
| Peptic ulcer disease | 24397 | 77661009 | Chronic peptic ulcer with hemorrhage, with perforation AND with obstruction | SNOMED CT |
| Peptic ulcer disease | 4146517 | 3483000 | Chronic peptic ulcer with perforation | SNOMED CT |
| Peptic ulcer disease | 30442 | 57871005 | Chronic peptic ulcer with perforation AND obstruction | SNOMED CT |
| Peptic ulcer disease | 23247 | 80953005 | Chronic peptic ulcer with perforation but without obstruction | SNOMED CT |
| Peptic ulcer disease | 4204555 | 5492000 | Chronic peptic ulcer without hemorrhage AND without perforation | SNOMED CT |
| Peptic ulcer disease | 24973 | 12384004 | Chronic peptic ulcer without hemorrhage AND without perforation but with obstruction | SNOMED CT |
| Peptic ulcer disease | 23808 | 60400003 | Chronic peptic ulcer without hemorrhage, without perforation AND without obstruction | SNOMED CT |
| Peptic ulcer disease | 4031954 | 23812009 | Duodenal ulcer with hemorrhage AND perforation | SNOMED CT |
| Peptic ulcer disease | 4209746 | 56776001 | Duodenal ulcer without hemorrhage AND without perforation | SNOMED CT |
| Peptic ulcer disease | 435305 | 18169007 | Duodenal ulcer without hemorrhage AND without perforation but with obstruction | SNOMED CT |
| Peptic ulcer disease | 438469 | 34580000 | Duodenal ulcer without hemorrhage, without perforation AND without obstruction | SNOMED CT |
| Peptic ulcer disease | 4265600 | 397825006 | Gastric ulcer | SNOMED CT |
| Peptic ulcer disease | 4248429 | 73481001 | Gastric ulcer without hemorrhage AND without perforation | SNOMED CT |
| Peptic ulcer disease | 196443 | 31452001 | Gastric ulcer without hemorrhage AND without perforation but with obstruction | SNOMED CT |
| Peptic ulcer disease | 195851 | 59913009 | Gastric ulcer without hemorrhage, without perforation AND without obstruction | SNOMED CT |
| Peptic ulcer disease | 4059178 | 16121001 | Gastrojejunal ulcer | SNOMED CT |
| Peptic ulcer disease | 4101104 | 2783007 | Gastrojejunal ulcer without hemorrhage AND without perforation | SNOMED CT |
| Peptic ulcer disease | 438188 | 47152002 | Gastrojejunal ulcer without hemorrhage AND without perforation but with obstruction | SNOMED CT |
| Peptic ulcer disease | 439858 | 35517004 | Gastrojejunal ulcer without hemorrhage, without perforation AND without obstruction | SNOMED CT |
| Peptic ulcer disease | 4027663 | 13200003 | Peptic ulcer | SNOMED CT |
| Peptic ulcer disease | 4291028 | 37442009 | Peptic ulcer without hemorrhage AND without perforation | SNOMED CT |
| Peptic ulcer disease | 200771 | 54157007 | Peptic ulcer without hemorrhage AND without perforation but with obstruction | SNOMED CT |
| Peptic ulcer disease | 201069 | 38365000 | Peptic ulcer without hemorrhage, without perforation AND without obstruction | SNOMED CT |
| Peptic ulcer disease | 4198381 | 51868009 | Ulcer of duodenum | SNOMED CT |
| Rheumatic disease | 4344166 | 239920006 | Adult onset Still's disease | SNOMED CT |
| Rheumatic disease | 4005037 | 1212005 | Childhood type dermatomyositis | SNOMED CT |
| Rheumatic disease | 4135937 | 31848007 | CREST syndrome | SNOMED CT |
| Rheumatic disease | 80182 | 396230008 | Dermatomyositis | SNOMED CT |
| Rheumatic disease | 4081250 | 238935002 | Dermatomyositis sine myositis | SNOMED CT |
| Rheumatic disease | 4344161 | 239901009 | Dermatomyositis with malignant disease | SNOMED CT |
| Rheumatic disease | 4063581 | 201436003 | Drug-induced systemic lupus erythematosus | SNOMED CT |
| Rheumatic disease | 46273369 | 72181000119109 | Endocarditis due to systemic lupus erythematosus | SNOMED CT |
| Rheumatic disease | 81097 | 57160007 | Felty's syndrome | SNOMED CT |
| Rheumatic disease | 4343935 | 239938009 | Giant cell arteritis with polymyalgia rheumatica | SNOMED CT |
| Rheumatic disease | 74125 | 417373000 | Inflammatory polyarthropathy | SNOMED CT |
| Rheumatic disease | 37395588 | 311671000119100 | Juvenile dermatomyositis co-occurrent with respiratory involvement | SNOMED CT |
| Rheumatic disease | 4055369 | 196136009 | Lung disease with polymyositis | SNOMED CT |
| Rheumatic disease | 4055640 | 196138005 | Lung disease with systemic lupus erythematosus | SNOMED CT |
| Rheumatic disease | 255304 | 196133001 | Lung disease with systemic sclerosis | SNOMED CT |
| Rheumatic disease | 4107913 | 193250002 | Myopathy due to rheumatoid arthritis | SNOMED CT |
| Rheumatic disease | 4105026 | 193252005 | Myopathy due to systemic sclerosis | SNOMED CT |
| Rheumatic disease | 4079978 | 276657008 | Overlap syndrome | SNOMED CT |
| Rheumatic disease | 255348 | 65323003 | Polymyalgia rheumatica | SNOMED CT |
| Rheumatic disease | 80800 | 31384009 | Polymyositis | SNOMED CT |
| Rheumatic disease | 46270482 | 322461000119108 | Polyneuropathy due to systemic sclerosis | SNOMED CT |
| Rheumatic disease | 4102493 | 193180002 | Polyneuropathy in rheumatoid arthritis | SNOMED CT |
| Rheumatic disease | 40485046 | 444133002 | Progressive systemic sclerosis | SNOMED CT |
| Rheumatic disease | 4145240 | 307755009 | Renal tubulo-interstitial disorder in systemic lupus erythematosus | SNOMED CT |
| Rheumatic disease | 80809 | 69896004 | Rheumatoid arthritis | SNOMED CT |
| Rheumatic disease | 4117687 | 287008006 | Rheumatoid arthritis - ankle and/or foot | SNOMED CT |
| Rheumatic disease | 4115161 | 287007001 | Rheumatoid arthritis - hand joint | SNOMED CT |
| Rheumatic disease | 4116440 | 201769002 | Rheumatoid arthritis of elbow | SNOMED CT |
| Rheumatic disease | 4116150 | 201775006 | Rheumatoid arthritis of hip | SNOMED CT |
| Rheumatic disease | 4116151 | 201777003 | Rheumatoid arthritis of knee | SNOMED CT |
| Rheumatic disease | 42534834 | 1073711000119100 | Rheumatoid arthritis of left hand | SNOMED CT |
| Rheumatic disease | 42534835 | 1073721000119100 | Rheumatoid arthritis of left hip | SNOMED CT |
| Rheumatic disease | 37108590 | 1073731000119100 | Rheumatoid arthritis of left knee | SNOMED CT |
| Rheumatic disease | 35609009 | 1073741000119100 | Rheumatoid arthritis of left shoulder | SNOMED CT |
| Rheumatic disease | 36685020 | 1073751000119100 | Rheumatoid arthritis of left wrist | SNOMED CT |
| Rheumatic disease | 4117686 | 287006005 | Rheumatoid arthritis of multiple joints | SNOMED CT |
| Rheumatic disease | 36685022 | 1073771000119100 | Rheumatoid arthritis of right elbow | SNOMED CT |
| Rheumatic disease | 42534836 | 1073791000119100 | Rheumatoid arthritis of right hand | SNOMED CT |
| Rheumatic disease | 42534837 | 1073801000119100 | Rheumatoid arthritis of right hip | SNOMED CT |
| Rheumatic disease | 37108591 | 1073811000119100 | Rheumatoid arthritis of right knee | SNOMED CT |
| Rheumatic disease | 35609010 | 1073821000119100 | Rheumatoid arthritis of right shoulder | SNOMED CT |
| Rheumatic disease | 36685024 | 1073831000119100 | Rheumatoid arthritis of right wrist | SNOMED CT |
| Rheumatic disease | 4114439 | 201766009 | Rheumatoid arthritis of shoulder | SNOMED CT |
| Rheumatic disease | 4116441 | 201771002 | Rheumatoid arthritis of wrist | SNOMED CT |
| Rheumatic disease | 36684997 | 459911000124100 | Rheumatoid factor positive rheumatoid arthritis | SNOMED CT |
| Rheumatic disease | 256197 | 398726004 | Rheumatoid lung disease | SNOMED CT |
| Rheumatic disease | 37395590 | 319841000119107 | Rheumatoid lung disease with rheumatoid arthritis | SNOMED CT |
| Rheumatic disease | 4162539 | 398640008 | Rheumatoid pneumoconiosis | SNOMED CT |
| Rheumatic disease | 4271003 | 400054000 | Rheumatoid vasculitis | SNOMED CT |
| Rheumatic disease | 4083556 | 239792003 | Seronegative rheumatoid arthritis | SNOMED CT |
| Rheumatic disease | 4035611 | 239791005 | Seropositive rheumatoid arthritis | SNOMED CT |
| Rheumatic disease | 254443 | 83901003 | Sjögren's syndrome | SNOMED CT |
| Rheumatic disease | 4285717 | 68815009 | SLE glomerulonephritis syndrome | SNOMED CT |
| Rheumatic disease | 4142899 | 33719002 | Subcutaneous rheumatoid nodule | SNOMED CT |
| Rheumatic disease | 257628 | 55464009 | Systemic lupus erythematosus | SNOMED CT |
| Rheumatic disease | 4344158 | 239887007 | Systemic lupus erythematosus with organ/system involvement | SNOMED CT |
| Rheumatic disease | 4149913 | 309762007 | Systemic lupus erythematosus with pericarditis | SNOMED CT |
| Rheumatic disease | 134442 | 89155008 | Systemic sclerosis | SNOMED CT |
| Rheumatic disease | 4063582 | 201443009 | Systemic sclerosis induced by drugs and chemicals | SNOMED CT |
| Rheumatic disease | 4290976 | 400130008 | Temporal arteritis | SNOMED CT |
| Myocardial infarction | 45766075 | 703164000 | Acute anterior ST segment elevation myocardial infarction | SNOMED CT |
| Myocardial infarction | 312327 | 57054005 | Acute myocardial infarction | SNOMED CT |
| Myocardial infarction | 434376 | 54329005 | Acute myocardial infarction of anterior wall | SNOMED CT |
| Myocardial infarction | 438438 | 70211005 | Acute myocardial infarction of anterolateral wall | SNOMED CT |
| Myocardial infarction | 438170 | 73795002 | Acute myocardial infarction of inferior wall | SNOMED CT |
| Myocardial infarction | 438447 | 65547006 | Acute myocardial infarction of inferolateral wall | SNOMED CT |
| Myocardial infarction | 441579 | 76593002 | Acute myocardial infarction of inferoposterior wall | SNOMED CT |
| Myocardial infarction | 436706 | 58612006 | Acute myocardial infarction of lateral wall | SNOMED CT |
| Myocardial infarction | 4270024 | 401314000 | Acute non-ST segment elevation myocardial infarction | SNOMED CT |
| Myocardial infarction | 4296653 | 401303003 | Acute ST segment elevation myocardial infarction | SNOMED CT |
| Myocardial infarction | 46270162 | 15713081000119100 | Acute ST segment elevation myocardial infarction due to left coronary artery occlusion | SNOMED CT |
| Myocardial infarction | 46270163 | 15713121000119100 | Acute ST segment elevation myocardial infarction due to right coronary artery occlusion | SNOMED CT |
| Myocardial infarction | 43020460 | 285981000119103 | Acute ST segment elevation myocardial infarction involving left anterior descending coronary artery | SNOMED CT |
| Myocardial infarction | 45766116 | 703213009 | Acute ST segment elevation myocardial infarction of inferior wall | SNOMED CT |
| Myocardial infarction | 444406 | 70422006 | Acute subendocardial infarction | SNOMED CT |
| Myocardial infarction | 4329847 | 22298006 | Myocardial infarction | SNOMED CT |
| Myocardial infarction | 37309626 | 16837681000119100 | Myocardial infarction due to demand ischemia | SNOMED CT |
| Myocardial infarction | 314666 | 1755008 | Old myocardial infarction | SNOMED CT |
| Myocardial infarction | 4108217 | 194856005 | Subsequent myocardial infarction | SNOMED CT |
| Myocardial infarction | 4108677 | 194857001 | Subsequent myocardial infarction of anterior wall | SNOMED CT |
| Myocardial infarction | 4108218 | 194858006 | Subsequent myocardial infarction of inferior wall | SNOMED CT |
| Myocardial infarction | 45766241 | 703360004 | Subsequent non-ST segment elevation myocardial infarction | SNOMED CT |
| Myocardial infarction | 45766114 | 703211006 | Subsequent ST segment elevation myocardial infarction | SNOMED CT |
| Myocardial infarction | 439693 | 194802003 | True posterior myocardial infarction | SNOMED CT |
| Dementia | 378419 | 26929004 | Alzheimer's disease | SNOMED CT |
| Dementia | 37111242 | 725898002 | Delirium co-occurrent with dementia | SNOMED CT |
| Dementia | 44782726 | 141991000119109 | Delusions in Alzheimer's disease | SNOMED CT |
| Dementia | 4182210 | 52448006 | Dementia | SNOMED CT |
| Dementia | 4228133 | 421529006 | Dementia associated with AIDS | SNOMED CT |
| Dementia | 374888 | 191519005 | Dementia associated with another disease | SNOMED CT |
| Dementia | 4314734 | 425390006 | Dementia associated with Parkinson's Disease | SNOMED CT |
| Dementia | 4180284 | 429458009 | Dementia due to Creutzfeldt Jakob disease | SNOMED CT |
| Dementia | 40483103 | 442344002 | Dementia due to Huntington chorea | SNOMED CT |
| Dementia | 44782422 | 101421000119107 | Dementia due to Parkinson's disease | SNOMED CT |
| Dementia | 44782710 | 21921000119103 | Dementia due to Pick's disease | SNOMED CT |
| Dementia | 43530666 | 1591000119103 | Dementia with behavioral disturbance | SNOMED CT |
| Dementia | 44782727 | 142001000119106 | Depressed mood in Alzheimer's disease | SNOMED CT |
| Dementia | 37017319 | 713571008 | Disorder of central nervous system co-occurrent with human immunodeficiency virus infection | SNOMED CT |
| Dementia | 36687122 | 15928141000119100 | Human immunodeficiency virus infection with cognitive impairment | SNOMED CT |
| Dementia | 4046090 | 230287006 | Mixed cortical and subcortical vascular dementia | SNOMED CT |
| Dementia | 379778 | 56267009 | Multi-infarct dementia | SNOMED CT |
| Dementia | 444091 | 10349009 | Multi-infarct dementia with delirium | SNOMED CT |
| Dementia | 443790 | 25772007 | Multi-infarct dementia with delusions | SNOMED CT |
| Dementia | 443864 | 14070001 | Multi-infarct dementia with depression | SNOMED CT |
| Dementia | 377254 | 70936005 | Multi-infarct dementia, uncomplicated | SNOMED CT |
| Dementia | 378125 | 12348006 | Presenile dementia | SNOMED CT |
| Dementia | 381832 | 191452002 | Presenile dementia with delirium | SNOMED CT |
| Dementia | 44782771 | 31081000119101 | Presenile dementia with delusions | SNOMED CT |
| Dementia | 377527 | 191455000 | Presenile dementia with depression | SNOMED CT |
| Dementia | 4218017 | 416780008 | Primary degenerative dementia of the Alzheimer type, presenile onset | SNOMED CT |
| Dementia | 4220313 | 416975007 | Primary degenerative dementia of the Alzheimer type, senile onset | SNOMED CT |
| Dementia | 4152048 | 268612007 | Senile and presenile organic psychotic conditions | SNOMED CT |
| Dementia | 373179 | 45864009 | Senile degeneration of brain | SNOMED CT |
| Dementia | 4048875 | 15662003 | Senile dementia | SNOMED CT |
| Dementia | 376946 | 191461002 | Senile dementia with delirium | SNOMED CT |
| Dementia | 380986 | 371024007 | Senile dementia with delusion | SNOMED CT |
| Dementia | 379784 | 191459006 | Senile dementia with depression | SNOMED CT |
| Dementia | 4047747 | 230286002 | Subcortical vascular dementia | SNOMED CT |
| Dementia | 376085 | 191451009 | Uncomplicated presenile dementia | SNOMED CT |
| Dementia | 375791 | 191449005 | Uncomplicated senile dementia | SNOMED CT |
| Dementia | 443605 | 429998004 | Vascular dementia | SNOMED CT |
| Dementia | 4046089 | 230285003 | Vascular dementia of acute onset | SNOMED CT |
| Dementia | 37018688 | 288631000119104 | Vascular dementia with behavioral disturbance | SNOMED CT |
| Dementia | 37109056 | 16276361000119100 | Vascular dementia without behavioral disturbance | SNOMED CT |

**Supplemental Appendix B.** Performance of Charlson comorbidity index (CCI) coding algorithms to predict one-year mortality

| **Model** | **Dependent Variables** | **2013, c-statistic (95% CI)** | | **2018, c-statistic (95% CI)** | |
| --- | --- | --- | --- | --- | --- |
|  |  | **MDCD** | **DOD** | **MDCD** | **DOD** |
| 1 | CCI (SNOMED CT) | 0.725 (0.721, 0.728) | 0.789 (0.787, 0.79) | 0.754 (0.751, 0.757) | 0.757 (0.755, 0.758) |
| 2 | CCI (Quan) | 0.723 (0.72, 0.726) | 0.787 (0.786, 0.789) | 0.752 (0.749, 0.754) | 0.757 (0.756, 0.758) |
