# Supplementary material for: Adaptation and validation of a coding algorithm for the Charlson Comorbidity Index in administrative claims data using the SNOMED CT standardized vocabulary
Source: BMC Med Inform Decis Mak. 2022 Oct 7;22:261. doi: 10.1186/s12911-022-02006-1 (PMC9541054; doi:10.1186/s12911-022-02006-1)
Supplement: Supplementary file 2 — Additional file 2. Review history. [file 12911_2022_2006_MOESM2_ESM.zip › Peer review - af18d343-ce7c-4eaa-8d1c-d14271508d91 - reviewer 1.pdf]

15-JUN-22

Perform the analysis by segmenting the data by age and gender
