# Supplementary material for: Adaptation and validation of a coding algorithm for the Charlson Comorbidity Index in administrative claims data using the SNOMED CT standardized vocabulary
Source: BMC Med Inform Decis Mak. 2022 Oct 7;22:261. doi: 10.1186/s12911-022-02006-1 (PMC9541054; doi:10.1186/s12911-022-02006-1)
Supplement: Supplementary file 2 — Additional file 2. Review history. [file 12911_2022_2006_MOESM2_ESM.zip › Peer review - af18d343-ce7c-4eaa-8d1c-d14271508d91 - reviewer 2.pdf]

29-JUN-22

The authors present a study that utilized SNOMED CT in lieu of ICD-9/10 for calculation of the Charlson comorbidity index. Analyses include two large administrative claims databases. Results demonstrate that use of SNOMED CT as the base code system for Charlson comorbitiy calculations are statistically equivalent to calculations using the ICD system. This work is promising as SNOMED CT is a prevalent terminology system used internationally within the electronic health records and for public health/translational research in large, federated, observational data warehouses. Thus, these datasets and SNOMED CT basis can be used reliably in the context of comorbidity evaluations.

The authors' methods are sound, and there are some items that would be beneficial to be addressed prior to acceptance.

1. The ICD system has its origins in epidemiology and tracking morbidity while SNOMED CT finds its origins in data analytics. As a result, the constructs of each terminology are different. ICD is a taxonomy while SNOMED CT is an onotology, more specifically, SNOMED CT supports polyhierarchy and ICD does not. Therefore, an ICD code indicates a specific, singular concept for a morbid condition while the SNOMED CT concept can indicate multiple "vectors" for morbidity. For example, infectious pneumonia is a disease of the lung in ICD. In SNOMED CT it is a disease of the lung, an infectious disease, and potentially a disease caused by a particular organism.
Readers would benefit from this difference between terminologies, and the authors would add value to their evaluation by articulating how this specific difference impacts their analysis. This could be an improvement in terms of specificity and/or aggregation of comorbid conditions. How did the authors address polyhierarchy in their analysis?

2. The authors discuss "gain" in the use of SNOMED CT for these calculations. Could the authors elaborate on that observation and discuss its pros/cons.
3. ICD allows for "Not otherwise specified" and SNOMED CT does not. This is a classic difference between an Open World vs. Closed World view in terminologies. How do the authors handle/address this?
4. Finally, SNOMED CT publishes new releases every 6 months for the International community. This is much more frequent than the ICD system. The authors should specify the specific SNOMED CT releases used for their analyses and discuss its effect on the results.

This comments are fairly broad and merit some level of discussion as the authors are comfortable and for which there is space in the manuscript.
